# Effects, Acceptability, and Use of a Dynamically Tailored Mobile What Do You Drink Intervention to Reduce Excessive Drinking Among Adolescents and Young Adults in the Netherlands: Randomized Controlled Trial

**DOI:** 10.2196/68468

**Published:** 2026-05-26

**Authors:** Hilde van Keulen, Carmen Voogt, Marloes Kleinjan, Arjan Huizing, Rosa Andree, Pepijn van Empelen

**Affiliations:** 1 Department of Child Health, Netherlands Organisation for Applied Scientific Research, Sylviusweg 71, Leiden, 2333 BE, The Netherlands, 31 652803631; 2 Eating Disorder Experts Network, Emergis and Parnassia Groep, The Hague, The Netherlands; 3 HAN (Hogeschool Arnhem Nijmegen) University of Applied Sciences, Research Group Organisation of Care and Service Delivery, Arnhem, The Netherlands; 4 Drug Monitoring and Policy, Trimbos Institute, Utrecht, The Netherlands; 5 Department of Health, Medical and Neuropsychology, University of Leiden, Leiden, The Netherlands

**Keywords:** alcohol consumption, excessive drinking, at-risk population, adolescents, young adults, dynamic tailoring, mHealth intervention, randomized controlled trial, health promotion

## Abstract

**Background:**

Excessive alcohol consumption among adolescents and young adults is a serious health problem. Dynamically tailored interventions could reduce their excessive drinking. We therefore developed “What Do You Drink” (WDYD), a 17-week dynamically tailored mHealth (mobile health) intervention providing personalized support on alcohol consumption.

**Objective:**

We aim to evaluate the effectiveness, acceptability, and use of WDYD in reducing alcohol consumption of adolescents and young adults at risk.

**Methods:**

We conducted a 2-arm, parallel-group randomized controlled trial using ecological momentary assessments. Recruitment was via an educational alcohol program, an online lifestyle monitor, social media advertisements, or news items on websites. Participants downloaded the standalone WDYD app, and when having given active informed consent, were randomized to the intervention or control group. Participants in the intervention group received dynamically tailored feedback sessions on alcohol consumption (wk 0‐5, 7, 9, 13, and 17) and goal-monitoring reminders. Both groups completed an online baseline survey, 2 follow-up surveys (wk 9 and 33), and various ecological momentary assessments (7 daily assessments during wk 1, 7, 13, 19, 25, 31, and 33). Participants provided consent before randomization, in which they were informed that 2 study groups existed. After randomization, no disclosure of group assignment was provided, although participants could potentially infer it from receiving tailored sessions vs no tailored sessions. Primary outcomes were excessive drinking, binge drinking, and weekly alcohol consumption. Secondary outcomes were intrinsic motivation, self-confidence, and mood. Acceptability of WDYD was measured by survey questions; use was tracked via app data logs.

**Results:**

Analyses were based on data from 1767 participants; 720 in the intervention group and 1047 participants in the control group. Almost half of them were female (2276/4795, 47.5%), and most (3471/4595, 72.4%) participants were aged 18‐24 (median 19.40, IQR 2.92) years. The dropout rate was high, up to 96% (4603/4795) in the final 33rd week. No significant effect of WDYD was found on primary outcomes and mood, except for week 1 (excessive drinking: standardized *β*=−0.35, SE 0.15; 95% CI −0.64 to −0.05; binge drinking: standardized *β*=−0.36, SE 0.16; 95% CI −0.68 to −0.04; mood: standardized *β*=0.20, SE 0.06, 95% CI 0.08 to 0.32). Both groups reduced their alcohol consumption. Significant positive effects were found for intrinsic motivation and self-confidence up to 25 weeks (wk 25: standardized *β*=0.54, SE 0.24; 95% CI 0.06 to 1.02 for motivation; standardized *β*=0.72, SE 0.26; 95% CI 0.22 to 1.23 for self-confidence). Participants evaluated WDYD as acceptable and usable.

**Conclusions:**

WDYD did not significantly reduce excessive drinking compared to control, but improved motivation and self-confidence. High dropout rates highlight challenges in sustaining engagement in long-term mHealth interventions. Future research should explore strategies to enhance retention and optimize dynamic tailoring.

## Introduction

### Background

Excessive alcohol drinking among adolescents and young adults is a large public health concern in Western countries [1]; it is negatively associated with short-term and longer-term negative outcomes, with regard to health, academic performance, social relationships, and finishing school [1]. Specifically among adolescents and young adults, alcohol consumption occurs in groups and is strongly influenced by the group [2-4]. In the Netherlands, various studies show that adolescents and young adults engage in excessive drinking [5-7]. In accordance with the guidelines, excessive drinking is defined as heavy drinking (ie, drinking at least 8 [women] or 15 [men] alcoholic beverages per week) and/or binge drinking (ie, drinking at least 4 [women] or 6 [men] drinks at 1 occasion [8,9]). Preventing excessive drinking is important to improve the health of individuals and to reduce societal costs.

### Potential for mHealth Interventions

mHealth (mobile health) interventions can be a useful means to reach young people and to prevent them from drinking excessively [10]. mHealth interventions use mobile phones to deliver interventions and improve health, which are generally prevalent among young people (in the Netherlands, 99.1% uses a mobile phone for internet [11]). They are also widely accessible and can safeguard the users’ anonymity [12]. In addition, mHealth enables the effective combination of information and communication technology with behavior change methods [13]. Although mHealth can be useful, usable, and effective, currently available alcohol apps generally contain few behavior change strategies [14]. Empirical evidence is lacking for alcohol prevention mHealth interventions among adolescents and young adults specifically [10]. Research emphasized the need for tailoring [15], personalization [16], and intervening in real-life contexts [10] for this target group.

### WDYD Intervention

In this study, we aimed to evaluate an evidence-based 17-week dynamically tailored mHealth intervention, “What Do You Drink” (WDYD; Trial Registration: onderzoekmetmensen.nl Trial 28135 [17,18]). Dynamic tailored interventions, also called just-in-time adaptive interventions [19-21], are interventions where the information about the individual is used to determine when and how to intervene; that is, feedback and support are provided based on prior assessments of behavioral and psychological factors. In other words, to make these intervention-related decisions, not only static information (eg, baseline characteristics), but also time-varying information (eg, mood or response to intervention) is used [20]. Thus, dynamically tailored interventions are adapted over time to the dynamic changes of individuals, based on their varying needs. A meta-analytic review has shown that dynamic, tailored, just-in-time adaptive interventions are more effective than nondynamic, non–just-in-time adaptive interventions [19,21]. Moreover, dynamically tailored interventions should reduce dropout, a general problem of digital health interventions [10].

In addition, WDYD is based on behavior change techniques that have shown to be effective in changing health behavior and reducing alcohol consumption [22-24]. Examples of general methods used in WDYD are goal setting, action planning, behavioral feedback, and self-monitoring. In addition, based on daily assessments of behavioral (alcohol consumption) and psychological factors (motivation, self-confidence, and mood), users were provided with weekly tailored support based on a diversity of behavioral change techniques (eg, planning and problem solving, behavior substitution, modeling, and motivational interviewing techniques [18]).

### Objectives

The primary objective was to evaluate the effectiveness of WDYD in reducing excessive alcohol consumption among adolescents and young adults (≥16 y) at risk. Secondary objectives were to assess changes in determinants of drinking behavior (intrinsic motivation, self-confidence, and mood) and to examine the acceptability and use of WDYD.

### Hypotheses

We hypothesized that, compared to the control group (no intervention), participants receiving WDYD would (1) show greater reductions in excessive alcohol use, binge drinking, and weekly alcohol consumption (primary outcomes); (2) demonstrate positive changes in determinants of drinking behavior (secondary outcomes); and (3) evaluate the intervention as acceptable and usable, with measurable engagement reflected in app usage logs.

## Methods

### Ethical Considerations

#### Human Subjects Ethics Review

This study was approved by the Ethical Committee of the Faculty of Social Sciences of Radboud University Nijmegen (approval number ECSW2016-1403-390).

#### Informed Consent

Participants provided active informed consent within the app before study enrollment. Individuals younger than 16 years of age were excluded from participation; consent included confirmation that participants were aged 16 years or older (see Multimedia Appendix 1 for consent documents). Participants received a summary of this study’s purpose (ie, personalized education about alcohol intake), duration (ie, approximately 6 months), and procedures (including participation via an app, questionnaires, and ecological momentary assessments [EMAs], information about the study groups, and possible interventions). They were also informed that participation was voluntary and that they could withdraw at any time without providing a reason.

#### Privacy and Confidentiality

Participants were quasi-anonymous. Registration required an email address, which was linked to a unique participant code and stored separately. The Information and Communication Technology provider managing the app had access to both datasets; researchers only accessed deidentified data. Email addresses were shared with researchers solely for reward distribution and could not be linked to participant codes. Data protection measures were outlined in the terms of use and privacy statement included in the consent process (see Multimedia Appendix 1). No cookies or third-party tracking tools were used to prevent multiple registrations, and no additional identity verification measures were implemented.

#### Compensation

Participants were compensated for their participation. Those who completed both follow-up surveys received a minimum of €25 (US $29.15; €12.50 [US $14.63] per completed survey) and up to €80 (US $93.92) in bol.com vouchers, depending on overall study completion rates. Compensation procedures were communicated during the consent process (see Multimedia Appendix 1).

### Design

This study was a 2-arm, parallel-group, online-only randomized controlled trial conducted in the Netherlands between September 2018 and October 2019. The target population was adolescents and young adults aged ≥16 years who drank excessively. Participants could enroll between September 2018 until the end of January 2019 by downloading the stand-alone WDYD app from the iOS App Store (Apple Inc)or Google Play Store (Google LLC). Within the WDYD app, participants were randomly assigned (1:1) to the experimental group (ie, WDYD intervention) or control group (no intervention), using a preprogrammed algorithm embedded in WDYD (see reference for the language code [25]). During consent, participants were informed that 2 study groups existed. Individual allocation was unknown at the time of consent, because randomization occurred after participants provided consent. After randomization, no allocation disclosure was provided. However, participants could infer their group assignment from the presence (intervention) or absence (control) of tailored sessions. The trial lasted 33 weeks and consisted of an online survey at baseline, followed by additional online surveys at 9 and 33 weeks. To account for the fluctuating nature of alcohol drinking and contextual influences [26,27], participants were also asked to report their alcohol intake each day using EMA over 7 weeks (wk 1, 7, 13, 19, 25, 31, and 33), each consisting of 7 consecutive daily assessments. Surveys and EMAs were administered via the WDYD app, with push notifications as reminders.

The trial was conducted with version 2.0.0 of the WDYD app (d.d. August 27, 2018). Two updates were made to WDYD during the trial: (1) small textual changes (September 12, 2018), and (2) a bug fix and removal of text about participation rewards at the end of the enrollment period (January 30, 2019).

### Participants

The eligibility criteria were based on the Dutch Health Council guidelines for excessive drinking [9] and being 16 years or older. Adolescents (aged 16‐18 years) indicating any amount of routine alcohol consumption were considered excessive drinkers. For adults, excessive drinking was defined as:

- Men: drinking more than 2 drinks per day (heavy drinking) or 6 or more glasses in 1 day (binge drinking).
- Women: drinking more than 1 glass per day (heavy drinking) or 4 or more glasses in 1 day (binge drinking).

Participants also had to be 16 years of age or older at the time they signed up.

Initially, WDYD was developed as an intervention with a primary focus on students in secondary vocational education who drank excessively (aged 16 to 24 years). The intervention was developed in cocreation with these students using a user-centered approach [18]. Due to low enrollment in a prior pilot (n=253; 19.3% of the 1310 participants needed), recruitment was expanded to all Dutch adolescents and young adults who drank excessively.

Multiple recruitment channels were used:

1. Students in secondary vocational education were recruited via an online lifestyle monitor (“TestYourLifestyle”; *Testjeleefstijl* in Dutch) specifically designed for schools and students in secondary vocational education [28]. The monitor is used annually by multiple schools as part of their health-education curriculum. The monitor consists of various lifestyle modules (eg, physical activity, nutrition behavior, sexual behavior, or smoking), including alcohol consumption. Within the monitor, students can create a user account, receive module-specific questions followed by brief tailored normative feedback (ie, comparison of their behavior to the Dutch guidelines), behavior-related risk information, and information about websites or programs related to the lifestyle topic. To recruit students in secondary vocational education for this study, students who drank alcohol excessively according to the monitor received an invitation to participate with a link to further information about this study and the possibility to download the WDYD app to subscribe and provide informed consent for participation. During the recruitment period (September 2018 to January 2019), the alcohol module of the lifestyle monitor was completed by 12,004 students. Of these students, 55.1% (6619/12004) exceeded the heavy-drinking guideline, and 52.4% (6290/12004) reported binge drinking, making them eligible for participation in WDYD. The lifestyle monitor is implemented at the school level, but detailed information on the number of participating schools and class-level participation rates was not available.
2. Students in secondary vocational education were also recruited via teachers who implemented an educational program on alcohol consumption. The Secondary Vocational Education Council distributed an email invitation and published a news item on its website aimed at teachers responsible for citizenship education, a compulsory subject in secondary vocational education. Interested teachers could sign up via email to deliver the educational program in their classes. Unfortunately, the number of teachers and schools that received the initial invitation is not known. After completing the educational program, teachers were asked to invite their students to participate in the WDYD study by providing access to this study’s information page and the WDYD app.
3. Via a social media campaign (Instagram or Facebook) targeting people aged 16‐24 years in the Netherlands.
4. Via news items and flyers by the Addiction Prevention and Care Institute [29].
5. As the eligibility of participants who drank excessively could not be established via the recruitment channels before enrollment, we intentionally oversampled and subsequently excluded individuals who did not meet the inclusion criteria.

### Intervention

WDYD was developed systematically using the Intervention Mapping planning protocol [30] and an iterative, user-centered design process [31]. The development is described elsewhere [18]. WDYD (version 2.0.0) delivered a 17-week dynamically tailored mHealth program.

Regarding content and access to the intervention, WDYD offered a total of 39 different exercises and 22 videos targeting five key goals: (1) motivation, (2) self-confidence, (3) mood, (4) planning, and (5) relapse prevention, using evidence-based techniques (eg, motivational interviewing [32], behavioral modeling [33], goal setting [34], self-monitoring [35], implementation intentions [36], cognitive-behavior therapy [37], and provision of rewards [38]) and engagement strategies [22,23,39]. In addition, participants could monitor their goal progress in WDYD, in which historic daily alcohol intake was registered, as well as whether they had reached their self-set goal. Screenshots of the intervention are shown in Multimedia Appendix 2.

**Figure 1.**
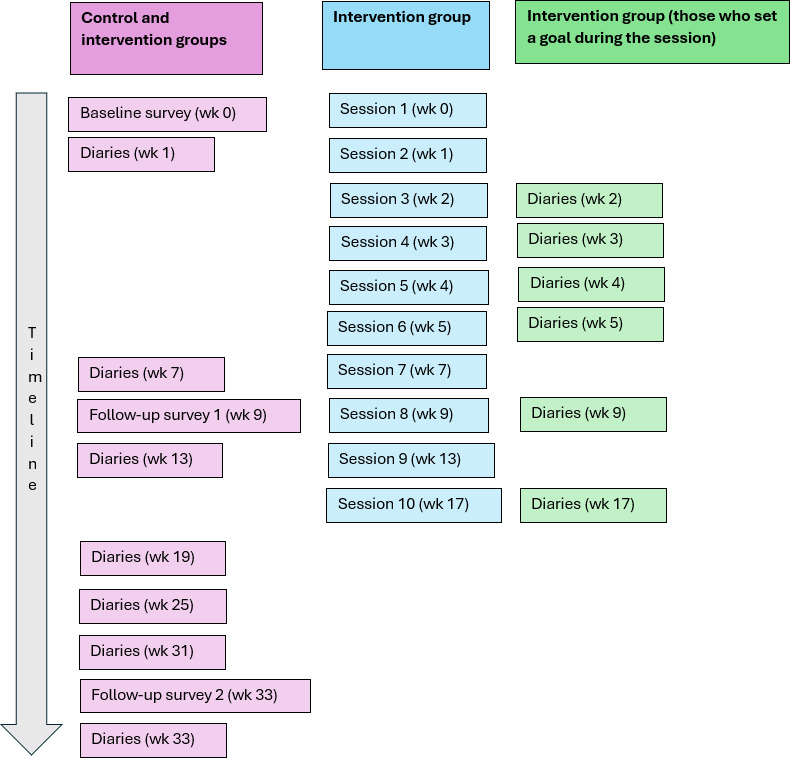
Flow of the WDYD intervention content and timing. WDYD: What Do You Drink.

Exercises were tailored based on EMA data. Participants who set a goal were asked to daily monitor their alcohol drinking behavior, mood, motivation, and self-confidence. They received push notifications to remind them to provide a daily report. The intervention group received tailored weekly sessions and reminders; this started with 6 weekly sessions (wk 0‐5), after which the frequency of sessions reduced to 2 biweekly sessions (wk 7 and 9), and 2 monthly sessions (wk 13 and 17). See Figure 1 for an overview of the intervention content and timing.

### Control Group

The control group completed surveys (baseline, wk 9, and wk 33) and EMAs only (7 daily assessments during wk 1, 7, 13, 19, 25, 31, and 33), without receiving any feedback or support. They reported their day-to-day alcohol consumption and mood in the EMAs. Motivation and self-confidence to drink less were only asked on the first day of each EMA week.

The control group was also invited to fill in the online baseline survey, and the first and second online follow-up surveys (wk 9 and 33).

### Procedure

Participants were invited to participate in this study via various recruitment strategies (see Participants section) and invited to download the WDYD app; access was open and free of charge. Participants who downloaded the app could sign up and provide informed consent in the app. After consent, participants completed a baseline survey measuring sociodemographic variables, and primary and secondary outcomes. Participants in the experimental group received tailored feedback sessions. Invitations for follow-up surveys at weeks 9 and 33, with an estimated completion time of, respectively, 10 and 5 minutes, were sent via push notifications at 7 PM, followed by up to 3 reminders (ie, at d 1, 4, and 7). At the start of a survey, participants were informed about the expected duration of each survey. Participants were given 30 days to complete the follow-up surveys.

### Outcome Variables

Outcomes were measured via closed online surveys at baseline, 9, and 33 weeks, and via EMAs consisting of brief daily assessments for 7 consecutive days (wk 1, 7, 13, 19, 25, 31, and 33). All measurements were administered within the WDYD app and pretested for usability and technical functionality.

### Primary Outcomes

Weekly alcohol consumption was measured as the number of standard drinks per week, assessed at baseline using daily intake questions [40,41] and during EMA bursts using the question: “How many glasses of alcoholic drinks did you drink yesterday?” (range: 0‐100) [40]. Weekly alcohol intake was calculated based on a minimum of 4 EMAs in a given week.

Binge drinking was defined as drinking more than 3 (women) or more than 5 (men) standard alcohol units on at least 1 day of the week; the day with the highest reported number of glasses consumed was used to determine whether binge drinking occurred.

Excessive drinking was defined as binge drinking (ie, drinking 4 [women] or 6 [men] or more glasses in 1 day) and/or heavy drinking (ie, drinking any amount of alcohol when under the legal drinking age of 18 years, and for adults to drink a maximum of 1 [women] or 2 [men] glasses of standard alcohol units on an average day [9]). Heavy drinking was determined by comparing the extrapolated number of glasses of alcoholic drinks in a week (more than 7 glasses for women, and more than 14 glasses for men).

### Secondary Outcomes

Intrinsic motivation was measured by the question: “How important is it for you to drink less alcoholic drinks” (0=not at all important to 10=very important) [32].

Self-confidence was assessed with the question: “How confident are you that you can drink less alcoholic drinks” (0=not at all confident to 10=very confident) [41,42].

Mood was assessed with the question: “How do you feel?” (0=very bad to 10=very good [41,43,44]. These were measured at baseline (except for mood), week 1 (except for motivation and confidence), and weeks 7, 13, 19, 25, 31, and 33. For mood, the weekly average was calculated if participants filled out a minimum of 4 daily assessments.

Motivation and self-confidence were measured on the first day of each EMA week. Mood was measured daily in an EMA week, and an average weekly mood index was created based on a minimum of 4 EMA entries.

### Sociodemographic Variables

Sociodemographic variables were measured at baseline. These variables were age, gender, migrant background, ongoing educational level, and highest completed educational level. Both education variables were rescored to low (less than secondary or vocational education), intermediate (secondary through preuniversity education) or high (professional or university education). Specifically for students in secondary vocational education, we assessed their qualification level (1-4), school year (1-4), and pathway (practical, vocational training, or combination).

### Program Evaluation and Use

#### Program Evaluation

Participants in the intervention group were asked to evaluate WDYD in the first follow-up survey (week 9).

WDYD usability was assessed by 5 items, for example, “How do you perceive the user-friendliness of WDYD?” on a 10-point scale (eg, 1=not at all user-friendly to 10=very user-friendly),

Information quality was also assessed by 5 items (eg, “How do you perceive the credibility of the information in WDYD?” with 10-point scales (eg, 1=not at all credible to 10=very credible).

Personalization was assessed by 8 items (eg, “WDYD took into account my personal preferences,” scales 1=totally agree to 5=totally disagree).

Finally, participants were asked to grade the overall acceptability of WDYD on a scale from 1 (very bad) to 10 (excellent) [45].

#### Program Use

For participants in the intervention group, use of WDYD was logged in the app. Session completion was calculated as the average number of sessions completed across all participants in the intervention group.

Exercises completed were calculated by dividing the number of completed exercises within a given category by the total number of exercises participants had chosen.

### Power Calculation

The sample size was based on a priori power calculation for detecting a reduction in excessive drinking in the intervention group compared to the control group. We anticipated a small effect size (Cohen *d*=0.20) based on a previous study of an earlier version of WDYD [27] and a just-in-time adaptive alcohol intervention [39]. In the previous study, the EMA procedure, including the rewarding of participants, resulted in a 20% dropout [27]. When anticipating a conservative dropout rate of 40% [46], a small effect size (*d*=0.20), a power of 0.80, and a 2-sided *α* of .05, a total of 655 participants were required per group at baseline.

### Statistical Analyses

Data were analyzed using R (R Foundation) [47]. Logistic regression analyses were also used to analyze the dropout probability at the start (ie, within the first week), and throughout this study (ie, within 33 wk), with primary, secondary, and sociodemographic variables at baseline as predictors. Descriptive statistics (%, n, mean, SD, median, or IQR) were used to analyze baseline characteristics of the sample (ie, sociodemographic and outcome variables) and to evaluate usability and acceptability of WDYD. A logistic regression analysis was used to evaluate the randomization by analyzing the probability of intervention group assignment using the primary, secondary, and sociodemographic variables observed at baseline. Baseline logistic regression analyses were used to examine differences between recruitment strategies in drinking behavior at baseline.

Effects of WDYD on primary and secondary outcomes were analyzed using multilevel generalized linear regression models using the R package *lme4* [48]. A multilevel logistic regression model was used for dichotomous outcomes (ie, excessive drinking and binge drinking), a multilevel negative binomial regression model for count outcomes (ie, number of glasses), and a linear multilevel regression model for continuous outcomes (ie, mood, motivation, and self-confidence). In these models, time (categorical: a dummy for every diary week with baseline as reference category), group (intervention vs control), and the interaction between time and group were used as predictors. In addition, a random intercept was included in each model to account for within-person clustering over time.

Missing values were imputed using multiple imputation with multilevel predictive mean matching using 100 imputations and 25 iterations, using the R package *mice* [49]. The imputation model contained the primary and secondary outcomes (alcohol consumption, mood, intrinsic motivation, and self-confidence) from the daily assessment data, group, enrolled in secondary vocational education, time, number of sessions attended (0‐10), gender, and intention to reduce excessive drinking at baseline and week 9. We performed additional complete case analyses on both primary and secondary outcomes to check for differences between analyses based on multiple imputation and those based on complete cases only.

## Results

### Response Rates and Dropout

**Figure 2.**
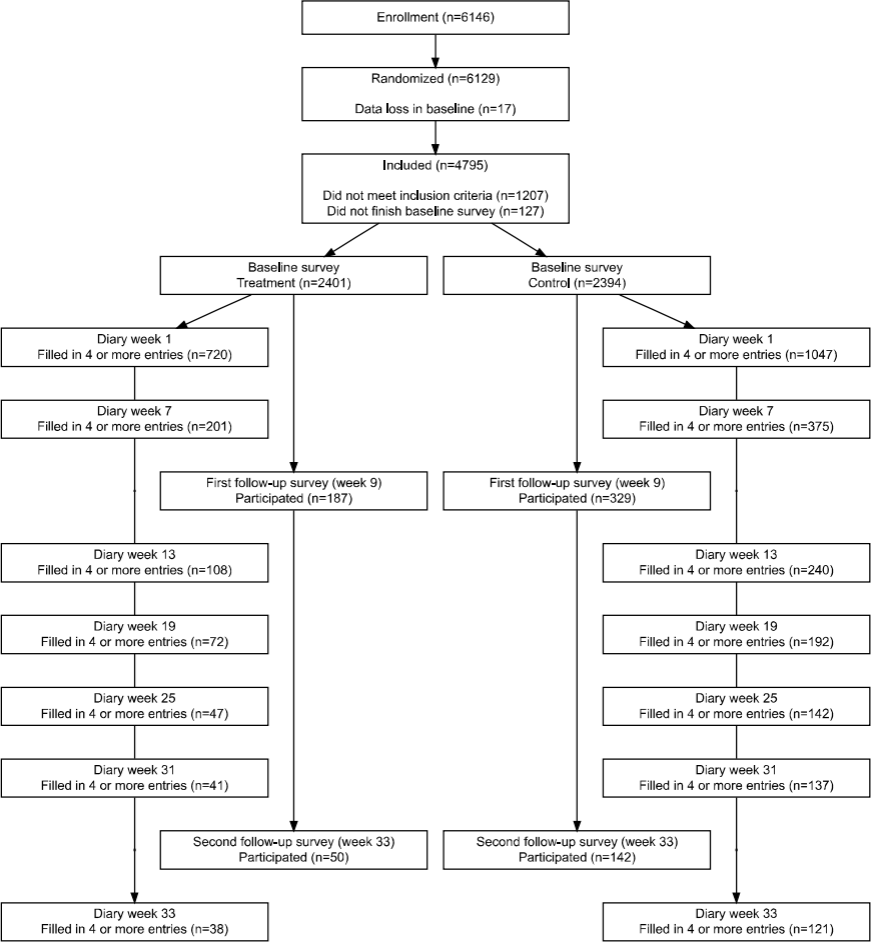
Flow of participants in the WDYD study. WDYD: What Do You Drink.

The flow of participants through this study is shown in Figure 2. A total of 6146 people downloaded the WDYD app between September 1, 2018, and January 31, 2019, and agreed to participate in this study. Most participants (4168/6146, 68.1%) indicated having been recruited via Facebook (Meta) or Instagram (Meta). Other recruitment sources included referrals from friends or relatives (637/6146, 10.4%), nonsocial media websites (465/6146, 7.6%), the online lifestyle monitor (327/6146, 5.3%), and through their teacher (272/6146, 4.4%). Finally, 4.6% (280/6146) participants found this study through another channel.

We were unable to determine the group allocation of 17 participants due to data loss of group allocation at baseline. The remaining 6129 participants were randomized into the intervention (n=3088) or control group (n=3041). After filling in the baseline survey, 1334 participants did not meet the inclusion criteria and were excluded from further analysis. Of the participants who met the inclusion criteria, 36.9% (1767/4795) completed the first week of daily assessments, 10.8% (516/4795) completed the first follow-up survey (wk 9), and 4% (192/4795) completed the second follow-up survey (wk 33). Participants who were enrolled in secondary vocational education (exp(β)=2.034, *P*<.001) and had a higher peak daily alcohol consumption (exp(β)=1.040, *P*<.001) were more likely to drop out within the first week of this study. Over the entire 33 weeks of this study, participants assigned to the intervention group (exp(β)=2.96, *P*<.001), enrolled in secondary vocational education (exp(β)=3.59, *P*<.001), and had a higher mean weekly alcohol consumption (exp(β)=1.034, *P*<.001) were more likely to drop out.

Participants were excluded from all analyses when they did not meet the inclusion criteria (ie, excessive drinking and ≥16 y of age; see Participants section; n=1207) or if they did not complete the baseline questionnaire (n=127). The number of participants that dropped out of this study was large, especially in the first diary week (n=3028). We excluded participants who dropped out within the first diary week from the main analysis, as not enough information was observed to allow for imputation. In addition, the number of participants who completed the measurements during weeks 31 and 33 was insufficient for reliable imputation. As a result, our imputation model was unable to produce stable imputed outcomes. For this reason, we restricted our analysis to data collected up to week 25.

The final sample for the main analyses consisted of 1767 participants (n_intervention_=720, n_control_=1047). Missing values of these participants were imputed for analyses. Trace plots showed that the imputed datasets had converged, and density and scatterplots between observed and imputed data showed plausible imputed values.

### Baseline Characteristics

Baseline characteristics of participants are depicted in Table 1. There were no significant differences between participants in the intervention and control groups regarding sociodemographic characteristics, primary and secondary outcomes (data not shown). Small differences were found in drinking behavior by recruitment strategy. Participants reporting higher weekly alcohol consumption were more likely to be recruited via social media (exp(β)=1.01, *P*<.001) and less likely via teachers (exp(β)=0.95, *P*<.001) or the lifestyle monitor (exp(β)=0.92, *P*<.001). No other significant baseline differences were observed in drinking behavior, and no interaction effects of recruitment strategy with group assignment were found. Slightly more than half of the participants were male (2519/4795, 52.5%), 72.4% (3471/4795) participants were aged 18 to 24 years, most of them attended or had completed a higher educational level (2673/4795, 56.3%), and 34.6% (1659/4795) of them attended secondary vocational education.

**Table 1.** Baseline characteristics of WDYD^a^ study participants.

	Control group (n=2394)	Intervention group (n=2401)	Total (n=4795)
Sociodemographic background			
Sex, n (%)			
Male	1248 (52.1)	1271 (52.9)	2519 (52.5)
Female	1146 (47.9)	1130 (47.1)	2276 (47.5)
Age (years), median (IQR)	19.36 (2.95)	19.42 (2.90)	19.40 (2.92)
<18, n (%)	574 (24)	563 (23.5)	1137 (23.7)
18‐24, n (%)	1732 (72.4)	1739 (72.4)	3471 (72.4)
≥24, n (%)	88 (3.7)	99 (4.12)	187 (3.9)
Education, n (%)			
Low	158 (6.67)	126 (5.3)	284 (6)
Medium	880 (37.2)	909 (38.2)	1789 (37.7)
High	1330 (56.2)	1343 (56.5)	2673 (56.3)
Student in secondary vocational education, n (%)	824 (34.4)	835 (34.8)	1659 (34.6)
Secondary vocational education school year, n (%)			
1	322 (39.1)	339 (40.6)	661 (39.8)
2	273 (33.1)	275 (32.9)	548 (33)
3	175 (21.2)	165 (19.8)	340 (20.5)
4	54 (6.55)	56 (6.71)	110 (6.6)
Secondary vocational education qualification level, n (%)			
1	8 (0.97)	3 (0.44)	11 (0.66)
2	83 (10.07)	82 (9.82)	165 (10)
3	169 (20.5)	184 (22)	353 (21.3)
4	564 (68.5)	566 (67.8)	1130 (68.1)
Secondary vocational education pathway, n (%)			
Practical training	229 (27.8)	225 (27)	454 (27.4)
Vocational training	571 (69.3)	580 (69.5)	1151 (69.38)
Combination	24 (2.91)	30 (3.59)	54 (3.25)
Migrant background, n (%)			
No	2192 (91.6)	2189 (91.2)	1615 (91.4)
Yes	202 (8.44)	212 (8.83)	414 (8.63)
Primary outcomes			
Excessive drinking, n (%)^b^			
No	0 (0)	0 (0)	0 (0)
Yes	2394 (100)	2401 (100)	4795 (100)
Number of glasses of alcoholic drinks consumed in a week, median (IQR)	16.00 (16.00)	16.00 (15.00)	16.00 (16.00)
Binge drinking, n (%)			
No	655 (27.4)	680 (28.3)	1335 (27.8)
Yes	1739 (72.6)	1721 (71.7)	3460 (72.2)
Binge drinking frequency per week, mean (SD)	1.67 (1.11)	1.66 (1.12)	1.67 (1.12)
Peak daily consumption, median (IQR)	6.00 (3.00)	6.00 (3.00)	6.00 (3.00)
Secondary outcomes			
Motivation to drink less (0‐10), median (IQR)	3.00 (5.00)	3.00 (5.00)	3.00 (5.00)
Self-confidence to drink less (0‐10), mean (SD)	4.52 (2.86)	4.51 (2.94)	4.52 (2.90)

a WDYD: What Do You Drink.

b This variable was the inclusion criterion.

### Intervention Effects

**Figure 3.**
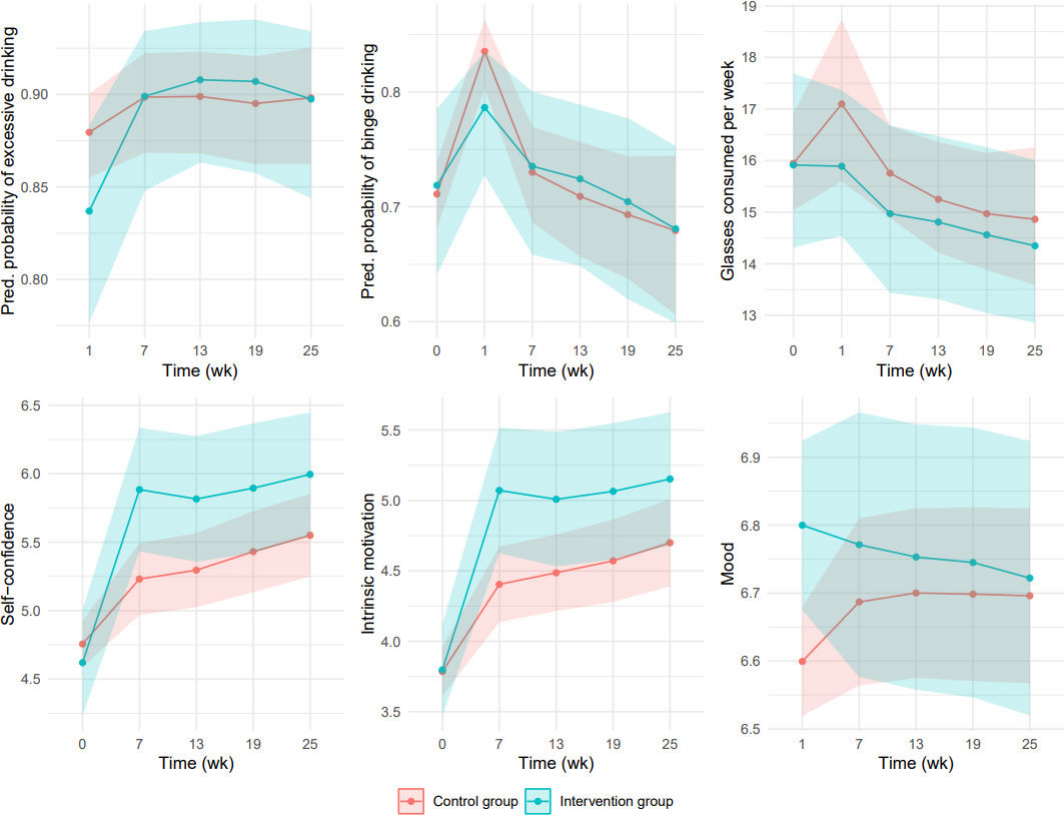
Predicted outcomes of WDYD for the control and intervention groups according to the multilevel model based on multiple imputation. Bands indicate the 95% pointwise CIs for the control and intervention groups. The CI for the intervention group has the coefficient of time and group fixed at its mean predictor values. Pred: predicted; WDYD: What Do You Drink.

Effects of WDYD on primary and secondary outcomes based on multiple imputation are depicted in Table 2 and Figure 3. In both groups, binge drinking and mean weekly alcohol consumption significantly increased from baseline to week 1. After week 1, both groups decreased their alcohol consumption, but this did not significantly differ from baseline. There were no significant differences between groups on the primary outcomes at different time points (wk 1, 7, 13, 19, and 25), with the exception of a positive effect in favor of the intervention group for binge drinking and excessive drinking at week 1. Participants in the intervention group were less likely to drink excessively or binge compared to the control group in week 1. At later time points (wk 7, 13, 19, and 25), we do not have enough evidence to suggest that both groups differ. Figure 3 shows that there is a large amount of uncertainty in our model parameters, especially at later points in time when less data was available.

With regard to the secondary outcomes, motivation and self-confidence increased in both groups from weeks 7 to 25. Positive effects of WDYD were found for motivation and self-confidence at weeks 7, 13, 19, and 25; participants in the intervention group had significantly increased their motivation and self-confidence to consume less alcohol than those in the control group. As for mood, participants in the intervention group reported a more positive outcome in week 1 than participants in the control group; this difference was small but significant. After week 1, the difference between groups was no longer significant. Week 3 shows similar uncertainty in estimation for motivation, self-confidence, and mood.

We did conduct additional moderation analyses and found no evidence of educational attainment moderating the treatment effect (data not shown).

Effects on the primary and secondary outcomes were additionally evaluated using complete case analysis; results (data not shown) were similar to those based on multiple imputation of missing values.

**Table 2.** Effects of WDYD^a^ on primary and secondary outcomes based on multiple imputation with sample statistics based on unimputed data.

Time	Sample statistics						Model coefficients (group×time)		Effect sizes
	N_control_	n (%)	Mean (SD)	N_intervention_	n (%)	Mean (SD)	B_intervention_ (SE)	95% CI	OR^b^/IRR^c^/ StdB^d^
Excessive drinking^e^									
Baseline	N/A^f^	N/A	N/A	N/A	N/A	N/A	N/A	N/A	N/A
Week 1	1047	877 (83.7)	N/A^g^	720	568 (78.9)	N/A^g^	−0.35 (0.15)	−0.64 to −0.05^e^	0.70^b^
Week 7	297	220 (74.1)	N/A^g^	198	146 (73.7)	N/A^g^	0.34 (0.24)	−0.13 to 0.80	0.99^b^
Week 13	192	135 (70.3)	N/A^g^	106	76 (71.7)	N/A^g^	0.43 (0.24)	−0.05 to 0.90	1.08^b^
Week 19	157	111 (70.7)	N/A^g^	70	51 (72.86)	N/A^g^	0.45 (0.26)	−0.06 to 0.96	1.11^b^
Week 25	106	81 (76.42)	N/A^g^	47	32 (68.09)	N/A^g^	0.32 (0.26)	−0.20 to 0.83	0.97^b^
Binge drinking^e^									
Baseline	1047	734 (70.1)	N/A^g^	720	510 (70.8)	N/A^g^	0.04 (0.11)	−0.18 to 0.25	1.04^b^
Week 1	1047	863 (82.4)	N/A^g^	720	558 (77.5)	N/A^g^	−0.36 (0.16)	−0.68 to −0.04^e^	0.73^b^
Week 7	297	214 (72.1)	N/A^g^	198	144 (72.7)	N/A^g^	−0.01 (0.19)	−0.38 to 0.35	1.03^b^
Week 13	192	133 (69.3)	N/A^g^	106	76 (71.7)	N/A^g^	0.00 (0.19)	−0.38 to 0.38	1.04^b^
Week 19	157	110 (70.1)	N/A^g^	70	49 (70)	N/A^g^	−0.02 (0.19)	−0.39 to 0.36	1.02^b^
Week 25	106	78 (73.58)	N/A^g^	47	32 (68.09)	N/A^g^	−0.02 (0.20)	−0.42 to 0.38	1.01^b^
Number of glasses of alcoholic drinks per week^e^									
Baseline	1047	N/A^g^	16 (15)	720	N/A^g^	17 (16)	0.00 (0.05)	−0.09 to 0.09	1.00^c^
Week 1	1047	N/A^g^	17 (22.5)	720	N/A^g^	16 (22)	−0.07 (0.05)	−0.16 to 0.02	0.93^c^
Week 7	297	N/A^g^	15	198	N/A^g^	12.5 (20)	−0.05 (0.05)	−0.16 to 0.06	0.95^c^
Week 13	192	N/A^g^	11.5 (24)	106	N/A^g^	13 (19.75)	−0.04 (0.05)	−0.14 to 0.07	0.96^c^
Week 19	157	N/A^g^	11 (22)	70	N/A^g^	10 (15)	−0.03 (0.05)	−0.13 to 0.08	0.97^c^
Week 25	106	N/A^g^	13.5 (19.5)	47	N/A^g^	10 (16.5)	−0.03 (0.06)	−0.15 to 0.08	0.96^c^
Mood^e^									
Baseline^h^	N/A	N/A	N/A	N/A	N/A	N/A	N/A	N/A	N/A
Week 1	973	N/A^g^	6.59 (1.27)	691	N/A^g^	6.81 (1.34)	0.20 (0.06)	0.08 to 0.32^e^	0.19^d^
Week 7	309	N/A^g^	6.63 (1.11)	193	N/A^g^	6.94 (1.36)	−0.12 (0.10)	−0.32 to 0.08	0.08^d^
Week 13	196	N/A^g^	6.68 (1.11)	105	N/A^g^	6.97 (1.27)	−0.15 (0.10)	−0.35 to 0.05	0.05^d^
Week 19	158	N/A^g^	6.62 (1.22)	71	N/A^g^	7.01 (1.37)	−0.15 (0.11)	−0.36 to 0.06	0.04^d^
Week 25	112	N/A^g^	6.65 (1.14)	46	N/A^g^	6.80 (1.44)	−0.18 (0.1)	−0.38 to 0.02	0.02^d^
Self-confidence^i^									
Baseline	1046	N/A^g^	4.76 (2.80)	719	N/A^g^	4.62 (2.90)	−0.13 (0.15)	−0.42 to 0.15	-0.05^d^
Week 1	N/A^j^	N/A^j^	N/A^j^	324	N/A^g^	7.65 (2.96)	N/A^j^	N/A^j^	N/A^j^
Week 7	164	N/A^g^	5.23 (2.86)	185	N/A^g^	6.95 (2.93)	0.91 (0.26)	0.40 to 1.41^e^	0.22^d^
Week 13	111	N/A^g^	5.18 (2.90)	91	N/A^g^	7.02 (2.96)	0.75 (0.26)	0.25 to 1.25^e^	0.18^d^
Week 19	75	N/A^g^	5.04 (3.00)	73	N/A^g^	6.88 (3.20)	0.72 (0.27)	0.18 to 1.25^e^	0.16^d^
Week 25	50	N/A^g^	5.02 (3.06)	44	N/A^g^	7.32 (3.00)	0.72 (0.26)	0.22 to 1.23^e^	0.15^d^
Motivation^i^									
Baseline	1046	N/A^g^	3.78 (2.74)	718	N/A^g^	3.79 (2.85)	0.01 (0.14)	−0.27 to 0.3	0.00^d^
Week 1	N/A^j^	N/A^j^	N/A^j^	324	N/A^g^	6.63 (2.93)	N/A^j^	N/A^j^	N/A^j^
Week 7	166	N/A^g^	4.40 (2.74)	185	N/A^g^	6.38 (2.87)	0.81 (0.25)	0.31 to 1.31^e^	0.22^d^
Week 13	114	N/A^g^	4.51 (3.02)	92	N/A^g^	6.66 (3.00)	0.60 (0.24)	0.13 to 1.07^e^	0.18^d^
Week 19	76	N/A^g^	4.30 (2.70)	73	N/A^g^	6.58 (2.89)	0.56 (0.24)	0.08 to 1.04^e^	0.17^d^
Week 25	50	N/A^g^	3.96 (2.94)	44	N/A^g^	6.91 (2.84)	0.54 (0.24)	0.06 to 1.02^e^	0.15^d^

a WDYD: What Do You Drink.

b OR: odds ratio for excessive drinking and binge drinking.

c IRR: incidence rate ratio for the number of glasses per week.

d StdB: standardized *β* for mood, self-confidence, and motivation.

e Significant difference between intervention and control condition (*P*<.05).

f N/A: not applicable.

g Mean (SD) was not applicable for the dichotomous outcomes excessive drinking and binge drinking, and n (%) was not applicable for the continuous outcomes of number of glasses of alcoholic drinks per week, mood, self-confidence, and motivation.

h Baseline was not included in the analyses because all participants engaged in excessive drinking at baseline.

i Mood was not measured at baseline, and had week 1 as the reference category for time.

j Self-confidence and motivation were not measured at week 1 in the control group.

### Program Evaluation and Use

The program evaluation and use of WDYD among intervention participants are depicted in Table 3. Participants were very positive about WDYD; they evaluated the overall acceptability of WDYD with a mean grade of 7.17 (SD 1.07; 0=very bad to 10=excellent). The usability of WDYD was evaluated as good (with mean grades of 6.78, SD 1.73, or higher at a 10-point scale). The informativity and usefulness of WDYD were evaluated as satisfactory (means 5.69, SD 2.04, and 5.46, SD 2.05, respectively), and the credibility and understandability as good (means 7.25, SD 1.41, and 7.97, SD 1.44, respectively). The amount of information was perceived as adequate (mean 5.78, SD on a scale from 0=too little to 10=too much). In addition, participants positively evaluated the design principles of WDYD (eg, acceptation and freedom of choice).

The average number of sessions followed reflects the high dropout rate (mean 1.67, SD 2.12). Within the intervention sessions, most participants received a motivation exercise (1475/4298, 34.3%) or a planning exercise (1465/4298, 34.1%).

**Table 3.** Program evaluation and use of WDYD^a^. A higher value is always better unless indicated otherwise.

	Values
Program evaluation (n=187)	
Overall acceptability (0=very bad to 10=excellent), mean (SD)	7.17 (1.07)
Usability (0=not at all or very bad to 10=excellent), mean (SD)	
User-friendliness	7.67 (1.59)
Fastness	8.51 (1.46)
Navigation ease	7.99 (1.55)
Design	7.43 (1.48)
Fun of use	6.78 (1.73)
Information in WDYD (0=not at all/very bad to 10=excellent), mean (SD)	
Informative	5.69 (2.04)
Usefulness	5.46 (2.05)
Credibility	7.25 (1.41)
Understandability	7.97 (1.44)
Amount of information^b^	5.78 (1.77)
Design principles (1=totally disagree to 5=totally agree), mean (SD)	
Free to choose my alcohol consumption	4.30 (0.75)
Learned to set goals to reduce drinking	3.33 (0.95)
Assumed I knew what was best for myself	3.74 (0.91)
Discussed topics I find important	3.63 (0.87)
Addressed in a pleasant way	4.17 (0.66)
Accounted for my personal preferences	3.71 (0.90)
Accepted me as I am	4.01 (0.80)
Program use (n=2401)	
Average number of sessions followed, mean (SD)	1.67 (2.12)
Exercises received (n and % of exercises)	
Mood exercise	807 (18.8)
Motivation exercise	1475 (34.3)
Self-confidence exercise	530 (12.3)
Planning exercise	1465 (34.1)
Relapse exercise	0.49 (21)

a WDYD: What Do You Drink.

b 0=too little, 10=too much, with 5 as the center.

## Discussion

### Principal Results

This study evaluated the effect, use, and acceptability of a dynamically tailored mobile intervention, WDYD, to reduce excessive alcohol drinking among at-risk adolescents and young adults. Regarding the primary outcomes, we found no support for our hypothesis that WDYD was more effective than the control group in reducing alcohol consumption. No effect of WDYD was found on mean weekly alcohol consumption. Positive effects of WDYD were found only in the first week for excessive drinking. In addition, there was an increase in binge drinking in the first week in both groups, but this increase was significantly smaller in the intervention group. After this first week, alcohol consumption seemed to decrease over time in both groups, but differences between groups were nonsignificant at all time points. The increase in binge drinking in the first week was possibly due to the measurement difference between baseline and the following weeks. At baseline, alcohol consumption was based on a participant’s self-reported alcohol intake during a typical week, whereas at the follow-up measurements, participants were asked to report their consumption of the day before (1-day recall) for 7 consecutive days. A 1-day recall leads to more accurate estimates than a 7-day recall because it is less susceptible to recall bias [50], and this difference is even more pronounced with binge drinking [40].

The reduction in alcohol consumption over time in both groups in this study is in contrast with the study about a previous web-based version of WDYD [27], which showed that both groups increased their alcohol consumption over time, with the intervention group showing a significantly lower increase than the control group. Differences between this study and the previous study may partly explain the variation of observed effects. The previous study used a web-based brief, single-session tailored feedback intervention. Effects were measured during 30 weekly 7-day recall EMAs over 6 months among a highly educated sample of young adults (ie, students in higher professional education or university) recruited via flyers and included when they were ready to change their alcohol consumption. In this study, the reduction of alcohol consumption in both groups over time, as well as the absence of a significant intervention effect on alcohol consumption, could be explained by the use of daily 1-day recall EMAs; the study’s participants, including those in the control group, self-monitored their alcohol drinking behavior daily for 7 weeks during this study. Self-monitoring is shown to be an important behavior change technique in predicting positive outcomes regarding alcohol reduction research [14,23]. Therefore, it is highly likely that the difference in outcomes between groups would have been greater if the control group had engaged in less frequent self-monitoring. Moreover, a difference between the studies lies in the inclusion criteria. First, the 2 studies targeted different populations. The previous study focused on highly educated young adults (ie, students in higher professional education and university), whereas this study primarily targeted adolescents and young adults from secondary vocational education and expanded recruitment to a broader group of Dutch adolescents and young adults. These populations differ in educational level, lifestyle context, and potentially in digital literacy, which may influence engagement and responsiveness to tailored feedback. Second, the previous study included participants who were already ready to change their alcohol consumption [27], a factor known to predict intervention responsiveness and behavior change outcomes [51,52]. In contrast, this study did not preselect participants based on their readiness to change, resulting in a more heterogeneous sample in terms of readiness. This combination of a different target group and broader variation in readiness may have reduced intervention effects compared to the earlier trial.

This study shows that there was a large variation between individuals in alcohol use and its determinants over time. This is in line with earlier research [27,53] and supports the dynamically tailored nature of WDYD. At the same time, it emphasizes a need for additional data analyses besides the evaluation of group-level effects. Specifically, an analysis method that accounts for differences between and within individuals over time to learn why and for whom the intervention is effective and to improve future dynamically tailored interventions [54].

An important flaw of this study was the high dropout rates, with 63.1% (3028/4795) within the first week, rising to 96% (4603/4795) at the final week 33, even though we applied several strategies to prevent dropout (eg, rewards, therapeutic alliance, and dynamic tailoring [10,21]). Dropout was higher among students in secondary vocational education and those who consumed more glasses of alcohol weekly. High dropout is a common problem in mHealth interventions, especially in the first 2 weeks of the study and among those most at-risk, and this needs attention (eg, [10,55]). EMA is a promising means to provide dynamic tailoring; however, it also places a cognitive burden upon participants and presents difficulties in keeping participants engaged [21]. Although sensor technology may be a promising strategy to reduce this burden, this is not yet available, reliable, or practical for the outcomes measured in this study [56]. WDYD was a stand-alone intervention and did not use in-person contact. Both in-person contact (eg, with a counselor) or a combined or stepped-care intervention (eg, mHealth combined with an educational program at school) could be promising ways to reduce dropout while also improving effectiveness [39,57,58]. In addition, although WDYD included pictures, videos, and a design that was tailored to accommodate low literacy levels, it relied on text to provide tailored feedback. As health literacy is related to dropout, future mHealth interventions could rely more on audio and video feedback [57]. Additionally, providing tailored information by a conversational agent using generative artificial intelligence (AI) could be a promising way to reduce dropout and increase intervention effects [59,60]. Possibly, the WDYD intervention was too static, thereby limiting engagement. Emerging developments in generative AI offer additional opportunities to personalize interventions at scale. AI-driven conversational agents can deliver dynamic, context-aware feedback, simulate motivational interviewing, and adapt content based on real-time user data. These technologies could enhance engagement, reduce attrition, and provide more nuanced support for behavior change compared to static tailoring. However, ethical considerations such as privacy, transparency, and bias mitigation must be addressed before widespread implementation in mHealth interventions.

Despite not finding significant effects on the primary outcomes, and dropout being a serious problem, WDYD could be seen as a promising intervention. This is especially true when taking into account that young and lower-educated adolescents and young adults are a difficult group to reach, as the majority (90%) lack motivation to reduce their alcohol consumption [27]. First, the combination of recruitment strategies used in this study showed that we are able to reach young adults who drink excessively; the use of social media advertisements resulted in the largest number of participants. Second, we found positive effects of WDYD on the secondary outcomes, intrinsic motivation, and self-confidence toward drinking less alcohol. Both outcomes significantly increased in the intervention group compared to the control group, and this effect lasted over 25 weeks. Third, additional analyses revealed that increases in intrinsic motivation and self-confidence among active users of WDYD were significantly associated with reductions in alcohol consumption (data not shown). WDYD can thus be an effective means to improve motivation and self-confidence toward reducing alcohol consumption, and this increase seems to have promising effects on alcohol consumption itself. In addition, the user-based assessments revealed that the intervention was evaluated very positively. WDYD was evaluated as usable (eg, user-friendly and easy in navigation), credible, understandable, and provided an adequate amount of information. The informativity and usefulness were evaluated as sufficient. Dynamic tailoring was successfully implemented in the intervention design, as participants indicated that WDYD accounted for their personal preferences, discussed topics they found important, and provided freedom in choosing their alcohol consumption. This was also the case for motivational interviewing because participants felt accepted for who they are and felt addressed pleasantly.

A small proportion of participants were recruited via channels that may have provided prior exposure to alcohol-related information, such as the online lifestyle monitor (327/6146, 5.3%) or educational programs delivered by teachers (272/6146, 4.4%). Although this may have reduced contrast between intervention and control conditions, moderation analyses showed that the recruitment method did not significantly influence group effects (data not shown).

### Limitations

This study had multiple limitations. First and foremost, dropout rates were high, particularly in the intervention group. This selective attrition may have introduced bias. To mitigate this, we applied multiple imputation [61]. However, due to difficulties imputing data from weeks 31 and 33 after baseline, these time points were omitted from the imputation model, and analyses were limited to data up to 25 weeks. Additionally, we did not examine reasons for dropout, which could have provided important information to reduce dropout in the future [57].

Second, although the intervention was originally developed for students in secondary vocational education, recruitment challenges led us to broaden our inclusion criteria to all Dutch adolescents and young adults engaging in excessive drinking. As a result, only 34.6% (1659/4795) of the sample consisted of students in secondary vocational education, and 6% (284/4795) had a low educational level. While moderation analyses showed no significant interaction between educational level and treatment effects (data not shown), caution is warranted when generalizing results to students in secondary vocational education or students from lower-educated populations. Sampling from a more diverse population may have introduced more variability in drinking behavior and decreased receptiveness to our intervention, which could affect the interpretation of our outcomes.

Third, a small proportion of participants (5.3%) were recruited via an online lifestyle monitor or via teachers delivering alcohol education (4.4%). These participants may have already been exposed to normative feedback or alcohol-related risk information before this study. This prior exposure could have reduced the contrast between the intervention and control conditions, potentially reducing observed effects.

Fourth, we used single-item measures to assess the primary and secondary outcomes. Although these items were selected based on prior studies and some of these items showed comparable or adequate performance compared to multi-item scales [42-44], single-item measures are generally more susceptible to measurement error and have lower psychometric quality [62]. In addition, the items used were not validated before our study, which may limit the reliability and generalizability of our findings. However, in studies using EMA, multiple-item scales may enhance reactivity and participant burden [63], justifying our choice for brevity.

Finally, there was a potential risk of contamination between conditions. Although participants were individually randomized into the control or experimental condition within the WDYD app, it is possible that participants in the control group were acquainted with those in the intervention group and discussed intervention content. To assess this, we asked participants in the control group 12 questions about the intervention at the first follow-up (9 weeks after baseline). The answers provided no strong indication of contamination. The distribution of correct answers resembled random guessing, and no participant answered more than 8 of 12 items correctly (data not shown).

### Strengths

Strong points of this study are the participatory design, use of intervention mapping as a framework for systematic development, the focus on a high-risk group for whom hardly any interventions are available, the use of EMAs to account for the fluctuating nature of alcohol consumption and to account for recall bias [26,50], and the use of multilevel multiple imputation analyses to account for dropout [61]. The current study adhered to the CONSORT (Consolidated Standards of Reporting Trials) statement [64,65], thereby improving the quality of reporting.

### Conclusions

The dynamically tailored mHealth intervention WDYD targeting adolescents and young adults with excessive alcohol consumption did not show favorable changes in alcohol intake, and dropout rates were high. Nevertheless, WDYD might be useful for adolescents and young adults to improve intrinsic motivation and self-confidence to reduce their alcohol consumption as important first steps toward behavior change. As such, the intervention has been implemented in the Netherlands as part of a larger campaign. To reduce dropout rates in mHealth interventions, future research should explore underlying reasons for dropout, as well as using in-person contact, a combined intervention (eg, mHealth with educational program), and audio- or visual-feedback to improve user retention.

## Supplementary material

10.2196/68468 Multimedia Appendix 1 Research information.

10.2196/68468 Multimedia Appendix 2 Screenshots of the WDYD intervention. WDYD: What Do You Drink.

10.2196/68468 Multimedia Appendix 3 Recruitment information.

10.2196/68468 Checklist 1 CONSORT-eHealth checklist WDYD intervention, October 23, 2025. WDYD: What Do You Drink.

## Abbreviations

AI: artificial intelligence
CONSORT: Consolidated Standards of Reporting Trials
EMA: ecological momentary assessment
mHealth: mobile health
WDYD: What Do You Drink
