# Supplementary material for: Effects, Acceptability, and Use of a Dynamically Tailored Mobile What Do You Drink Intervention to Reduce Excessive Drinking Among Adolescents and Young Adults in the Netherlands: Randomized Controlled Trial
Source: JMIR Mhealth Uhealth. 2026 May 26;14:e68468. doi: 10.2196/68468 (PMC13211942; doi:10.2196/68468)
Supplement: Multimedia Appendix 1 — Research information. [file mhealth-v14-e68468-s001.docx]

1. Research information within the WDYD app


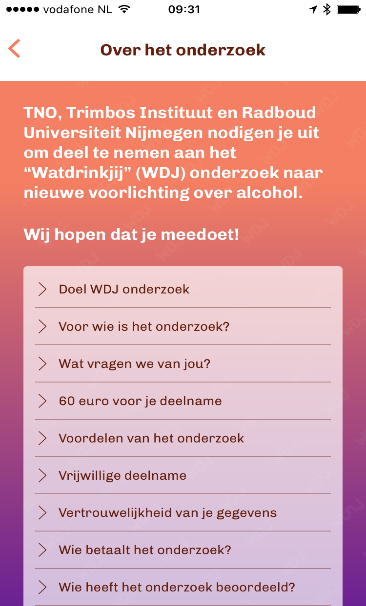

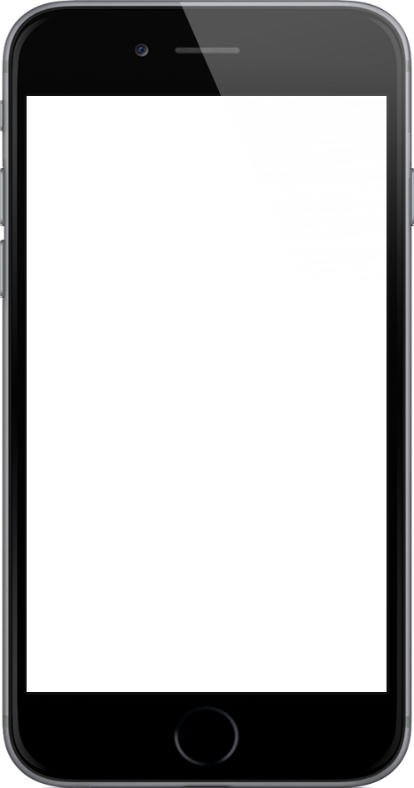

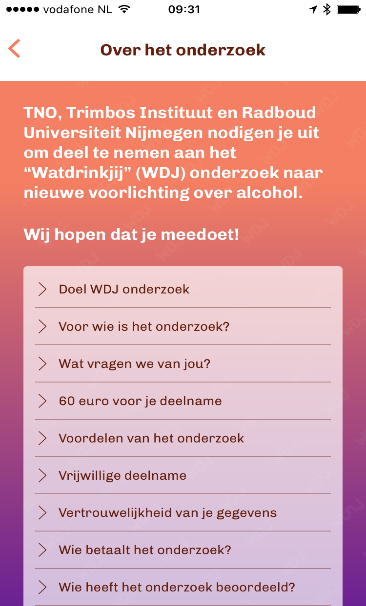

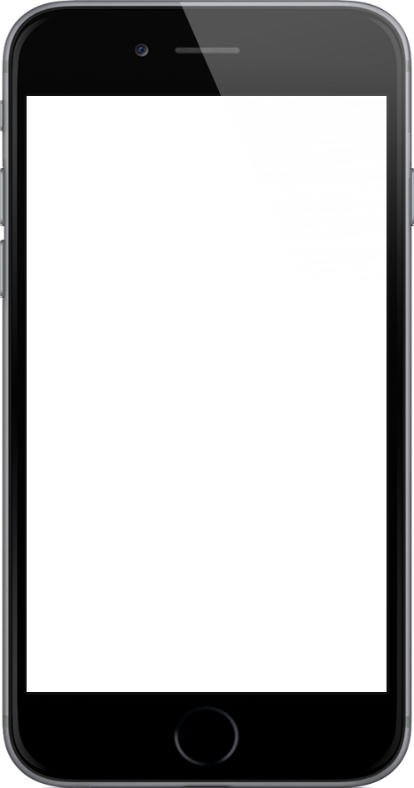


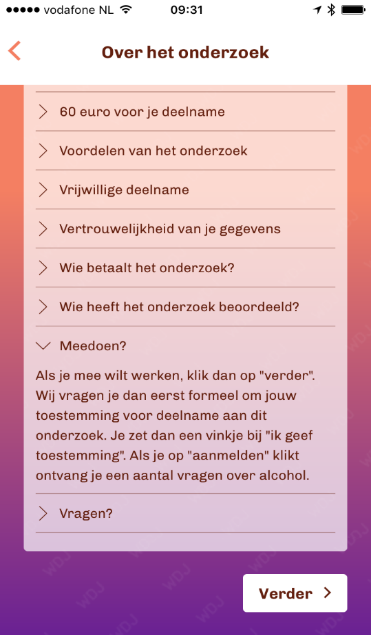


*English translation (a)*

**About the study**

TNO, Trimbos Institute, and Radboud University Nijmegen invite you to participate in the “WhatDoYouDrink” study on new education about alcohol. We hope you will join us!

Goal of the study

Who is the study for?

What do we ask of you?

60 euro compensation

Advantages of participation

Voluntary participation

Confidentiality of your data

Who funds the study?

Who has reviewed the study?

Interested? If you want to participate, click on ‘continue’. We will then formally ask for your consent to take part in this study. You will check the box ‘I give consent’. Once you click ‘register’, you will receive a few questions about alcohol.

Questions?

1. Research information for participants in the WDYD trial (names, telephone numbers and email addresses are replaced by ‘***’) **Watdrinkjij onderzoek**

TNO, Trimbos Instituut en Radboud Universiteit Nijmegen nodigen je uit om deel te nemen aan het “Watdrinkjij” (WDJ) onderzoek naar nieuwe voorlichting over alcohol.

Wij hopen dat je meedoet!

**Doel WDJ onderzoek**

We onderzoeken hoe we jongeren in de leeftijd van 16 tot en met 24 jaar persoonlijk kunnen voorlichten over alcohol. Dit advies is vrijblijvend, jij bent niet verplicht om minder te drinken. Voor dit onderzoek hebben we jouw hulp nodig.

**Voor wie is het onderzoek?**

Het onderzoek is bedoeld voor jongeren van 16 tot en met 24 jaar die wel eens alcohol drinken.

**Wat vragen we van jou?**

Het onderzoek vindt plaats via de WDJ app en duurt ongeveer 6 maanden. Alle deelnemers worden door loting over 2 groepen verdeeld. Alleen de deelnemers uit groep 1 ontvangen de nieuwe voorlichting, die uit groep 2 niet. Je kunt dus niet zelf kiezen in welke groep je komt.

Het onderzoek start met een korte vragenlijst; de vragen gaan over jouw ervaringen en mening over alcohol. Daarna ontvang je eens in de zes weken een week lang dagelijks een paar vragen over je alcoholgebruik en stemming; het invullen hiervan kost je hooguit 1 minuut per keer. Daarnaast ontvang je 2 en 6 maanden na de start een korte vragenlijst met vragen over jouw ervaringen en mening over alcohol; het invullen hiervan kost maximaal 15 minuten. Deelnemers uit groep 1 ontvangen hiernaast een aantal weken persoonlijke tips over alcohol.

**Beloning voor je deelname**

Als je volledig meedoet aan het onderzoek ontvang je minimaal €25,- en maximaal €80,- aan Bol.com bonnen. Dit bedrag wordt na afloop van het onderzoek uitgekeerd via je e-mailadres. Hoever het boven die 25 euro gaat uitkomen hangt af van het totaal aantal deelnemers dat het onderzoek volledig afrond. Hoe minder deelnemers het onderzoek afronden, hoe meer je verdient.
Via de app stellen we vragen over je alcoholgebruik en geven we soms tips. Na 2 en na 6 maanden krijg je een korte vragenlijst. Per vragenlijst verdien je €12,50. Heb je ze beide ingevuld dan ben je verzekerd van €25,-. Afhankelijk van het aantal personen dat volledig heeft meegewerkt kan dat bedrag oplopen tot maximaal €80,-.

**Voordelen van het onderzoek**

Door deel te nemen aan het onderzoek draag je bij aan het verbeteren van de voorlichting aan jongeren over het drinken van alcohol. Als je wilt, informeren we je na afloop wat het onderzoek over de resultaten.

**Vrijwillige deelname**

Je bent niet verplicht om aan het onderzoek deel te nemen. Ook kun je op ieder moment stoppen, zonder uitleg waarom je stopt. Wij hopen natuurlijk dat je blijft meedoen, want alleen dan is jouw inbreng van waarde voor verbetering van de voorlichting.

**Vertrouwelijkheid van je gegevens**

Alle gegevens, die tijdens het onderzoek over jou verzameld worden, zijn vertrouwelijk. Onderzoekers van het project kunnen de gegevens wel bekijken, maar je naam blijft voor hen onbekend. De gegevens worden niet gedeeld met anderen. Over het onderzoek verschijnen wetenschappelijke publicaties, maar ook daarin blijven de gegevens van de deelnemers volledig anoniem.

**Wie betaalt het onderzoek?**

Het onderzoek wordt betaald door Zorg Onderzoek Nederland en Medische Wetenschappen van NWO (ZonMw). ZonMw stimuleert namens de overheid onderzoek om de gezondheidszorg van Nederland te verbeteren.

Aan het onderzoek zijn voor jou geen kosten verbonden.

**Wie heeft het onderzoek beoordeeld?**

Het onderzoek is voorgelegd aan de Ethiek Commissie Faculteit der Sociale Wetenschappen van Radboud Universiteit Nijmegen. De commissie heeft geoordeeld dat het onderzoek aan alle ethische eisen voldoet die gelden voor een dergelijk onderzoek in Nederland, waaronder de bescherming van de veiligheid en anonimiteit van de deelnemers.

**Klachten**

Mocht je klachten hebben over het onderzoek, dan kun je contact opnemen met Dr. ***, Teamleider en onderzoeker, TNO Child Health, telefoonnummer ****.

**Meedoen?**

Als je mee wilt doen, klik dan op “verder”. Wij vragen je dan eerst formeel om jouw toestemming voor deelname aan dit onderzoek. Je zet dan een vinkje bij “ik geef toestemming”. Als je op “aanmelden” klikt, ontvang je een aantal vragen over alcohol.

**Contact**

Mocht je nog vragen hebben, dan zijn wij graag bereid je te woord te staan. Je kunt je vragen stellen aan ***, en haar telefonisch bereiken op telefoonnummer *** of mailen via ***.

Wij hopen dat je meedoet!

*English translation (b)*

**WhatDoYouDrink study**

TNO, Trimbos Institute and Radboud University Nijmegen invite you to take part in the WhatDoYouDrink (WDJ) study on new alcohol education. We hope you will join us!

**Purpose of the WDJ study**

We are exploring how to provide personalized alcohol education to young people aged 16 to 24 years. This advice is non-binding. You are not required to drink less. But we do need your help to carry out this study.

**Who is the study for?**

The study is intended for young people aged 16 to 24 years who occasionally drink alcohol.

**What do we ask of you?**

The study takes place via the WDJ app and lasts about 6 months. All participants are randomly assigned to one of two groups. Only participants in Group 1 receive the new alcohol education; Group 2 does not. You cannot choose which group you are in.

The study begins with a short questionnaire about your experiences and opinion on alcohol. Then, every six weeks, you will receive a week-long series of daily questions about your alcohol use and mood, each set takes no more than one minute to complete.

Additionally, you will receive a short questionnaire at 2 and 6 months after starting, asking again about your experiences and opinion on alcohol. These take up to 15 minutes each. Participants in Group 1 will also receive personalized tips about alcohol for several weeks.

**Compensation for participation**

If you complete the study, you will receive between 25 and 80 euros in bol.com vouchers. This amount will be sent to you email after the study ends. The final amount depends on how many participants complete the study – the fewer who finish, the more you earn.

Through the app, we will ask questions about your alcohol use and occasionally offer tips. After 2 and 6 months, you will receive a short questionnaire. Each completed questionnaire earns you 12,50 euro. If you complete both, you are guaranteed 25 euro. Depending on the total number of participants, this can increase to a maximum of 80 euro.

**Benefits of participation**

By joining the study, you help improve alcohol education for young people. If you would like, we will share the study results with you afterward.

**Voluntary participation**

You are not required to take part in the study. You can also stop at any time, without needing to explain why. Of course, we hope you will stay involved – your input is valuable for improving alcohol education.

**Confidentiality of your data**

All data collected about you during the study is confidential. Project researchers can view the data, but your name will remain unknown to them. Your data will not be shared with others. Scientific publications may result from the study, but participant data will remain fully anonymous.

**Who funds the study?**The study is funded by ZonMw, the Netherlands Organization for Health Research and Development. ZonMw promotes research on behalf of the government to improve healthcare in the Netherlands. There are not costs for you to participate.

**Who reviewed the study?**

The study was reviewed by the Ethics Committee of the Faculty of Social Sciences at Radboud University Nijmegen. The committee conformed that the study meets all ethical standards required in the Netherlands, including the protection of participant safety and anonymity.

**Complaints**

If you have any complaints about the study, you can contact Dr [Name], team leader and researcher at TNO Child Health, by phone at [phone number].

**Want to participate?**

If you would like to participate, click ‘continue’. We will first ask for you formal consent to participate. Check the box ‘I give consent’. Then click ‘register’ to receive a set of questions about alcohol.

**Contact**

If you have questions, we are happy to help. You can reach out to [name] by phone at [phone number] or by email at [email address].

We hope you will join us!

1. Informed consent within WDYD


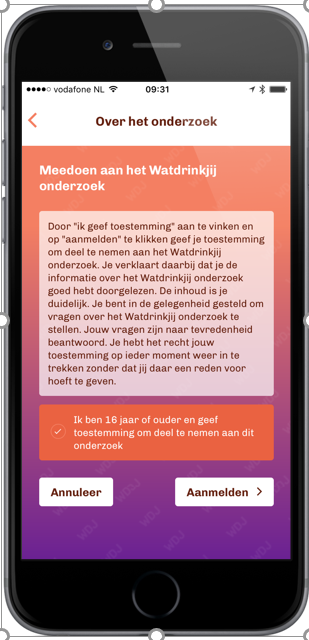


*English translation (c):*

**About the study**

Participation in the WhatDoYouDrink study

By checking the box ‘I give consent’, you will provide consent to participate in the WhatDoYouDrink study. You declare that you have thoroughly read the information about the WhatDoYouDrink study. The content of the study is clear. You were able to ask questions about the WhatDoYouDrink study. Your questions were answered to your satisfaction. You have the right to withdraw your consent at any time without providing an explanation.

- I am 16 years or older and give consent to participate in this study.

Cancel Register >

1. Registration within WDYD


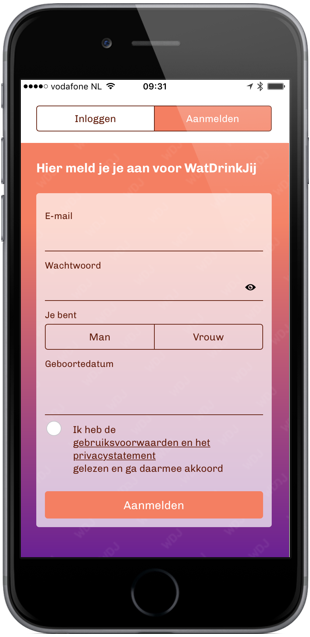


*English translation (d)*

Here you can register for WhatDoYouDrink

Email

Password

You are: male / female

Birthdate

- I have read the terms of use and privacy statement and agree with these.

Register

1. Terms of use and privacy statement as part of the registration procedure (personal information is replaced by ***)

**Gebruiksvoorwaarden en Privacyverklaring Watdrinkjij (WDJ) App**

**Gebruiksvoorwaarden WDJ app**
Versie 28 augustus 2018

**Artikel 1. Definities**TNO: Onderzoeksinstituut TNO, Afdeling Child Health, gevestigd aan de Schipholweg 77-79 (2316 ZL) in Leiden, e-mail: ***.

Trimbos: Stichting Trimbos-Instituut, Netherlands Institute of Mental Health and Addiction, gevestigd aan de Da Costakade 45 (3521 VS) in Utrecht, e-mail: info@trimbos.nl, KvK-nummer: 41265454.

App: de WDJ App die wordt aangeboden door TNO en Trimbos.

Gebruiker: een gebruiker van de App.

Content: alle elementen die onderdeel uitmaken van de App, waaronder teksten, foto’s, data, logo’s en software.

Intellectuele Eigendomsrechten: alle intellectuele eigendomsrechten, waaronder auteursrechten, naburige rechten, handelsnaamrechten, merkenrechten, rechten met betrekking tot know how en octrooien.

**Artikel 2. Toepassing voorwaarden**
Wanneer een Gebruiker de App downloadt of gebruikt, zijn deze algemene voorwaarden van toepassing.

**Artikel 3. Toestemming voor gebruik**
De Gebruiker verkrijgt toestemming om voor persoonlijke, niet-commerciële doeleinden gebruik te maken van de verschillende functionaliteiten van de App. Het is de gebruiker niet toegestaan om de App of de Content die daar onderdeel van uitmaakt te (her)gebruiken voor enige andere doeleinden. Meer specifiek betekent dat onder meer dat het de Gebruiker niet is toegestaan om (een deel van) de App of (een deel van) de Content te kopiëren, te wijzigen of met derden te delen, behoudens voor zover dat is voorzien in de functionaliteiten van de App. TNO en Trimbos zijn en blijven eigenaar van alle Intellectuele Eigendomsrechten ten aanzien van de App en de Content.

**Artikel 4. Respecteren rechten derden**
De Gebruiker garandeert dat hij geen Content zal plaatsen die inbreuk maakt op Intellectuele Eigendomsrechten of die om andere redenen onrechtmatig is jegens derden of TNO en Trimbos.

**Artikel 5. Aansprakelijkheid**
5.1 De App is ontwikkeld door TNO, Trimbos en de Radboud Universiteit Nijmegen en kan inzicht geven in je alcoholgebruik en geeft je persoonlijk advies. Op basis van de antwoorden die je geeft, selecteert de computer de juiste adviesteksten die passen bij je situatie. De adviesteksten zijn gebaseerd op de huidige wetenschappelijke inzichten. Het advies wordt vrijblijvend gegeven, je bent niet verplicht om de adviezen op te volgen. De App heeft in geen geval de diagnose, bewaking of behandeling van ziekten tot doel. De App dient daarom niet te worden beschouwd als een vervanging voor een consult van of een behandeling door een arts.

5.2 TNO en Trimbos behouden zich het recht voor om de App op ieder moment zonder opgaaf van redenen uit app-stores te verwijderen en ontoegankelijk te maken voor Gebruikers, of voor een specifieke Gebruiker, indien TNO en Trimbos daar aanleiding toe zien.

5.3 TNO en Trimbos zijn niet aansprakelijk voor eventuele schade die ontstaat door het gebruik van de App, informatie die als onderdeel van de App ter beschikking wordt gesteld of het verwijderen of ontoegankelijk maken van de App zoals hiervoor omschreven, tenzij deze schade is ontstaan door opzet of grove schuld van TNO en Trimbos.

5.4 De Gebruiker garandeert dat hij bij het gebruik van de App niet in strijd met deze algemene voorwaarden zal handelen en hij vrijwaart TNO en Trimbos ten aanzien van alle mogelijke vorderingen van derden welke het gevolg zijn van handelingen die in strijd zijn met de bepalingen uit deze algemene voorwaarden.

**Artikel 6. Privacy**
In het kader van het gebruik van de App zal de Gebruiker bepaalde persoonsgegevens aan TNO en Trimbos ter beschikking stellen. Op de verwerking van deze gegevens is de Privacyverklaring die bij deze App hoort van toepassing, die hieronder kan worden geraadpleegd.

**Artikel 7. Wijzigingen**
TNO en Trimbos kunnen deze algemene voorwaarden eenzijdig wijzigen of aanvullen. De aangepaste algemene voorwaarden zullen dan in onze App ter beschikking worden gesteld en er zal in de App een melding worden geplaatst dat de algemene voorwaarden zijn gewijzigd.

**Artikel 8. Rechts- en forumkeuze**
Op deze algemene voorwaarden en alle geschillen met betrekking tot het gebruik van de App is Nederlands recht van toepassing. Geschillen zullen worden voorgelegd aan de bevoegde rechter bij de rechtbank Midden-Nederland.

**Privacyverklaring WDJ app**In deze privacyverklaring kun je informatie vinden over hoe er wordt omgegaan met (persoons)gegevens die je achterlaat als je een account aanmaakt en bij het gebruiken van de WDJ App (hierna: “de App”). Je persoonsgegevens worden verwerkt door TNO, Afdeling Child Health, gevestigd aan de Schipholweg 77-79 (2316 ZL) in Leiden, telefoonnummer ***, e-mail: *** (hierna: “TNO”) en het Trimbos-Instituut, gevestigd aan de Da Costakade 45 (3521 VS) in Utrecht, telefoonnummer ***, e-mail: *** (hierna: “Trimbos”).

Als je na het lezen van deze privacyverklaring nog vragen hebt over de wijze waarop TNO of Trimbos omgaat met je persoonsgegevens, als je gebruik wilt maken van je rechten op grond van de AVG (die hieronder zijn beschreven), of als je een klacht wil indienen over het gebruik van je persoonsgegevens, kun je mailen naar ***. Als je niet tevreden bent over hoe TNO je klacht afhandelt of als je je klacht liever niet bij TNO indient, kun je deze ook indienen bij de Autoriteit Persoonsgegevens.

Deze privacyverklaring bevat de volgende informatie:

1.       Waarvoor dient de App?

2.       Wie is verantwoordelijk voor de verwerking van je persoonsgegevens?

3.       Welke persoonsgegevens worden door ons verzameld?

4.       Met welk doel en op welke grond worden je persoonsgegevens verwerkt?

5.       Verzamelen wij gegevens over kinderen jonger dan 16?

6.       Hoe lang bewaren wij jouw gegevens?

7.       Met wie delen wij jouw gegevens?

8.       Hoe beveiligen wij je persoonsgegevens?

9.       Wat zijn je rechten?

10.   Wijzigingen

**1. Waarvoor dient de App?**
De App is er om te onderzoeken hoe we jongeren in de leeftijd van 16 tot en met 24 jaar persoonlijk kunnen voorlichten over alcohol. Alle deelnemers worden door loting over 2 groepen verdeeld. Alleen de deelnemers uit groep 1 ontvangen de nieuwe voorlichting, die uit groep 2 niet. Je kunt dus niet zelf kiezen in welke groep je komt.

**2. Wie is verantwoordelijk voor de verwerking van je persoonsgegevens?**
TNO is verantwoordelijk voor de verwerking van jouw persoonsgegevens zoals hieronder beschreven en zal daarbij de grootst mogelijke zorgvuldigheid betrachten. Wij doen er uiteraard alles aan om de privacyregels die voortvloeien uit de Algemene Verordening Gegevensbescherming en andere wet- en regelgeving na te leven.

**3. Welke persoonsgegevens worden door ons verzameld?**
Als je een account aanmaakt om de App te gebruiken of als je de App gaat gebruiken, vragen wij je om de volgende persoonsgegevens:

- je e-mailadres en een wachtwoord
- je geboortedatum
- je geslacht
- de cijfers van je postcode
- je herkomst (je geboorteland en die van je ouders)

Verder verzamelen wij gegevens die je in de App invult, zoals antwoorden op een vragenlijst over je mening over alcoholgebruik, je dagboekgegevens, je voorkeursinstellingen en antwoorden/opties die je kiest. Aangezien deze gegevens informatie bevatten over hoe jij je voelt en je herkomst is sprake van gevoelige of bijzondere persoonsgegevens. Tenslotte bewaren we gegevens over hoe jij de App gebruikt (bijvoorbeeld over hoe vaak je de App gebruikt en welke onderdelen).

Je kunt de App alleen gebruiken als je een account hebt aangemaakt. Je kan een account aanmaken door je e-mailadres en een wachtwoord op te geven. Als je anoniem wil blijven raden we je aan om e-mailadres te gebruiken waar niet je echte naam in voorkomt. Helemaal anoniem blijven kan niet: hoewel wij niet kunnen zien wie je bent als je een e-mailadres zonder naam gebruikt is via de provider vaak wel te achterhalen via welk IP adres een e-mailadres is aangemaakt. Als je geen e-mailadres wil opgeven kun je de App helaas niet gebruiken.

**4. Met welk doel en op welke grond worden je persoonsgegevens verwerkt?**
We gebruiken je e-mailadres om je in te laten loggen op jouw account en om je een nieuw wachtwoord te sturen als je je wachtwoord bent vergeten. Daarnaast gebruiken we je e-mailadres om je een beloning (Bol.com bon) voor je deelname aan het WDJ onderzoek te sturen. Je e-mailadres wordt binnen 4 weken na uitkering van de beloning verwijderd. We sturen je geen andere informatie per e-mail (tenzij je daar specifiek om hebt verzocht of ons een vraag hebt gesteld per e-mail).

Nadat je de app hebt gedownload en je hebt aangemeld, wordt er een koppeling gemaakt tussen jouw account en je smartphone. Daardoor is het mogelijk om pushberichten zoals herinneringen te sturen die onderdeel zijn van de App.

Wij gebruiken je geboortedatum, je geslacht, de cijfers van je postcode en je herkomst om inzicht te krijgen in de doelgroep van de App. Je geboortedatum wordt omgezet in je leeftijd, en daarna verwijderd uit het databestand. Met deze gegevens krijgen wij inzicht in welke leeftijdscategorieën de App gebruiken, hoe gebruikers over de regio’s in Nederland verspreid zijn, en hoeveel mannen en vrouwen hebben meegedaan, hoeveel daarvan een Nederlandse achtergrond hebben, en welke onderdelen van de App door welke groep worden gebruikt. Zo kunnen we beter uitspraken doen over de werking van de App, en de App in de toekomst beter en gebruiksvriendelijker inrichten.

De gegevens uit de vragenlijsten, dagboeken en het gebruik van de App worden gebruikt om op groepsniveau uitspraken te doen over de effectiviteit van de nieuwe voorlichting. Als jij in de groep terecht komt die de nieuwe voorlichting ontvangt binnen de App, krijg je de mogelijkheid om oefeningen te maken. Dit zijn functionaliteiten die je kunt gebruiken als jij dat wilt. We slaan hiervan alleen op welke oefeningen je kiest, en niet de notities die je binnen de oefeningen maakt.

Wij zien gegevens over hoe jij je voelt en je herkomst als gevoelige of bijzondere persoonsgegevens en hebben jouw toestemming nodig voor het verwerken daarvan.

De grondslag voor het verwerken van deze gegevens is jouw toestemming. Je kunt deze toestemming ook weer intrekken door ons een e-mail te sturen via *** onder vermelding van het betreffende e-mailadres waaraan jouw account gekoppeld is. Wij verwijderen dan tot jou herleidbare gegevens binnen vier weken uit ons bestand. Als je je toestemming intrekt kan je helaas geen gebruik meer maken van de App. Het gebruik van de persoonsgegevens tot het moment van intrekking blijft rechtmatig.

**5. Verzamelen wij gegevens over kinderen jonger dan 16?**
Nee. De App is niet bedoeld voor kinderen jonger dan 16. Als je een geboortedatum of leeftijd invult waaruit blijkt dat je jonger dan 16 jaar bent, kun je de App niet gebruiken.

Wij kunnen helaas niet controleren of iemand liegt over zijn leeftijd, waardoor het voor zou kunnen komen dat wij toch gegevens ontvangen die zijn achtergelaten door iemand die de leeftijd van 16 jaar nog niet heeft bereikt. Mocht je aanleiding hebben te denken dat dit het geval is, neem dan contact met ons op zodat we de betreffende gegevens kunnen verwijderen.

**6. Hoe lang bewaren wij jouw gegevens?**
Wij bewaren jouw persoonsgegevens tot uiterlijk vier weken na ontvangst van een emailbericht van jou waarin je aangeeft dat jij je account wilt verwijderen. Je kunt ons daarvoor een e-mail sturen via *** onder vermelding van het betreffende e-mailadres waaraan jouw account gekoppeld is. Als je dit verzoek niet doet, wordt je e-mailadres binnen 4 weken na uitkering van de beloning verwijderd; dit geldt ook voor je geboortedatum. Daarna bewaren we enkel nog het geanonimiseerde databestand met gegevens uit de vragenlijsten, over jouw gebruik van de App en de gegevens die je achterlaat op de App. Daar staan dus geen tot jou herleidbare gegevens meer in. Deze gegevens bewaren we maximaal 7 jaar na de laatste publicatie van de onderzoeksresultaten.

**7. Met wie delen wij jouw gegevens?**
We slaan jouw gegevens op bij onze ICT-serviceprovider, die daarmee jouw gegevens verwerkt in onze opdracht. De gegevens worden opgeslagen op een server binnen de Europese Unie. Met deze partij hebben wij afspraken gemaakt (een verwerkersovereenkomst) om de gegevens passend te beveiligen tegen verlies, diefstal, misbruik, ongeoorloofde toegang en ongewenste wijziging of verstrekking.

We verstrekken je gegevens verder niet aan derden, tenzij TNO en Trimbos op grond van geldende wet- en regelgeving verplicht wordt bepaalde gegevens te verstrekken, bijvoorbeeld aan de politie in het kader van een opsporingsonderzoek.

**8. Hoe beveiligen we jouw gegevens?**
Wij hebben passende technische en organisatorische veiligheidsmaatregelen getroffen om je persoonsgegevens te beschermen tegen verlies, misbruik en ongeoorloofde toegang door derden. Zo worden de gegevens op een beveiligde server opgeslagen. Jouw wachtwoord wordt versleuteld opgeslagen. Jouw e-mailadres en de overige door jou in de App ingevoerde gegevens worden gekoppeld door een code en vervolgens van elkaar gescheiden bewaard. Alleen de ICT-serviceprovider heeft toegang tot beide bestanden. De onderzoekers van het project WDJ hebben alleen toegang tot de geanonimiseerde gegevens. Daarnaast zal TNO eenmalig de e-mailadressen bij de ICT-serviceprovider opvragen van diegenen die de beloning uitgekeerd krijgen, maar ook dan kan TNO niet zien welke e-mailadressen bij welke antwoorden in de App horen. Wij verplichten onze ICT-serviceprovider passende technische en organisatorische veiligheidsmaatregelen te treffen.

**9. Wat zijn je rechten?**
Behalve dat je elk moment je toestemming voor de verwerking van persoonsgegevens mag intrekken, zoals hierboven beschreven, heb je:

a)      Het recht op inzage in je persoonsgegevens en het ontvangen van een kopie daarvan;

b)      Het recht op rectificatie van je persoonsgegevens indien deze niet juist of onvolledig zijn;

c)       Het recht om bezwaar te maken tegen de verwerking of – in bepaalde gevallen - het recht op beperking van de verwerking van je persoonsgegevens;

d)      In bepaalde gevallen: het recht om je persoonsgegevens te laten wissen (‘recht op vergetelheid’), bijvoorbeeld als je je toestemming voor het gebruik van je persoonsgegevens intrekt;

e)      Het recht om je persoonsgegevens in een gestructureerde, gangbare en machinaal leesbare vorm te verkrijgen en die gegevens aan een ander over te dragen.

Voor meer informatie: zie artikelen 15 tot en met 20 van de Algemene Verordening Gegevensbescherming.

Je kan je rechten uitoefenen door per e-mail contact op te nemen met de hierboven genoemde contactpersoon.

Houd er bij verwijdering van je persoonsgegevens of de beperking van de verwerking van je persoonsgegevens wel rekening mee dat de App daarna niet of niet in zijn geheel meer kan worden gebruikt.

Indien wij je e-mailadres al hebben verwijderd kunnen wij geen gevolg meer geven aan een verzoek tot uitoefening van je rechten, omdat wij dan niet meer weten welke gegevens bij jou horen. In dat geval is er ook geen sprake meer van persoonsgegevens in de zin van de Algemene Verordening Gegevensbescherming.

**Wijzigingen**
Onze diensten zijn voortdurend in ontwikkeling. Wij behouden ons daarom het recht voor om deze privacyverklaring van tijd tot tijd te wijzigen of aan te vullen en plaatsen eventuele gewijzigde versies in onze App. We plaatsen een opvallende melding in de App om je te informeren indien de privacyverklaring is gewijzigd en geven bovenaan de privacyverklaring aan wanneer deze de laatste keer is bijgewerkt. We raden je aan om de nieuwste versie regelmatig te raadplegen en indien je contact met ons op wil nemen te bekijken of de contactgegevens nog up-to-date zijn. De nieuwe privacyverklaring is na plaatsing van kracht.

Versie 2.0 Datum 28 augustus 2018

*English translation (e)*

## ****Terms of Use – WDJ App****

**Version: August 28, 2018**

### ****Article 1. Definitions****

- **TNO**: Research institute TNO, Department of Child Health, located at Schipholweg 77–79 (2316 ZL) in Leiden, email: ***.
- **Trimbos**: Trimbos Institute, Netherlands Institute of Mental Health and Addiction, located at Da Costakade 45 (3521 VS) in Utrecht, email: info@trimbos.nl, Chamber of Commerce number: 41265454.
- **App**: The WDJ App offered by TNO and Trimbos.
- **User**: A user of the App.
- **Content**: All elements that are part of the App, including texts, photos, data, logos, and software.
- **Intellectual Property Rights**: All intellectual property rights, including copyrights, neighboring rights, trade name rights, trademark rights, rights related to know-how, and patents.

### ****Article 2. Applicability of Terms****

These general terms apply when a User downloads or uses the App.

### ****Article 3. Permission to Use****

The User is granted permission to use the App’s features for personal, non-commercial purposes. The User is not allowed to reuse or repurpose the App or its Content for any other purpose. Specifically, the User may not copy, modify, or share (parts of) the App or its Content, except as permitted by the App’s functionalities. TNO and Trimbos retain ownership of all Intellectual Property Rights related to the App and its Content.

### ****Article 4. Respecting Third-Party Rights****

The User guarantees not to upload any Content that infringes on Intellectual Property Rights or is otherwise unlawful toward third parties or TNO and Trimbos.

### ****Article 5. Liability****

**5.1** The App was developed by TNO, Trimbos, and Radboud University Nijmegen. It provides insight into your alcohol use and offers personalized advice. Based on your answers, the system selects advice texts tailored to your situation. These texts are based on current scientific knowledge. The advice is non-binding; you are not required to follow it. The App is not intended to diagnose, monitor, or treat any medical condition and should not be considered a substitute for medical consultation or treatment.

**5.2** TNO and Trimbos reserve the right to remove the App from app stores or make it inaccessible to Users at any time and without explanation, if deemed necessary.

**5.3** TNO and Trimbos are not liable for any damages resulting from the use of the App, the information provided within it, or its removal or inaccessibility, unless such damage is caused by intent or gross negligence.

**5.4** The User guarantees to comply with these terms and indemnifies TNO and Trimbos against any third-party claims resulting from violations of these terms.

### ****Article 6. Privacy****

In using the App, the User provides certain personal data to TNO and Trimbos. The processing of this data is governed by the Privacy Statement associated with the App, which can be found below.

### ****Article 7. Changes****

TNO and Trimbos may unilaterally modify or supplement these terms. Updated terms will be made available in the App, and a notification will appear indicating that changes have been made.

### ****Article 8. Governing Law and Jurisdiction****

Dutch law applies to these terms and all disputes related to the App. Disputes will be submitted to the competent court in the district of Midden-Nederland.

## ****Privacy Statement – WDJ App****

This privacy statement explains how your (personal) data is handled when you create an account and use the WDJ App (“the App”). Your personal data is processed by:

- **TNO**, Department of Child Health, Schipholweg 77–79 (2316 ZL) in Leiden, phone: ***, email: ***
- **Trimbos Institute**, Da Costakade 45 (3521 VS) in Utrecht, phone: ***, email: ***

If you have questions after reading this privacy statement, want to exercise your rights under the GDPR (listed below), or wish to file a complaint about the use of your personal data, you can email ***. If you’re not satisfied with how TNO handles your complaint or prefer not to submit it to TNO, you may also contact the Dutch Data Protection Authority (Autoriteit Persoonsgegevens).

### This privacy statement includes the following information:

1. What is the purpose of the App?
2. Who is responsible for processing your personal data?
3. What personal data do we collect?
4. For what purpose and on what legal basis is your data processed?
5. Do we collect data from children under 16?
6. How long do we store your data?
7. With whom do we share your data?
8. How do we protect your personal data?
9. What are your rights?
10. Changes

### ****1. What is the purpose of the App?****

The App is designed to study how we can provide personalized alcohol education to young people aged 16 to 24. All participants are randomly assigned to one of two groups. Only participants in Group 1 receive the new educational content; those in Group 2 do not. You cannot choose which group you are placed in.

### ****2. Who is responsible for processing your personal data?****

TNO is responsible for processing your personal data as described below and will handle it with the utmost care. We make every effort to comply with privacy regulations under the General Data Protection Regulation (GDPR) and other applicable laws.

### ****3. What personal data do we collect?****

When you create an account or use the App, we ask for the following personal data:

- Your email address and password
- Your date of birth
- Your gender
- The digits of your postal code
- Your background (country of birth and that of your parents)

We also collect data you enter in the App, such as:

- Answers to questionnaires about your views on alcohol use
- Diary entries
- Your preferences and selected options

Because this data includes information about your feelings and background, it qualifies as sensitive personal data. We also store data about how you use the App (e.g., frequency and which features you use).

You can only use the App if you create an account with an email address and password. If you wish to remain anonymous, we recommend using an email address that does not contain your real name. Full anonymity is not possible: although we cannot see who you are, your email provider may be able to trace the IP address used to create the account. If you do not wish to provide an email address, you cannot use the App.

### ****4. Why and on what legal basis is your personal data processed?****

We use your email address to:

- Log you into your account
- Send you a new password if you forget yours
- Send you a Bol.com voucher as a reward for participating in the WDJ study

Your email address will be deleted within four weeks after the reward is issued. We do not send other emails unless you specifically request information or contact us.

Once you’ve downloaded and registered in the App, your account is linked to your smartphone, allowing us to send push notifications such as reminders.

We use your date of birth, gender, postal code digits, and background to understand the App’s target audience. Your date of birth is converted to age and then deleted. This helps us analyze age groups, regional distribution, gender participation, and cultural background, and how different groups use the App. This information helps us improve the App’s effectiveness and usability.

Data from questionnaires, diaries, and App usage is used to assess the effectiveness of the new educational content at a group level. If you are in the group that receives the new content, you may access optional exercises. We only record which exercises you choose, not the notes you write within them.

Because your emotional data and background are considered sensitive, we require your consent to process them.

The legal basis for processing this data is your consent. You may withdraw your consent at any time by emailing us at *** and referencing the email address linked to your account. We will delete all identifiable data within four weeks. If you withdraw consent, you can no longer use the App. Data processed before withdrawal remains lawful.

### ****5. Do we collect data from children under 16?****

No. The App is not intended for children under 16. If you enter a birthdate or age indicating you are under 16, you cannot use the App.

We cannot verify if someone lies about their age, so it’s possible we may receive data from someone under 16. If you suspect this has happened, please contact us so we can delete the relevant data.

### ****6. How long do we store your data?****

We store your personal data for up to four weeks after receiving an email from you requesting account deletion. You can email us at *** and reference the email address linked to your account.

If you do not make this request, your email address and birthdate will be deleted within four weeks after the reward is issued. After that, only anonymized data from questionnaires, App usage, and entries will be retained. This data is no longer traceable to you and will be stored for up to seven years after the final publication of the research results.

### ****7. Who do we share your data with?****

Your data is stored by our IT service provider, who processes it on our behalf. The data is stored on a server within the European Union. We have a data processing agreement with this provider to ensure appropriate protection against loss, theft, misuse, unauthorized access, and unwanted changes or disclosures.

We do not share your data with third parties unless required by law, such as in a police investigation.

### ****8. How do we protect your data?****

We have implemented appropriate technical and organizational measures to protect your personal data from loss, misuse, and unauthorized access. Your data is stored on a secure server. Your password is encrypted. Your email address and other data entered in the App are linked by a code and stored separately. Only the IT service provider has access to both files. WDJ researchers only access anonymized data.

TNO will request email addresses from the IT provider once to issue rewards, but cannot link email addresses to specific App responses. We require our IT provider to maintain strict security standards.

### ****9. What are your rights?****

In addition to withdrawing your consent at any time, you have the right to: a) Access your personal data and receive a copy b) Correct inaccurate or incomplete data c) Object to or restrict data processing in certain cases d) Request deletion of your data (“right to be forgotten”), e.g., if you withdraw consent e) Receive your data in a structured, commonly used, machine-readable format and transfer it to another party

For more information, see Articles 15–20 of the GDPR.

You can exercise your rights by emailing the contact person listed above. Please note that deleting or restricting your data may limit or disable your use of the App.

If we have already deleted your email address, we cannot fulfill further data requests, as we can no longer identify which data belongs to you. In that case, the data is no longer considered personal under the GDPR.

### ****Changes****

Our services are continuously evolving. We reserve the right to modify or update this privacy statement and will publish any changes in the App. A prominent notice will appear in the App when the privacy statement is updated, and the latest update date will be shown at the top. We recommend checking the latest version regularly and verifying that contact details are still current. The new privacy statement takes effect upon publication.

**Version 2.0 – Date: August 28, 2018**
