# Supplementary material for: Effects, Acceptability, and Use of a Dynamically Tailored Mobile What Do You Drink Intervention to Reduce Excessive Drinking Among Adolescents and Young Adults in the Netherlands: Randomized Controlled Trial
Source: JMIR Mhealth Uhealth. 2026 May 26;14:e68468. doi: 10.2196/68468 (PMC13211942; doi:10.2196/68468)
Supplement: Multimedia Appendix 2 — Screenshots of the WDYD intervention. WDYD: What Do You Drink. [file mhealth-v14-e68468-s002.docx]

1. Normative tailored feedback (b) Question to set a alcohol reduction goal


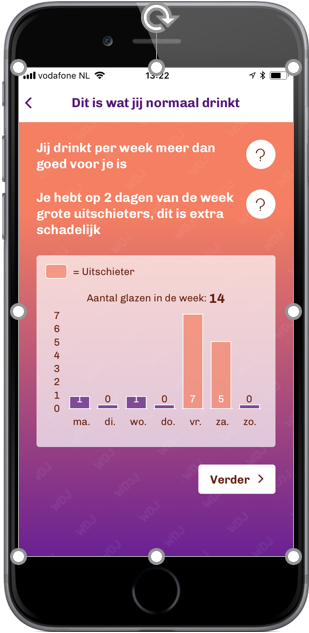

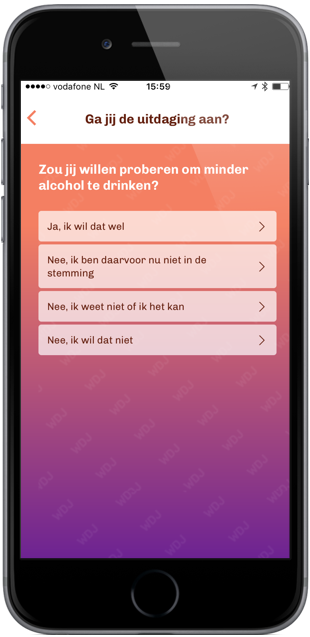


*English translation (a)*

**This is what you usually drink**

You drink more per week than is good for you.

You show significant peaks on two days per week, which poses additional health risks.

Peaks / Number of glasses during the week: 14.

*English translation (b)*

**Are you up for the challenge?**

Would you like to try drinking less alcohol?

Yes, I would like to

No, I am not in the mood for that

No, I am not sure I can do that

No, I do not want to

(c) A check whether the drinking (d) The diary

goal is achievable


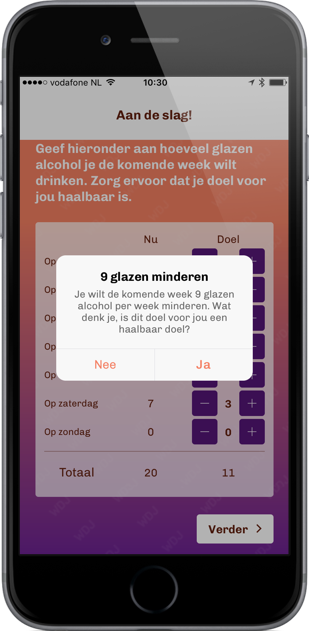

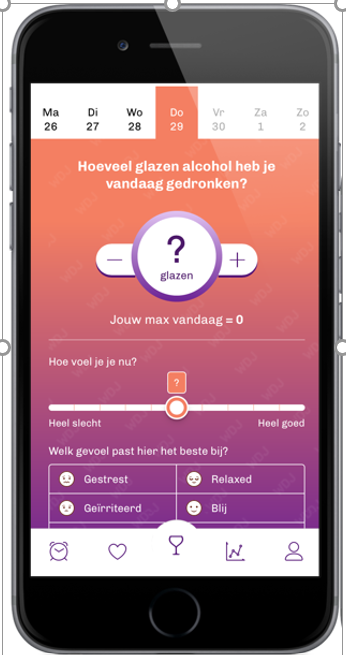


*English translation (c)*

**Get started**

Indicate below how many glasses of alcohol you want to drink in the coming week. Make sure your goal is realistic and achievable for you.

**Reduction of 9 glasses**

You want to cut down your alcohol consumption by 9 glasses this week. What do you think – is this a realistic goal for you? Yes / No

*English translation (d)*

How many glasses of alcohol have you had today?

- ? glasses +

Your max today = 0

How do you feel right now? Very bad --- Very good

Which feeling fits best here: Stressed, Relaxed, Irritated, Happy

(e) Positive reinforcement with (f) Motivational exercise ‘facts and fables quiz’

regard to goal achievement


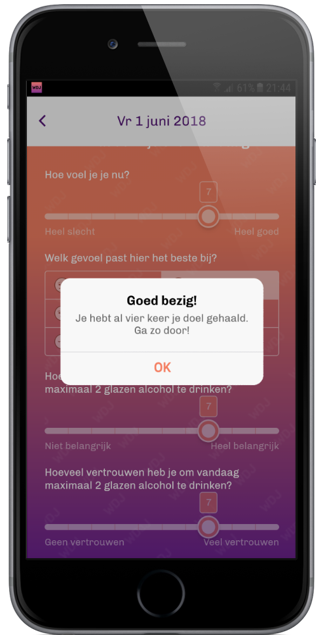

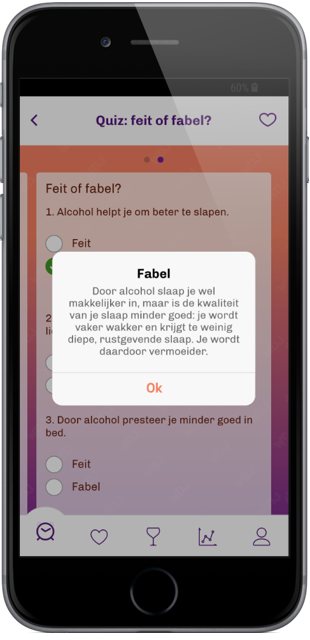


*English translation (e)*

**Well done!**

You have already reached your goal 4 times. Keep it up!

*English translation (f)*

Fact or myth?
1. Alcohol helps you sleep better.

**Myth**

Alcohol may help you fall asleep more easily, but it reduces the quality of your sleep. You wake up more often and get less deep, restorative sleep, which leaves you feeling more tired.

(g) Planning exercise ‘ask a buddy for support’


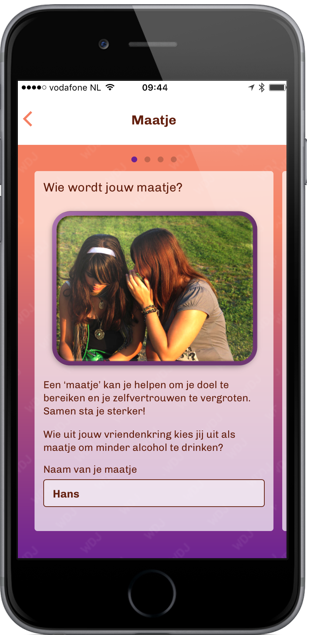


*English translation (g)*

**Buddy**Who will be your buddy?
A buddy can help you reach your goal and boost your confidence. Together, you are stronger!

Who in your circle of friends will you choose as your buddy to help you drink less alcohol?

Name of your buddy:

(h) First menu of WDYD, in which participants could see when the next session was scheduled, and choose for small exercises or movies readily available in between sessions.


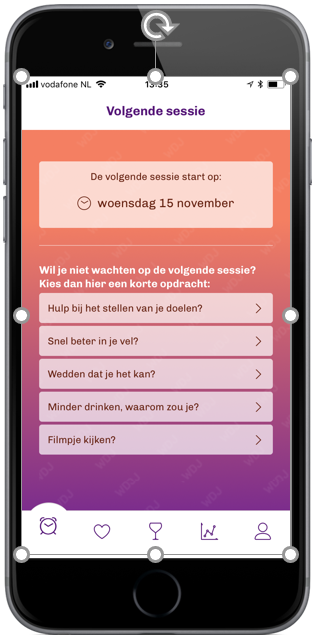


*English translation (h)*

**Next session**

The following sessions starts on: Wednesday 15 November

Do not feel like waiting for the next session? Choose a quick exercise below:

- Help with setting your goals
- Feel better fast
- Bet you can do it!
- Why drink less?
- Watch a short video
