# Supplementary material for: Effects, Acceptability, and Use of a Dynamically Tailored Mobile What Do You Drink Intervention to Reduce Excessive Drinking Among Adolescents and Young Adults in the Netherlands: Randomized Controlled Trial
Source: JMIR Mhealth Uhealth. 2026 May 26;14:e68468. doi: 10.2196/68468 (PMC13211942; doi:10.2196/68468)
Supplement: Multimedia Appendix 3 — Recruitment information. [file mhealth-v14-e68468-s003.docx]

1. Recruitment information of WDYD within the online lifestylemonitor Testjeleefstijl


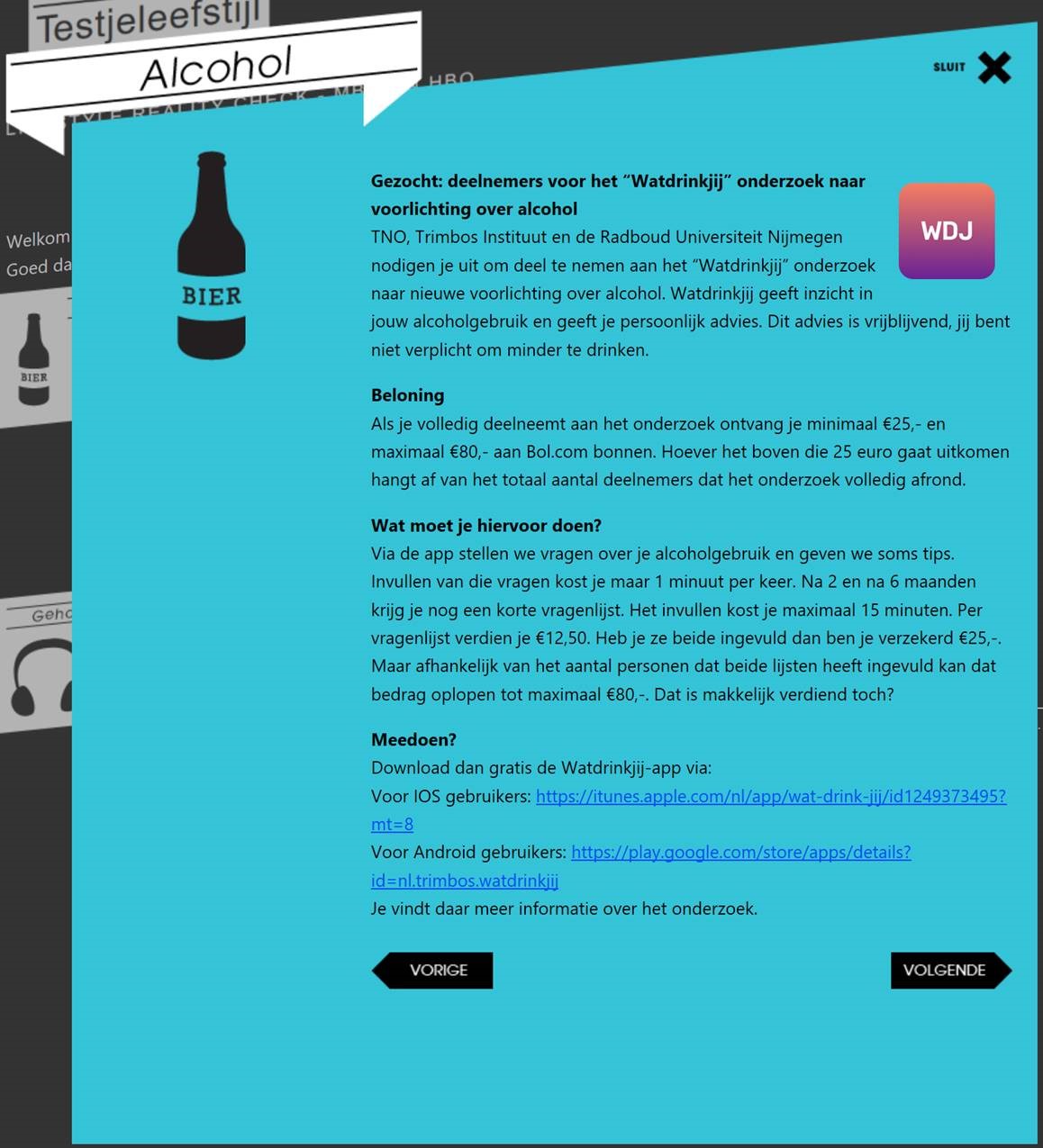


*English translation:*

**Wanted: participants for the “WhatDoYouDrink” study on education about alcohol consumption.**

TNO, Trimbos institute, and Radboud University Nijmegen invite you to participate in the “WhatDoYouDrink” study on new education about alcohol consumption. WhatDoYouDrink provides personal information about alcohol consumption and personalized advice. This advice is without obligation, you are not obliged to reduce your alcohol consumption.

**Reward**

When you complete participation to the study, you will receive a minimum of 25 euro and maximal 80 euro in bol.com gift vouchers. How much you will receive above 25 euro depends on the total amount of participants that complete the study.

**What do you have to do?**

We will ask questions about your alcohol consumption via the app and provide some tips. Answering these questions costs approximately 1 minute per time. After 2 and 6 months, you will receive a brief survey. Answering these surveys costs maximal 15 minutes. Per survey you will earn 12,50 euro. If you have completed both surveys, you will receive 25 euro. Depending on the amount of participants that complete both surveys, the reward can increase up to maximal 80 euro. That is easy money, right?

**Interested?**

Download the WhatDoYouDrink app via:

- For IOS users: [App store link]
- For Android users: [Google Play link]

You will receive more information about the study in the app.

1. Examples of recruitment information of WDYD via social media adds


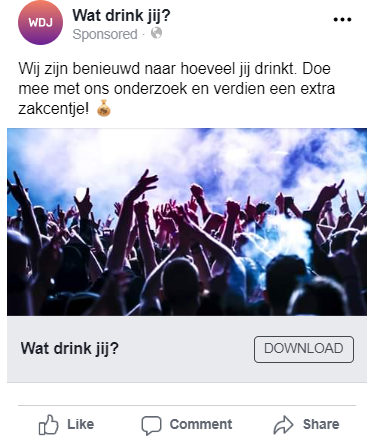

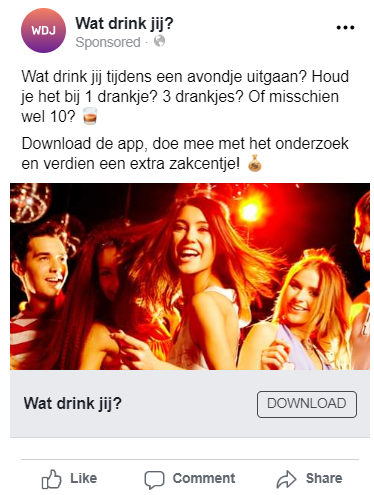


*English translation left add:*

**What do you drink?**

We are curious about how much you drink. Take part in our study and earn a little extra cash!

*English translation right add:*

**What do you drink?**

What do you drink on a night out? Do you stick to just one drink? Three? Or maybe even ten?

Download the app, take part in the study, and earn a little extra cash!
