# Supplementary material for: Effects, Acceptability, and Use of a Dynamically Tailored Mobile What Do You Drink Intervention to Reduce Excessive Drinking Among Adolescents and Young Adults in the Netherlands: Randomized Controlled Trial
Source: JMIR Mhealth Uhealth. 2026 May 26;14:e68468. doi: 10.2196/68468 (PMC13211942; doi:10.2196/68468)
Supplement: Checklist 1 [file mhealth-v14-e68468-s004.pdf]

CONSORT-EHEALTH (V 1.6.1) - Submission/Publication Form

The CONSORT-EHEALTH checklist is intended for authors of randomized trials evaluating web-based and Internet-based applications/interventions, including mobile interventions, electronic games (incl multiplayer games), social media, certain telehealth applications, and other interactive and/or networked electronic applications. Some of the items (e.g. all subitems under item 5 - description of the intervention) may also be applicable for other study designs.

The goal of the CONSORT EHEALTH checklist and guideline is to be
a) a guide for reporting for authors of RCTs,
b) to form a basis for appraisal of an ehealth trial (in terms of validity)

CONSORT-EHEALTH items/subitems are MANDATORY reporting items for studies published in the Journal of Medical Internet Research and other journals / scientific societies endorsing the checklist.

Items numbered 1., 2., 3., 4a., 4b etc are original CONSORT or CONSORT-NPT (non-pharmacologic treatment) items.
Items with Roman numerals (i., ii, iii, iv etc.) are CONSORT-EHEALTH extensions/clarifications.

As the CONSORT-EHEALTH checklist is still considered in a formative stage, we would ask that you also RATE ON A SCALE OF 1-5 how important/useful you feel each item is FOR THE PURPOSE OF THE CHECKLIST and reporting guideline (optional).

Mandatory reporting items are marked with a red *.
In the textboxes, either copy & paste the relevant sections from your manuscript into this form - please include any quotes from your manuscript in QUOTATION MARKS,
or answer directly by providing additional information not in the manuscript, or elaborating on why the item was not relevant for this study.

YOUR ANSWERS WILL BE PUBLISHED AS A SUPPLEMENTARY FILE TO YOUR PUBLICATION IN JMIR AND ARE CONSIDERED PART OF YOUR PUBLICATION (IF ACCEPTED).
Please fill in these questions diligently. Information will not be copyedited, so please use proper spelling and grammar, use correct capitalization, and avoid abbreviations.

DO NOT FORGET TO SAVE AS PDF _AND_ CLICK THE SUBMIT BUTTON SO YOUR ANSWERS ARE IN OUR DATABASE !!!

Citation Suggestion (if you append the pdf as Appendix we suggest to cite this paper in the caption):
Eysenbach G, CONSORT-EHEALTH Group
CONSORT-EHEALTH: Improving and Standardizing Evaluation Reports of Web-based and Mobile Health Interventions
J Med Internet Res 2011;13(4):e126

URL: http://www.jmir.org/2011/4/e126/
doi: 10.2196/jmir.1923
PMID: 22209829

hildevankeulen@gmail.com Ander account
Niet gedeeld
Concept opgeslagen

* Verplichte vraag

Your name *
First Last

Hilde van Keulen

Primary Affiliation (short), City, Country *
University of Toronto, Toronto, Canada

Netherlands Organisation for Applied Scientifi[illegible]

Your e-mail address *
abc@gmail.com

hilde.vankeulen@tno.nl

Title of your manuscript *
Provide the (draft) title of your manuscript.

"Effects, Acceptability and Use of a Dynamically Tailored Mobile What Do You Drink Intervention to Reduce Excessive Drinking among Adolescents and Young Adults in the Netherlands: Randomized Controlled Trial"

Name of your App/Software/Intervention *

If there is a short and a long/alternate name, write the short name first and add the long name in brackets.

Wat drink jij?

Evaluated Version (if any)

e.g. "V1", "Release 2017-03-01", "Version 2.0.27913"

V2.0.1 12 September 2018

Language(s) *

What language is the intervention/app in? If multiple languages are available, separate by comma (e.g. "English, French")

Dutch

URL of your Intervention Website or App

e.g. a direct link to the mobile app on app in appstore (itunes, Google Play), or URL of the website. If the intervention is a DVD or hardware, you can also link to an Amazon page.

Jouw antwoord

URL of an image/screenshot (optional)

Jouw antwoord

Accessibility *

Can an enduser access the intervention presently?

- ( ) access is free and open
- ( ) access only for special usergroups, not open
- ( ) access is open to everyone, but requires payment/subscription/in-app purchases
- (x) app/intervention no longer accessible
- ( ) Anders:

Primary Medical Indication/Disease/Condition *

e.g. "Stress", "Diabetes", or define the target group in brackets after the condition, e.g. "Autism (Parents of children with)", "Alzheimers (Informal Caregivers of)"

Excessive alcohol consumption

Primary Outcomes measured in trial *

comma-separated list of primary outcomes reported in the trial

excessive drinking, binge drinking, weekly alco

Secondary/other outcomes

Are there any other outcomes the intervention is expected to affect?

Jouw antwoord

Recommended "Dose" *

What do the instructions for users say on how often the app should be used?

- ( ) Approximately Daily
- (x) Approximately Weekly
- ( ) Approximately Monthly
- ( ) Approximately Yearly
- ( ) "as needed"
- ( ) Anders:

Approx. Percentage of Users (starters) still using the app as recommended after 3 months *

- ( ) unknown / not evaluated
- (x) 0-10%
- ( ) 11-20%
- ( ) 21-30%
- ( ) 31-40%
- ( ) 41-50%
- ( ) 51-60%
- ( ) 61-70%
- ( ) 71%-80%
- ( ) 81-90%
- ( ) 91-100%
- ( ) Anders:

Overall, was the app/intervention effective? *

- ○ yes: all primary outcomes were significantly better in intervention group vs control
- ◉ partly: SOME primary outcomes were significantly better in intervention group vs control
- ○ no statistically significant difference between control and intervention
- ○ potentially harmful: control was significantly better than intervention in one or more outcomes
- ○ inconclusive: more research is needed
- ○ Anders:

Article Preparation Status/Stage *

At which stage in your article preparation are you currently (at the time you fill in this form)

- ○ not submitted yet - in early draft status
- ○ not submitted yet - in late draft status, just before submission
- ○ submitted to a journal but not reviewed yet
- ◉ submitted to a journal and after receiving initial reviewer comments
- ○ submitted to a journal and accepted, but not published yet
- ○ published
- ○ Anders:

Journal *

If you already know where you will submit this paper (or if it is already submitted), please provide the journal name (if it is not JMIR, provide the journal name under "other")

- ○ not submitted yet / unclear where I will submit this
- ○ Journal of Medical Internet Research (JMIR)
- ◉ JMIR mHealth and UHealth
- ○ JMIR Serious Games
- ○ JMIR Mental Health
- ○ JMIR Public Health
- ○ JMIR Formative Research
- ○ Other JMIR sister journal
- ○ Anders:

Is this a full powered effectiveness trial or a pilot/feasibility trial? *

- ○ Pilot/feasibility
- ◉ Fully powered

Manuscript tracking number *

If this is a JMIR submission, please provide the manuscript tracking number under "other" (The ms tracking number can be found in the submission acknowledgement email, or when you login as author in JMIR. If the paper is already published in JMIR, then the ms tracking number is the four-digit number at the end of the DOI, to be found at the bottom of each published article in JMIR)

- ○ no ms number (yet) / not (yet) submitted to / published in JMIR
- ◉ Anders: ms #68468

TITLE AND ABSTRACT

1a) TITLE: Identification as a randomized trial in the title

1a) Does your paper address CONSORT item 1a? *

I.e does the title contain the phrase "Randomized Controlled Trial"? (if not, explain the reason under "other")

- (●) yes
- ( ) Anders:

1a-i) Identify the mode of delivery in the title

Identify the mode of delivery. Preferably use "web-based" and/or "mobile" and/or "electronic game" in the title. Avoid ambiguous terms like "online", "virtual", "interactive". Use "Internet-based" only if Intervention includes non-web-based Internet components (e.g. email), use "computer-based" or "electronic" only if offline products are used. Use "virtual" only in the context of "virtual reality" (3-D worlds). Use "online" only in the context of "online support groups". Complement or substitute product names with broader terms for the class of products (such as "mobile" or "smart phone" instead of "iphone"), especially if the application runs on different platforms.

	1	2	3	4	5	
subitem not at all important	( )	( )	( )	( )	( )	essential

Does your paper address subitem 1a-i? *

Copy and paste relevant sections from manuscript title (include quotes in quotation marks "like this" to indicate direct quotes from your manuscript), or elaborate on this item by providing additional information not in the ms, or briefly explain why the item is not applicable/relevant for your study

"a Dynamically Tailored Mobile What Do You Drink Intervention"

1a-ii) Non-web-based components or important co-interventions in title

Mention non-web-based components or important co-interventions in title, if any (e.g., “with telephone support”).

	1	2	3	4	5	
subitem not at all important	○	○	○	○	○	essential

Does your paper address subitem 1a-ii?

Copy and paste relevant sections from manuscript title (include quotes in quotation marks "like this" to indicate direct quotes from your manuscript), or elaborate on this item by providing additional information not in the ms, or briefly explain why the item is not applicable/relevant for your study

Jouw antwoord

1a-iii) Primary condition or target group in the title

Mention primary condition or target group in the title, if any (e.g., “for children with Type I Diabetes”) Example: A Web-based and Mobile Intervention with Telephone Support for Children with Type I Diabetes: Randomized Controlled Trial

	1	2	3	4	5	
subitem not at all important	○	○	○	○	○	essential

Does your paper address subitem 1a-iii? *

Copy and paste relevant sections from manuscript title (include quotes in quotation marks "like this" to indicate direct quotes from your manuscript), or elaborate on this item by providing additional information not in the ms, or briefly explain why the item is not applicable/relevant for your study

"among Adolescents and Young Adults in the Netherlands"

1b) ABSTRACT: Structured summary of trial design, methods, results, and conclusions

NPT extension: Description of experimental treatment, comparator, care providers, centers, and blinding status.

1b-i) Key features/functionalities/components of the intervention and comparator in the METHODS section of the ABSTRACT

Mention key features/functionalities/components of the intervention and comparator in the abstract. If possible, also mention theories and principles used for designing the site. Keep in mind the needs of systematic reviewers and indexers by including important synonyms. (Note: Only report in the abstract what the main paper is reporting. If this information is missing from the main body of text, consider adding it)

	1	2	3	4	5	
subitem not at all important	○	○	○	○	○	essential

Does your paper address subitem 1b-i? *

Copy and paste relevant sections from the manuscript abstract (include quotes in quotation marks "like this" to indicate direct quotes from your manuscript), or elaborate on this item by providing additional information not in the ms, or briefly explain why the item is not applicable/relevant for your study

"Participants downloaded the standalone WDYD app, and when having given active informed consent were randomized to the intervention or control group. Participants in the intervention group received dynamically tailored feedback sessions on alcohol consumption (weeks 0–5, 7, 9, 13, and 17) and goal-monitoring reminders. Both groups completed an online baseline survey, two follow-up surveys (weeks 9 and 33), and various EMAs (seven daily assessments during weeks 1, 7, 13, 19, 25, 31, and 33)."

1b-ii) Level of human involvement in the METHODS section of the ABSTRACT

Clarify the level of human involvement in the abstract, e.g., use phrases like “fully automated” vs. “therapist/nurse/care provider/physician-assisted” (mention number and expertise of providers involved, if any). (Note: Only report in the abstract what the main paper is reporting. If this information is missing from the main body of text, consider adding it)

	1	2	3	4	5	
subitem not at all important	○	○	○	○	○	essential

Does your paper address subitem 1b-ii?

Copy and paste relevant sections from the manuscript abstract (include quotes in quotation marks "like this" to indicate direct quotes from your manuscript), or elaborate on this item by providing additional information not in the ms, or briefly explain why the item is not applicable/relevant for your study

Jouw antwoord

1b-iii) Open vs. closed, web-based (self-assessment) vs. face-to-face assessments in the METHODS section of the ABSTRACT

Mention how participants were recruited (online vs. offline), e.g., from an open access website or from a clinic or a closed online user group (closed usergroup trial), and clarify if this was a purely web-based trial, or there were face-to-face components (as part of the intervention or for assessment). Clearly say if outcomes were self-assessed through questionnaires (as common in web-based trials). Note: In traditional offline trials, an open trial (open-label trial) is a type of clinical trial in which both the researchers and participants know which treatment is being administered. To avoid confusion, use “blinded” or “unblinded” to indicated the level of blinding instead of “open”, as “open” in web-based trials usually refers to “open access” (i.e. participants can self-enrol). (Note: Only report in the abstract what the main paper is reporting. If this information is missing from the main body of text, consider adding it)

	1	2	3	4	5	
subitem not at all important	○	○	○	○	○	essential

Does your paper address subitem 1b-iii?

Copy and paste relevant sections from the manuscript abstract (include quotes in quotation marks "like this" to indicate direct quotes from your manuscript), or elaborate on this item by providing additional information not in the ms, or briefly explain why the item is not applicable/relevant for your study

Jouw antwoord

1b-iv) RESULTS section in abstract must contain use data

Report number of participants enrolled/assessed in each group, the use/uptake of the intervention (e.g., attrition/adherence metrics, use over time, number of logins etc.), in addition to primary/secondary outcomes. (Note: Only report in the abstract what the main paper is reporting. If this information is missing from the main body of text, consider adding it)

	1	2	3	4	5	
subitem not at all important	○	○	○	○	○	essential

Does your paper address subitem 1b-iv?

Copy and paste relevant sections from the manuscript abstract (include quotes in quotation marks "like this" to indicate direct quotes from your manuscript), or elaborate on this item by providing additional information not in the ms, or briefly explain why the item is not applicable/relevant for your study

Jouw antwoord

1b-v) CONCLUSIONS/DISCUSSION in abstract for negative trials

Conclusions/Discussions in abstract for negative trials: Discuss the primary outcome - if the trial is negative (primary outcome not changed), and the intervention was not used, discuss whether negative results are attributable to lack of uptake and discuss reasons. (Note: Only report in the abstract what the main paper is reporting. If this information is missing from the main body of text, consider adding it)

	1	2	3	4	5	
subitem not at all important	○	○	○	○	○	essential

Does your paper address subitem 1b-v?

Copy and paste relevant sections from the manuscript abstract (include quotes in quotation marks "like this" to indicate direct quotes from your manuscript), or elaborate on this item by providing additional information not in the ms, or briefly explain why the item is not applicable/relevant for your study

Jouw antwoord

INTRODUCTION

2a) In INTRODUCTION: Scientific background and explanation of rationale

2a-i) Problem and the type of system/solution

Describe the problem and the type of system/solution that is object of the study: intended as stand-alone intervention vs. incorporated in broader health care program? Intended for a particular patient population? Goals of the intervention, e.g., being more cost-effective to other interventions, replace or complement other solutions? (Note: Details about the intervention are provided in “Methods” under 5)

	1	2	3	4	5	
subitem not at all important	○	○	○	○	○	essential

Does your paper address subitem 2a-i? *

Copy and paste relevant sections from the manuscript (include quotes in quotation marks "like this" to indicate direct quotes from your manuscript), or elaborate on this item by providing additional information not in the ms, or briefly explain why the item is not applicable/relevant for your study

Problem and population: "Excessive alcohol drinking among adolescents and young adults is a large public health concern in Western countries [1]; it is negatively associated with short term and longer term negative outcomes, with regard to health, academic performances, social relationships and finishing school (e.g., [1]). Specifically among adolescents and young adults, alcohol consumption occurs in groups and is strongly influenced by the group [2-4]. In the Netherlands, various studies show that adolescents and young adults engage in excessive drinking (e.g., [5-7])."
Solution: "Mobile health (mHealth) interventions can be a useful means to reach young people and to prevent them from drinking excessively [10]. mHealth interventions use mobile phones to deliver interventions and improve health, which are generally prevalent among young people (in the Netherlands, 99,1% uses a mobile phone for internet [11]). They are also widely accessible and can safeguard the users' anonymity (e.g., [12]). In addition, mHealth enables effectively combining information and communication technology with behavior change methods [13]. Although mHealth can be useful, usable and effective, currently available alcohol apps generally contain few behavior change strategies [14]. Empirical evidence is lacking for alcohol prevention mHealth interventions among adolescents and young adults specifically [10]. Research emphasized the need for tailoring [15], personalization [16], and intervening in real-life contexts [10] for this target group." and "In this study we aimed to evaluate an evidence-based 17-week dynamically tailored mHealth intervention, 'What Do You Drink' (WDYD; Trial Registration: onderzoekmetmensen.nl Trial 28135 [17, 18]). Dynamic tailored interventions, also called just-in time adaptive Interventions [19-21] are interventions where the information about the individual is used to determine when and how to intervene, that is, feedback and support is provided based on prior assessments of behavioral and psychological factors. In other words, to make these intervention-related decisions, not only static information (e.g., baseline characteristics), but also time-varying information (e.g., mood, response to intervention) is used [20]."
Goals: "The primary objective was to evaluate the effectiveness of WDYD in reducing excessive alcohol consumption among adolescents and young adults (≥16 years) at risk. Secondary objectives were to assess changes in determinants of drinking behavior (intrinsic motivation, self-confidence, and mood) and to examine the acceptability and use of WDYD."

2a-ii) Scientific background, rationale: What is known about the (type of) system

Scientific background, rationale: What is known about the (type of) system that is the object of the study (be sure to discuss the use of similar systems for other conditions/diagnoses, if appropiate), motivation for the study, i.e. what are the reasons for and what is the context for this specific study, from which stakeholder viewpoint is the study performed, potential impact of findings [2]. Briefly justify the choice of the comparator.

	1	2	3	4	5	
subitem not at all important	○	○	○	○	○	essential

Does your paper address subitem 2a-ii? *

Copy and paste relevant sections from the manuscript (include quotes in quotation marks "like this" to indicate direct quotes from your manuscript), or elaborate on this item by providing additional information not in the ms, or briefly explain why the item is not applicable/relevant for your study

"Mobile health (mHealth) interventions can be a useful means to reach young people and to prevent them from drinking excessively [10]. mHealth interventions use mobile phones to deliver interventions and improve health, which are generally prevalent among young people (in the Netherlands, 99,1% uses a mobile phone for internet [11]). They are also widely accessible and can safeguard the users' anonymity (e.g., [12]). In addition, mHealth enables effectively combining information and communication technology with behavior change methods [13]. Although mHealth can be useful, usable and effective, currently available alcohol apps generally contain few behavior change strategies [14]. Empirical evidence is lacking for alcohol prevention mHealth interventions among adolescents and young adults specifically [10]. Research emphasized the need for tailoring [15], personalization [16], and intervening in real-life contexts [10] for this target group."
"In this study we aimed to evaluate an evidence-based 17-week dynamically tailored mHealth intervention, 'What Do You Drink' (WDYD; Trial Registration: onderzoekmetmensen.nl Trial 28135 [17, 18]). Dynamic tailored interventions, also called just-in time adaptive Interventions [19-21] are interventions where the information about the individual is used to determine when and how to intervene, that is, feedback and support is provided based on prior assessments of behavioral and psychological factors. In other words, to make these intervention-related decisions, not only static information (e.g., baseline characteristics), but also time-varying information (e.g., mood, response to intervention) is used [20]. Thus, dynamically tailored interventions are adapted over time to the dynamic changes of individuals, based on their varying needs. A meta-analytic review has shown that dynamic tailored, just-in time adaptive interventions are more effective than non-dynamic, non-just-in time adaptive interventions [19, 21]. Moreover, dynamically tailored interventions should reduce drop-out, a general problem of digital health interventions [10]."
"In addition, WDYD is based on behavior change techniques that have shown to be effective in changing health behavior and reducing alcohol consumption [22-24]. Examples of general methods used in WDYD are goal setting, action planning, behavioral feedback and self-monitoring. In addition, based on daily assessments of behavioral (alcohol consumption) and psychological factors (motivation, self-confidence and mood) users were provided with weekly tailored support based on a diversity of behavioral change techniques (e.g., planning and problem solving, behavior substitution, modeling and Motivational Interviewing techniques [18])."

2b) In INTRODUCTION: Specific objectives or hypotheses

Does your paper address CONSORT subitem 2b? *

Copy and paste relevant sections from the manuscript (include quotes in quotation marks "like this" to indicate direct quotes from your manuscript), or elaborate on this item by providing additional information not in the ms, or briefly explain why the item is not applicable/relevant for your study

"Objectives
The primary objective was to evaluate the effectiveness of WDYD in reducing excessive alcohol consumption among adolescents and young adults (≥16 years) at risk. Secondary objectives were to assess changes in determinants of drinking behavior (intrinsic motivation, self-confidence, and mood) and to examine the acceptability and use of WDYD."

"Hypotheses
We hypothesized that, compared to the control group (no intervention), participants receiving WDYD would:
1. Show greater reductions in excessive alcohol use, binge drinking, and weekly alcohol consumption (primary outcomes).
2. Demonstrate positive changes in determinants of drinking behavior (secondary outcomes).
3. Evaluate the intervention as acceptable and usable, with measurable engagement reflected in app usage logs."

METHODS

3a) Description of trial design (such as parallel, factorial) including allocation ratio

Does your paper address CONSORT subitem 3a? *

Copy and paste relevant sections from the manuscript (include quotes in quotation marks "like this" to indicate direct quotes from your manuscript), or elaborate on this item by providing additional information not in the ms, or briefly explain why the item is not applicable/relevant for your study

Trial design: "This study was a two-arm, parallel-group, online-only randomized controlled trial (RCT) conducted in the Netherlands between September 2018 and October 2019."
"Within the WDYD app, participants were randomly assigned (1:1) to the experimental group (i.e., WDYD intervention) or control group (no intervention), using a pre-programmed algorithm embedded in WDYD (see for the language code: [27])."

3b) Important changes to methods after trial commencement (such as eligibility criteria), with reasons

Does your paper address CONSORT subitem 3b? *

Copy and paste relevant sections from the manuscript (include quotes in quotation marks "like this" to indicate direct quotes from your manuscript), or elaborate on this item by providing additional information not in the ms, or briefly explain why the item is not applicable/relevant for your study

"Participants also had to be 16 years of age or older at the time they signed up. Initially, WDYD was developed as an intervention with a primary focus on students in secondary vocational education who drank excessively (aged 16 to 24). The intervention was developed in co-creation with these students using a user-centered approach [18]. Due to low enrollment in a prior pilot (N = 253; 19.3% of the 1,310 participants needed), recruitment was expanded to all Dutch adolescents and young adults who drank excessively."

3b-i) Bug fixes, Downtimes, Content Changes

Bug fixes, Downtimes, Content Changes: ehealth systems are often dynamic systems. A description of changes to methods therefore also includes important changes made on the intervention or comparator during the trial (e.g., major bug fixes or changes in the functionality or content) (5-iii) and other "unexpected events" that may have influenced study design such as staff changes, system failures/downtimes, etc. [2].

	1	2	3	4	5	
subitem not at all important	○	○	○	○	○	essential

Does your paper address subitem 3b-i?

Copy and paste relevant sections from the manuscript (include quotes in quotation marks "like this" to indicate direct quotes from your manuscript), or elaborate on this item by providing additional information not in the ms, or briefly explain why the item is not applicable/relevant for your study

Jouw antwoord

4a) Eligibility criteria for participants

Does your paper address CONSORT subitem 4a? *

Copy and paste relevant sections from the manuscript (include quotes in quotation marks "like this" to indicate direct quotes from your manuscript), or elaborate on this item by providing additional information not in the ms, or briefly explain why the item is not applicable/relevant for your study

"The eligibility criteria were based on the Dutch Health Council guidelines for excessive drinking [9] and being 16 years or older. Adolescents (aged 16-18) indicating any amount of routine alcohol consumption were considered excessive drinking. For adults, excessive drinking was defined as:

- Men: drinking more than two drinks per day (heavy drinking) or six or more glasses in one day (binge drinking).
- Women: drinking more than 1 glass per day (heavy drinking) or four or more glasses in one day (binge drinking).

Participants also had to be 16 years of age or older at the time they signed up."

4a-i) Computer / Internet literacy

Computer / Internet literacy is often an implicit "de facto" eligibility criterion - this should be explicitly clarified.

	1	2	3	4	5	
subitem not at all important	○	○	○	○	○	essential

Does your paper address subitem 4a-i?

Copy and paste relevant sections from the manuscript (include quotes in quotation marks "like this" to indicate direct quotes from your manuscript), or elaborate on this item by providing additional information not in the ms, or briefly explain why the item is not applicable/relevant for your study

Jouw antwoord

4a-ii) Open vs. closed, web-based vs. face-to-face assessments:

Open vs. closed, web-based vs. face-to-face assessments: Mention how participants were recruited (online vs. offline), e.g., from an open access website or from a clinic, and clarify if this was a purely web-based trial, or there were face-to-face components (as part of the intervention or for assessment), i.e., to what degree got the study team to know the participant. In online-only trials, clarify if participants were quasi-anonymous and whether having multiple identities was possible or whether technical or logistical measures (e.g., cookies, email confirmation, phone calls) were used to detect/prevent these.

	1	2	3	4	5	
subitem not at all important	○	○	○	○	○	essential

Does your paper address subitem 4a-ii? *

Copy and paste relevant sections from the manuscript (include quotes in quotation marks "like this" to indicate direct quotes from your manuscript), or elaborate on this item by providing additional information not in the ms, or briefly explain why the item is not applicable/relevant for your study

Recruitment was online and offline: "Multiple recruitment channels were used:
1) Students in secondary vocational education were recruited via an online lifestyle monitor ('TestYourLifestyle'; Testjeleefstijl in Dutch) specifically designed for schools and students in secondary vocational education [30]. The monitor consists of various lifestyle modules (e.g., physical activity, nutrition behavior, sexual behavior, smoking) including alcohol consumption. Within the monitor, students can create a user account, receive questions within a module followed by, brief tailored normative feedback (i.e., comparison of their behavior to the Dutch guidelines), behavior-related risk information and information about websites or programs related to the lifestyle topic. To recruit students in secondary vocational education for this study, students who drank alcohol excessively according to the monitor received an invitation to participate with a link to further information about the study and the possibility to download the app to subscribe and provide informed consent for participation.
2) Students in secondary vocational education received an invitation via their teachers as part of an educational program about alcohol consumption
3) Via a social media campaign (Instagram, Facebook) targeting people aged 16-24 years in The Netherlands.
4) Via news items and flyers by the addiction prevention and care institute [31]."
Stand-alone app: "Participants could enroll between September 2018 until the end of January 2019 by downloading the stand-alone WDYD app from the iOS App Store [25] or Google Play Store [26]."
Quasi-anonymous participants: "Participants were quasi-anonymous. Registration required an email address, which was linked to a unique participant code and stored separately. The ICT provider managing the app had access to both datasets; researchers only accessed de-identified data. Email addresses were shared with researchers solely for reward distribution and could not be linked to participant codes. Data protection measures were outlined in the terms of use and privacy statement included in the consent process (see Multimedia Appendix 3 part e)."
Measures to multiple registrations: "No cookies or third-party tracking tools were used to prevent multiple registrations, and no additional identity verification measures were implemented."

4a-iii) Information giving during recruitment

Information given during recruitment. Specify how participants were briefed for recruitment and in the informed consent procedures (e.g., publish the informed consent documentation as appendix, see also item X26), as this information may have an effect on user self-selection, user expectation and may also bias results.

	1	2	3	4	5	
subitem not at all important	○	○	○	○	○	essential

Does your paper address subitem 4a-iii?

Copy and paste relevant sections from the manuscript (include quotes in quotation marks "like this" to indicate direct quotes from your manuscript), or elaborate on this item by providing additional information not in the ms, or briefly explain why the item is not applicable/relevant for your study

Jouw antwoord

4b) Settings and locations where the data were collected

Does your paper address CONSORT subitem 4b? *

Copy and paste relevant sections from the manuscript (include quotes in quotation marks "like this" to indicate direct quotes from your manuscript), or elaborate on this item by providing additional information not in the ms, or briefly explain why the item is not applicable/relevant for your study

All data were collected within the app: "Participants were invited to participate in the study via various recruitment strategies (see Methods-Participants section) and invited to download the WDYD app; access was open and free of charge. Participants who downloaded the app, could sign up and provided informed consent in the app. After consent, participants completed a baseline survey measuring sociodemographic variables, primary and secondary outcomes. Participants in the experimental group received tailored feedback sessions. Invitations for follow-up surveys at weeks 9 and 33, with an estimated completion time of respectively 10 and 5 minutes) were sent via push notifications at 19:00, followed by up to three reminders (i.e., at day 1, 4 and 7)."
And "Outcomes were measured via closed online surveys at baseline and 9 and 33 weeks, and via EMAs consisting of brief daily assessments for 7 consecutive days (weeks 1, 7, 13, 19, 25, 31 and 33). All measurements were administered within the WDYD app"

4b-i) Report if outcomes were (self-)assessed through online questionnaires

Clearly report if outcomes were (self-)assessed through online questionnaires (as common in web-based trials) or otherwise.

	1	2	3	4	5	
subitem not at all important	○	○	○	○	○	essential

Does your paper address subitem 4b-i? *

Copy and paste relevant sections from the manuscript (include quotes in quotation marks "like this" to indicate direct quotes from your manuscript), or elaborate on this item by providing additional information not in the ms, or briefly explain why the item is not applicable/relevant for your study

All surveys and diaries were online within the app: "Outcomes were measured via closed online surveys at baseline and 9 and 33 weeks, and via EMAs consisting of brief daily assessments for 7 consecutive days (weeks 1, 7, 13, 19, 25, 31 and 33). All measurements were administered within the WDYD app"

4b-ii) Report how institutional affiliations are displayed

Report how institutional affiliations are displayed to potential participants [on ehealth media], as affiliations with prestigious hospitals or universities may affect volunteer rates, use, and reactions with regards to an intervention.(Not a required item – describe only if this may bias results)

	1	2	3	4	5	
subitem not at all important	○	○	○	○	○	essential

Does your paper address subitem 4b-ii?

Copy and paste relevant sections from the manuscript (include quotes in quotation marks "like this" to indicate direct quotes from your manuscript), or elaborate on this item by providing additional information not in the ms, or briefly explain why the item is not applicable/relevant for your study

Jouw antwoord

5) The interventions for each group with sufficient details to allow replication, including how and when they were actually administered

5-i) Mention names, credential, affiliations of the developers, sponsors, and owners

Mention names, credential, affiliations of the developers, sponsors, and owners [6] (if authors/evaluators are owners or developer of the software, this needs to be declared in a “Conflict of interest” section or mentioned elsewhere in the manuscript).

	1	2	3	4	5	
subitem not at all important	○	○	○	○	○	essential

Does your paper address subitem 5-i?

Copy and paste relevant sections from the manuscript (include quotes in quotation marks "like this" to indicate direct quotes from your manuscript), or elaborate on this item by providing additional information not in the ms, or briefly explain why the item is not applicable/relevant for your study

Jouw antwoord

5-ii) Describe the history/development process

Describe the history/development process of the application and previous formative evaluations (e.g., focus groups, usability testing), as these will have an impact on adoption/use rates and help with interpreting results.

	1	2	3	4	5	
subitem not at all important	○	○	○	○	○	essential

Does your paper address subitem 5-ii?

Copy and paste relevant sections from the manuscript (include quotes in quotation marks "like this" to indicate direct quotes from your manuscript), or elaborate on this item by providing additional information not in the ms, or briefly explain why the item is not applicable/relevant for your study

Jouw antwoord

5-iii) Revisions and updating

Revisions and updating. Clearly mention the date and/or version number of the application/intervention (and comparator, if applicable) evaluated, or describe whether the intervention underwent major changes during the evaluation process, or whether the development and/or content was “frozen” during the trial. Describe dynamic components such as news feeds or changing content which may have an impact on the replicability of the intervention (for unexpected events see item 3b).

	1	2	3	4	5	
subitem not at all important	○	○	○	○	○	essential

Does your paper address subitem 5-iii?

Copy and paste relevant sections from the manuscript (include quotes in quotation marks "like this" to indicate direct quotes from your manuscript), or elaborate on this item by providing additional information not in the ms, or briefly explain why the item is not applicable/relevant for your study

Jouw antwoord

5-iv) Quality assurance methods

Provide information on quality assurance methods to ensure accuracy and quality of information provided [1], if applicable.

	1	2	3	4	5	
subitem not at all important	○	○	○	○	○	essential

Does your paper address subitem 5-iv?

Copy and paste relevant sections from the manuscript (include quotes in quotation marks "like this" to indicate direct quotes from your manuscript), or elaborate on this item by providing additional information not in the ms, or briefly explain why the item is not applicable/relevant for your study

Jouw antwoord

5-v) Ensure replicability by publishing the source code, and/or providing screenshots/screen-capture video, and/or providing flowcharts of the algorithms used

Ensure replicability by publishing the source code, and/or providing screenshots/screen-capture video, and/or providing flowcharts of the algorithms used. Replicability (i.e., other researchers should in principle be able to replicate the study) is a hallmark of scientific reporting.

	1	2	3	4	5	
subitem not at all important	○	○	○	○	○	essential

Does your paper address subitem 5-v?

Copy and paste relevant sections from the manuscript (include quotes in quotation marks "like this" to indicate direct quotes from your manuscript), or elaborate on this item by providing additional information not in the ms, or briefly explain why the item is not applicable/relevant for your study

Jouw antwoord

5-vi) Digital preservation

Digital preservation: Provide the URL of the application, but as the intervention is likely to change or disappear over the course of the years; also make sure the intervention is archived (Internet Archive, webcitation.org, and/or publishing the source code or screenshots/videos alongside the article). As pages behind login screens cannot be archived, consider creating demo pages which are accessible without login.

	1	2	3	4	5	
subitem not at all important	○	○	○	○	○	essential

Does your paper address subitem 5-vi?

Copy and paste relevant sections from the manuscript (include quotes in quotation marks "like this" to indicate direct quotes from your manuscript), or elaborate on this item by providing additional information not in the ms, or briefly explain why the item is not applicable/relevant for your study

Jouw antwoord

5-vii) Access

Access: Describe how participants accessed the application, in what setting/context, if they had to pay (or were paid) or not, whether they had to be a member of specific group. If known, describe how participants obtained “access to the platform and Internet” [1]. To ensure access for editors/reviewers/readers, consider to provide a “backdoor” login account or demo mode for reviewers/readers to explore the application (also important for archiving purposes, see vi).

	1	2	3	4	5	
subitem not at all important	○	○	○	○	○	essential

Does your paper address subitem 5-vii? *

Copy and paste relevant sections from the manuscript (include quotes in quotation marks "like this" to indicate direct quotes from your manuscript), or elaborate on this item by providing additional information not in the ms, or briefly explain why the item is not applicable/relevant for your study

Recruitment: "Multiple recruitment channels were used:
1) Students in secondary vocational education were recruited via an online lifestyle monitor (‘TestYourLifestyle’; Testjeleefstijl in Dutch) specifically designed for schools and students in secondary vocational education [30]. The monitor consists of various lifestyle modules (e.g., physical activity, nutrition behavior, sexual behavior, smoking) including alcohol consumption. Within the monitor, students can create a user account, receive questions within a module followed by, brief tailored normative feedback (i.e., comparison of their behavior to the Dutch guidelines), behavior-related risk information and information about websites or programs related to the lifestyle topic. To recruit students in secondary vocational education for this study, students who drank alcohol excessively according to the monitor received an invitation to participate with a link to further information about the study and the possibility to download the WDYD app to subscribe and provide informed consent for participation.
2) Students in secondary vocational education received an invitation via their teachers as part of an educational program about alcohol consumption
3) Via a social media campaign (Instagram, Facebook) targeting people aged 16-24 years in The Netherlands.
4) Via news items and flyers by the addiction prevention and care institute [31]."

Open and free access: "Participants were invited to participate in the study via various recruitment strategies (see Methods-Participants section) and invited to download the WDYD app; access was open and free of charge."

5-viii) Mode of delivery, features/functionalities/components of the intervention and comparator, and the theoretical framework

Describe mode of delivery, features/functionalities/components of the intervention and comparator, and the theoretical framework [6] used to design them (instructional strategy [1], behaviour change techniques, persuasive features, etc., see e.g., [7, 8] for terminology). This includes an in-depth description of the content (including where it is coming from and who developed it) [1],” whether [and how] it is tailored to individual circumstances and allows users to track their progress and receive feedback” [6]. This also includes a description of communication delivery channels and – if computer-mediated communication is a component – whether communication was synchronous or asynchronous [6]. It also includes information on presentation strategies [1], including page design principles, average amount of text on pages, presence of hyperlinks to other resources, etc. [1].

	1	2	3	4	5	
subitem not at all important	◯	◯	◯	◯	◯	essential

Does your paper address subitem 5-viii? *

Copy and paste relevant sections from the manuscript (include quotes in quotation marks "like this" to indicate direct quotes from your manuscript), or elaborate on this item by providing additional information not in the ms, or briefly explain why the item is not applicable/relevant for your study

Mode of delivery, components of the intervention, theoretical framework: "WDYD was developed systematically using the Intervention Mapping planning protocol [32] and an iterative, user-centered design process [33]. The development is described elsewhere [18]. WDYD (version 2.0.0) delivered a 17-week dynamically tailored mHealth program.
Content and access to the intervention - WDYD offered a total of 39 different exercises and 22 videos targeting five key goals: 1) motivation, 2) self-confidence, 3) mood, 4) planning, and 5) relapse prevention, using evidence-based techniques (e.g., Motivational Interviewing [34], behavioral modeling [35], goal setting [36], self-monitoring [37], implementation intentions [38], cognitive-behavior therapy [39], provision of rewards [40]) and engagement strategies [22, 23, 41]. In addition, participants could monitor their goal progress in WDYD, in which historic daily alcohol intake was registered as well as whether they had reached their self-set goal. Screenshots of the intervention are shown in Multimedia Appendix 2.
Exercises were tailored based on EMA data. Participants who set a goal were asked to daily monitor their alcohol drinking behavior, mood, motivation and self-confidence. They received push notifications to remind them to provide a daily report. The intervention group received tailored weekly sessions and reminders; This started with six weekly sessions (weeks 0–5), after which the frequency of sessions reduced to two biweekly sessions (weeks 7, 9), and two monthly sessions (weeks 13, 17). See Figure 1 for an overview of the intervention content and timing."
Computer-mediated communication was dynamic and real-time, that is synchronous.
Comparator/control group: "The control group completed surveys (baseline, weeks 9 and 33) and EMAs only (seven daily assessments during weeks 1, 7, 13, 19, 25, 31, and 33), without receiving any feedback or support. They reported their day-to-day alcohol consumption and mood in the EMAs. Motivation and self-confidence to drink less were only asked on the first day of each EMA week.
The control group was also invited to fill in the online baseline survey, and the first and second online follow-up surveys (weeks 9 and 33)."

5-ix) Describe use parameters

Describe use parameters (e.g., intended "doses" and optimal timing for use). Clarify what instructions or recommendations were given to the user, e.g., regarding timing, frequency, heaviness of use, if any, or was the intervention used ad libitum.

	1	2	3	4	5	
subitem not at all important	○	○	○	○	○	essential

Does your paper address subitem 5-ix?

Copy and paste relevant sections from the manuscript (include quotes in quotation marks "like this" to indicate direct quotes from your manuscript), or elaborate on this item by providing additional information not in the ms, or briefly explain why the item is not applicable/relevant for your study

Jouw antwoord

5-x) Clarify the level of human involvement

Clarify the level of human involvement (care providers or health professionals, also technical assistance) in the e-intervention or as co-intervention (detail number and expertise of professionals involved, if any, as well as “type of assistance offered, the timing and frequency of the support, how it is initiated, and the medium by which the assistance is delivered”. It may be necessary to distinguish between the level of human involvement required for the trial, and the level of human involvement required for a routine application outside of a RCT setting (discuss under item 21 – generalizability).

	1	2	3	4	5	
subitem not at all important	○	○	○	○	○	essential

Does your paper address subitem 5-x?

Copy and paste relevant sections from the manuscript (include quotes in quotation marks "like this" to indicate direct quotes from your manuscript), or elaborate on this item by providing additional information not in the ms, or briefly explain why the item is not applicable/relevant for your study

Jouw antwoord

5-xi) Report any prompts/reminders used

Report any prompts/reminders used: Clarify if there were prompts (letters, emails, phone calls, SMS) to use the application, what triggered them, frequency etc. It may be necessary to distinguish between the level of prompts/reminders required for the trial, and the level of prompts/reminders for a routine application outside of a RCT setting (discuss under item 21 – generalizability).

	1	2	3	4	5	
subitem not at all important	○	○	○	○	○	essential

Does your paper address subitem 5-xi? *

Copy and paste relevant sections from the manuscript (include quotes in quotation marks "like this" to indicate direct quotes from your manuscript), or elaborate on this item by providing additional information not in the ms, or briefly explain why the item is not applicable/relevant for your study

Reminders/prompts for the trail (Intervention and control group measurements): "Surveys and EMAs were administered via the WDYD app, with push notifications as reminders."

"After consent, participants completed a baseline survey measuring sociodemographic variables, primary and secondary outcomes. Participants in the experimental group received tailored feedback sessions. Invitations for follow-up surveys at weeks 9 and 33, with an estimated completion time of respectively 10 and 5 minutes) were sent via push notifications at 19:00, followed by up to three reminders (i.e., at day 1, 4 and 7)."

Reminders/prompts for the intervention (intervention group only): "Participants who set a goal were asked to daily monitor their alcohol drinking behavior, mood, motivation and self-confidence. They received push notifications to remind them to provide a daily report."

5-xii) Describe any co-interventions (incl. training/support)

Describe any co-interventions (incl. training/support): Clearly state any interventions that are provided in addition to the targeted eHealth intervention, as ehealth intervention may not be designed as stand-alone intervention. This includes training sessions and support [1]. It may be necessary to distinguish between the level of training required for the trial, and the level of training for a routine application outside of a RCT setting (discuss under item 21 – generalizability.

	1	2	3	4	5	
subitem not at all important	○	○	○	○	○	essential

Does your paper address subitem 5-xii? *

Copy and paste relevant sections from the manuscript (include quotes in quotation marks "like this" to indicate direct quotes from your manuscript), or elaborate on this item by providing additional information not in the ms, or briefly explain why the item is not applicable/relevant for your study

"Participants could enroll between September 2018 until the end of January 2019 by downloading the stand-alone WDYD app from the iOS App Store [25] or Google Play Store [26]."

6a) Completely defined pre-specified primary and secondary outcome measures, including how and when they were assessed

Does your paper address CONSORT subitem 6a? *

Copy and paste relevant sections from the manuscript (include quotes in quotation marks "like this" to indicate direct quotes from your manuscript), or elaborate on this item by providing additional information not in the ms, or briefly explain why the item is not applicable/relevant for your study

"Outcome variables
Outcomes were measured via closed online surveys at baseline and 9 and 33 weeks, and via EMAs consisting of brief daily assessments for 7 consecutive days (weeks 1, 7, 13, 19, 25, 31 and 33). All measurements were administered within the WDYD app and pretested for usability and technical functionality.

Primary outcomes
Weekly alcohol consumption was measured as the number of standard drinks per week, assessed at baseline using daily intake questions [42, 43] and during EMA bursts using the question: “How many glasses of alcoholic drinks did you drink yesterday?” (range: 0–100) [42]. Weekly alcohol intake was calculated based on a minimum of four EMAs in a given week.
Binge drinking was defined as drinking more than 3 (women) or more than 5 (men) standard alcohol units on at least one day of the week; the day with the highest reported number of glasses consumed was used to determine whether binge drinking occurred.
Excessive drinking was defined as binge drinking (i.e., drinking four (women) or six (men) or more glasses in one day) and/or heavy drinking (i.e., drinking any amount of alcohol when under the legal drinking age of 18, and for adults to drink a maximum of one (women) or two (men) glasses of standard alcohol units on an average day [9]). Heavy drinking was determined by comparing the extrapolated number of glasses of alcoholic drinks in a week (more than 7 glasses for women, and more than 14 glasses for men).

Secondary outcomes.
Intrinsic motivation was measured by the question: “How important is it for you to drink less alcoholic drinks” (0 = not at all important, to 10 = very important) [34].
Self-confidence was assessed with the question: “How confident are you that you can drink less alcoholic drinks” (0 = not at all confident, to 10 = very confident [43, 44]
Mood was assessed with the question: “How do you feel?” (0 = very bad, to 10 = very good [43, 45, 46]. These were measured at baseline (except for mood), week 1 (except for motivation and confidence) and weeks 7, 13, 19, 25, 31 and 33. For mood, the weekly average was calculated if participants filled out a minimum of four daily assessments. Motivation and self-confidence were measured on the first day of each EMA week. Mood was measured daily in a EMA week, and an average weekly mood index was created based on a minimum of 4 EMA entries.

Sociodemographic variables
Sociodemographic variables were measured at baseline. These variables were age, gender, migrant background, ongoing educational level and highest completed educational level. Both education variables were rescored to low (less than secondary or vocational education), intermediate (secondary through pre-university education) or high (professional or university education). Specifically for students in secondary vocational education, we assessed their qualification level (1-4), school year (1-4) and pathway (practical/vocational training/combination).

Program evaluation and use
Program evaluation - Participants in the intervention group were asked to evaluate WDYD in the first follow-up survey (week 9).
WDYD usability was assessed by 5 items, e.g., “How do you perceive the user-friendliness of WDYD?” on a 10-point scale (e.g. 1 = not at all user-friendly, to 10 = very user-friendly), Information quality was also assessed by 5 items (e.g., “How do you perceive the credibility of the information in WDYD?”, with 10-points scales (e.g. 1 = not at all credible, to 10 = very credible).

Personalization was assessed by 8 items (e.g.., “WDYD took into account my personal preferences”, scales 1 = totally agree, to 5 = totally disagree).
Finally, participants were asked to grade the overall acceptability of WDYD on a scale from 1 (very bad) to 10 (excellent) [47].
Program use - For participants in the intervention group, use of WDYD was logged in the app. Session completion was calculated as the average number of sessions completed across all participants in the intervention group.
Exercises completed was calculated by dividing the number of completed exercises within a given category by the total number of exercises participants had chosen."

6a-i) Online questionnaires: describe if they were validated for online use and apply CHERRIES items to describe how the questionnaires were designed/deployed

If outcomes were obtained through online questionnaires, describe if they were validated for online use and apply CHERRIES items to describe how the questionnaires were designed/deployed [9].

	1	2	3	4	5	
subitem not at all important	○	○	○	○	○	essential

Does your paper address subitem 6a-i?

Copy and paste relevant sections from manuscript text

Jouw antwoord

6a-ii) Describe whether and how “use” (including intensity of use/dosage) was defined/measured/monitored

Describe whether and how “use” (including intensity of use/dosage) was defined/measured/monitored (logins, logfile analysis, etc.). Use/adoption metrics are important process outcomes that should be reported in any ehealth trial.

	1	2	3	4	5	
subitem not at all important	○	○	○	○	○	essential

Does your paper address subitem 6a-ii?

Copy and paste relevant sections from manuscript text

Jouw antwoord

6a-iii) Describe whether, how, and when qualitative feedback from participants was obtained

Describe whether, how, and when qualitative feedback from participants was obtained (e.g., through emails, feedback forms, interviews, focus groups).

	1	2	3	4	5	
subitem not at all important	○	○	○	○	○	essential

Does your paper address subitem 6a-iii?

Copy and paste relevant sections from manuscript text

Jouw antwoord

6b) Any changes to trial outcomes after the trial commenced, with reasons

Does your paper address CONSORT subitem 6b? *

Copy and paste relevant sections from the manuscript (include quotes in quotation marks "like this" to indicate direct quotes from your manuscript), or elaborate on this item by providing additional information not in the ms, or briefly explain why the item is not applicable/relevant for your study

Not applicable, no changes were made to the outcomes after the trial commenced

7a) How sample size was determined

NPT: When applicable, details of whether and how the clustering by care provides or centers was addressed

7a-i) Describe whether and how expected attrition was taken into account when calculating the sample size

Describe whether and how expected attrition was taken into account when calculating the sample size.

	1	2	3	4	5	
subitem not at all important	○	○	○	○	○	essential

Does your paper address subitem 7a-i?

Copy and paste relevant sections from manuscript title (include quotes in quotation marks "like this" to indicate direct quotes from your manuscript), or elaborate on this item by providing additional information not in the ms, or briefly explain why the item is not applicable/relevant for your study

Jouw antwoord

7b) When applicable, explanation of any interim analyses and stopping guidelines

Does your paper address CONSORT subitem 7b? *

Copy and paste relevant sections from the manuscript (include quotes in quotation marks "like this" to indicate direct quotes from your manuscript), or elaborate on this item by providing additional information not in the ms, or briefly explain why the item is not applicable/relevant for your study

No interim analyses or stopping guideline, because the analyses were fixed/determined, see: "Data were analyzed using R [49]. Logistic regression analyses were also used to analyze the drop-out probability at the start (i.e., within the first week), and throughout the study (i.e., within 33 weeks), with primary, secondary and socio-demographic variables at baseline as predictors. Descriptive statistics (%, N, mean, standard deviation, median, interquartile range) were used to analyze baseline characteristics of the sample (i.e., socio-demographic and outcome variables) and to evaluate usability and acceptability of WDYD. A logistic regression was used to evaluate the randomization by analyzing the probability of intervention group assignment using the primary, secondary and socio-demographic variables observed at baseline. Baseline logistic regression analyses were used to examine differences in drinking behavior at baseline. Baseline differences between the intervention and control group or in drinking behavior were accounted for by the random intercept in multilevel models for the effect analyses.
Effects of WDYD on primary and secondary outcomes were analyzed using multilevel generalized linear regression models using the R package ‘lme4’ [50]. A multilevel logistic regression model was used for dichotomous outcomes (i.e., excessive drinking and binge drinking), a multilevel negative binomial regression model for count outcomes (i.e., number of glasses), and a linear multilevel regression model for continuous outcomes (i.e. mood, motivation and self-confidence). In these models, time (categorical: a dummy for every diary week with baseline as reference category), group (intervention vs. control), and the interaction between time and group were used as predictors. In addition, a random intercept was included in each model to account for participant-level variability.
Missing values were imputed using multiple imputation with multilevel predictive mean matching using 100 imputations and 25 iterations, using the R package ‘mice’ [51]. The imputation model contained the primary and secondary outcomes (alcohol consumption, mood, intrinsic motivation, self-confidence) from the daily assessment data, variables that were additionally related to dropout (see results: besides alcohol consumption these were group, enrolled in secondary vocational education), time, number of sessions attended (0-10), gender and intention to reduce excessive drinking at baseline and week 9. We performed additional complete case analyses on both primary and secondary outcomes to check for differences between analyses based upon multiple imputation and those based on complete cases only."

8a) Method used to generate the random allocation sequence

NPT: When applicable, how care providers were allocated to each trial group

Does your paper address CONSORT subitem 8a? *

Copy and paste relevant sections from the manuscript (include quotes in quotation marks "like this" to indicate direct quotes from your manuscript), or elaborate on this item by providing additional information not in the ms, or briefly explain why the item is not applicable/relevant for your study

"Within the WDYD app, participants were randomly assigned (1:1) to the experimental group (i.e., WDYD intervention) or control group (no intervention), using a pre-programmed algorithm embedded in WDYD (see for the language code: [27])."

8b) Type of randomisation; details of any restriction (such as blocking and block size)

Does your paper address CONSORT subitem 8b? *

Copy and paste relevant sections from the manuscript (include quotes in quotation marks "like this" to indicate direct quotes from your manuscript), or elaborate on this item by providing additional information not in the ms, or briefly explain why the item is not applicable/relevant for your study

"Within the WDYD app, participants were randomly assigned (1:1) to the experimental group (i.e., WDYD intervention) or control group (no intervention), using a pre-programmed algorithm embedded in WDYD (see for the language code: [27])."

9) Mechanism used to implement the random allocation sequence (such as sequentially numbered containers), describing any steps taken to conceal the sequence until interventions were assigned

Does your paper address CONSORT subitem 9? *

Copy and paste relevant sections from the manuscript (include quotes in quotation marks "like this" to indicate direct quotes from your manuscript), or elaborate on this item by providing additional information not in the ms, or briefly explain why the item is not applicable/relevant for your study

"Within the WDYD app, participants were randomly assigned (1:1) to the experimental group (i.e., WDYD intervention) or control group (no intervention), using a pre-programmed algorithm embedded in WDYD (see for the language code: [27])."

10) Who generated the random allocation sequence, who enrolled participants, and who assigned participants to interventions

Does your paper address CONSORT subitem 10? *

Copy and paste relevant sections from the manuscript (include quotes in quotation marks "like this" to indicate direct quotes from your manuscript), or elaborate on this item by providing additional information not in the ms, or briefly explain why the item is not applicable/relevant for your study

"Within the WDYD app, participants were randomly assigned (1:1) to the experimental group (i.e., WDYD intervention) or control group (no intervention), using a pre-programmed algorithm embedded in WDYD (see for the language code: [27])."

11a) If done, who was blinded after assignment to interventions (for example, participants, care providers, those assessing outcomes) and how

NPT: Whether or not administering co-interventions were blinded to group assignment

11a-i) Specify who was blinded, and who wasn't

Specify who was blinded, and who wasn't. Usually, in web-based trials it is not possible to blind the participants [1, 3] (this should be clearly acknowledged), but it may be possible to blind outcome assessors, those doing data analysis or those administering co-interventions (if any).

	1	2	3	4	5	
subitem not at all important	○	○	○	○	○	essential

Does your paper address subitem 11a-i? *

Copy and paste relevant sections from the manuscript (include quotes in quotation marks "like this" to indicate direct quotes from your manuscript), or elaborate on this item by providing additional information not in the ms, or briefly explain why the item is not applicable/relevant for your study

"Treatment assignment was not blinded; participants were informed about both groups during consent and may have become aware of their group allocation after randomization."

11a-ii) Discuss e.g., whether participants knew which intervention was the "intervention of interest" and which one was the "comparator"

Informed consent procedures (4a-ii) can create biases and certain expectations - discuss e.g., whether participants knew which intervention was the "intervention of interest" and which one was the "comparator".

	1	2	3	4	5	
subitem not at all important	○	○	○	○	○	essential

Does your paper address subitem 11a-ii?

Copy and paste relevant sections from the manuscript (include quotes in quotation marks "like this" to indicate direct quotes from your manuscript), or elaborate on this item by providing additional information not in the ms, or briefly explain why the item is not applicable/relevant for your study

Jouw antwoord

11b) If relevant, description of the similarity of interventions

(this item is usually not relevant for ehealth trials as it refers to similarity of a placebo or sham intervention to a active medication/intervention)

Does your paper address CONSORT subitem 11b? *

Copy and paste relevant sections from the manuscript (include quotes in quotation marks "like this" to indicate direct quotes from your manuscript), or elaborate on this item by providing additional information not in the ms, or briefly explain why the item is not applicable/relevant for your study

Not applicable, the control group received measures only: the surveys and EMA's.

12a) Statistical methods used to compare groups for primary and secondary outcomes

NPT: When applicable, details of whether and how the clustering by care providers or centers was addressed

Does your paper address CONSORT subitem 12a? *

Copy and paste relevant sections from the manuscript (include quotes in quotation marks "like this" to indicate direct quotes from your manuscript), or elaborate on this item by providing additional information not in the ms, or briefly explain why the item is not applicable/relevant for your study

"Effects of WDYD on primary and secondary outcomes were analyzed using multilevel generalized linear regression models using the R package ‘lme4’ [50]. A multilevel logistic regression model was used for dichotomous outcomes (i.e., excessive drinking and binge drinking), a multilevel negative binomial regression model for count outcomes (i.e., number of glasses), and a linear multilevel regression model for continuous outcomes (i.e. mood, motivation and self-confidence). In these models, time (categorical: a dummy for every diary week with baseline as reference category), group (intervention vs. control), and the interaction between time and group were used as predictors. In addition, a random intercept was included in each model to account for participant-level variability."

12a-i) Imputation techniques to deal with attrition / missing values

Imputation techniques to deal with attrition / missing values: Not all participants will use the intervention/comparator as intended and attrition is typically high in ehealth trials. Specify how participants who did not use the application or dropped out from the trial were treated in the statistical analysis (a complete case analysis is strongly discouraged, and simple imputation techniques such as LOCF may also be problematic [4]).

	1	2	3	4	5	
subitem not at all important	○	○	○	○	○	essential

Does your paper address subitem 12a-i? *

Copy and paste relevant sections from the manuscript (include quotes in quotation marks "like this" to indicate direct quotes from your manuscript), or elaborate on this item by providing additional information not in the ms, or briefly explain why the item is not applicable/relevant for your study

"Missing values were imputed using multiple imputation with multilevel predictive mean matching using 100 imputations and 25 iterations, using the R package 'mice' [51]. The imputation model contained the primary and secondary outcomes (alcohol consumption, mood, intrinsic motivation, self-confidence) from the daily assessment data, variables that were additionally related to dropout (see results: besides alcohol consumption these were group, enrolled in secondary vocational education), time, number of sessions attended (0-10), gender and intention to reduce excessive drinking at baseline and week 9. We performed additional complete case analyses on both primary and secondary outcomes to check for differences between analyses based upon multiple imputation and those based on complete cases only."

12b) Methods for additional analyses, such as subgroup analyses and adjusted analyses

Does your paper address CONSORT subitem 12b? *

Copy and paste relevant sections from the manuscript (include quotes in quotation marks "like this" to indicate direct quotes from your manuscript), or elaborate on this item by providing additional information not in the ms, or briefly explain why the item is not applicable/relevant for your study

"Data were analyzed using R [49]. Logistic regression analyses were also used to analyze the drop-out probability at the start (i.e., within the first week), and throughout the study (i.e., within 33 weeks), with primary, secondary and socio-demographic variables at baseline as predictors. Descriptive statistics (%, N, mean, standard deviation, median, interquartile range) were used to analyze baseline characteristics of the sample (i.e., socio-demographic and outcome variables) and to evaluate usability and acceptability of WDYD. A logistic regression was used to evaluate the randomization by analyzing the probability of intervention group assignment using the primary, secondary and socio-demographic variables observed at baseline. Baseline logistic regression analyses were used to examine differences in drinking behavior at baseline. Baseline differences between the intervention and control group or in drinking behavior were accounted for by the random intercept in multilevel models for the effect analyses."

"We did conduct additional moderation analyses, and found no evidence of educational attainment moderating the treatment effect (data not shown)."

X26) REB/IRB Approval and Ethical Considerations [recommended as subheading under "Methods"] (not a CONSORT item)

X26-i) Comment on ethics committee approval

	1	2	3	4	5	
subitem not at all important	○	○	○	○	○	essential

Does your paper address subitem X26-i?

Copy and paste relevant sections from the manuscript (include quotes in quotation marks "like this" to indicate direct quotes from your manuscript), or elaborate on this item by providing additional information not in the ms, or briefly explain why the item is not applicable/relevant for your study

Jouw antwoord

x26-ii) Outline informed consent procedures

Outline informed consent procedures e.g., if consent was obtained offline or online (how? Checkbox, etc.?), and what information was provided (see 4a-ii). See [6] for some items to be included in informed consent documents.

	1	2	3	4	5	
subitem not at all important	○	○	○	○	○	essential

Does your paper address subitem X26-ii?

Copy and paste relevant sections from the manuscript (include quotes in quotation marks "like this" to indicate direct quotes from your manuscript), or elaborate on this item by providing additional information not in the ms, or briefly explain why the item is not applicable/relevant for your study

Jouw antwoord

X26-iii) Safety and security procedures

Safety and security procedures, incl. privacy considerations, and any steps taken to reduce the likelihood or detection of harm (e.g., education and training, availability of a hotline)

	1	2	3	4	5	
subitem not at all important	○	○	○	○	○	essential

Does your paper address subitem X26-iii?

Copy and paste relevant sections from the manuscript (include quotes in quotation marks "like this" to indicate direct quotes from your manuscript), or elaborate on this item by providing additional information not in the ms, or briefly explain why the item is not applicable/relevant for your study

Jouw antwoord

RESULTS

13a) For each group, the numbers of participants who were randomly assigned, received intended treatment, and were analysed for the primary outcome

NPT: The number of care providers or centers performing the intervention in each group and the number of patients treated by each care provider in each center

Does your paper address CONSORT subitem 13a? *

Copy and paste relevant sections from the manuscript (include quotes in quotation marks "like this" to indicate direct quotes from your manuscript), or elaborate on this item by providing additional information not in the ms, or briefly explain why the item is not applicable/relevant for your study

"A total of 6,146 people downloaded the WDYD app between 1 September 2018 and 31 January 2019, and agreed to participate in the study."
"We were unable to determine group allocation of 17 participants due to data loss of group allocation at baseline. The remaining 6,129 participants were randomized into the intervention (n = 3,088) or control group (n = 3,041). After filling in the baseline survey, 1,334 participants did not meet the inclusion criteria and were excluded from further analysis."
"Participants were excluded from all analyses when they did not meet the inclusion criteria (i.e., excessive drinking and >=16 years of age; see participants' section; n = 1,207) or if they did not complete the baseline questionnaire (n = 127). The amount of participants that dropped-out of the study was large, especially in the first diary week (n = 3,028). We excluded participants who dropped-out within the first diary week from main analysis, as not enough information was observed to allow for imputation. In addition, the number of participants that completed the measurements during weeks 31 and 33 was insufficient for reliable imputation. As a result, our imputation model was unable to produce stable imputed outcomes. For this reason we restricted our analysis to data collected up to week 25."
"The final sample for the main analyses consisted of 1,767 participants (nintervention = 720, ncontrol = 1,047). Missing values of these participants were imputed for analyses. Trace plots showed that the imputed datasets had converged, and density and scatterplots between observed and imputed data showed plausible imputed values."

13b) For each group, losses and exclusions after randomisation, together with reasons

Does your paper address CONSORT subitem 13b? (NOTE: Preferably, this is shown in a CONSORT flow diagram) *

Copy and paste relevant sections from the manuscript (include quotes in quotation marks "like this" to indicate direct quotes from your manuscript), or elaborate on this item by providing additional information not in the ms, or briefly explain why the item is not applicable/relevant for your study

"Participants were excluded from all analyses when they did not meet the inclusion criteria (i.e., excessive drinking and >=16 years of age; see participants' section; n = 1,207) or if they did not complete the baseline questionnaire (n = 127). The amount of participants that dropped-out of the study was large, especially in the first diary week (n = 3,028). We excluded participants who dropped-out within the first diary week from main analysis, as not enough information was observed to allow for imputation. In addition, the number of participants that completed the measurements during weeks 31 and 33 was insufficient for reliable imputation. As a result, our imputation model was unable to produce stable imputed outcomes. For this reason we restricted our analysis to data collected up to week 25."

"The final sample for the main analyses consisted of 1,767 participants (nintervention = 720, ncontrol = 1,047). Missing values of these participants were imputed for analyses. Trace plots showed that the imputed datasets had converged, and density and scatterplots between observed and imputed data showed plausible imputed values."

13b-i) Attrition diagram

Strongly recommended: An attrition diagram (e.g., proportion of participants still logging in or using the intervention/comparator in each group plotted over time, similar to a survival curve) or other figures or tables demonstrating usage/dose/engagement.

	1	2	3	4	5	
subitem not at all important	O	O	O	O	O	essential

Does your paper address subitem 13b-i?

Copy and paste relevant sections from the manuscript or cite the figure number if applicable (include quotes in quotation marks "like this" to indicate direct quotes from your manuscript), or elaborate on this item by providing additional information not in the ms, or briefly explain why the item is not applicable/relevant for your study

Jouw antwoord

14a) Dates defining the periods of recruitment and follow-up

Does your paper address CONSORT subitem 14a? *

Copy and paste relevant sections from the manuscript (include quotes in quotation marks "like this" to indicate direct quotes from your manuscript), or elaborate on this item by providing additional information not in the ms, or briefly explain why the item is not applicable/relevant for your study

"This study was a two-arm, parallel-group, online-only randomized controlled trial (RCT) conducted in the Netherlands between September 2018 and October 2019. "
"Participants could enroll between September 2018 until the end of January 2019 by downloading the stand-alone WDYD app from the iOS App Store [25] or Google Play Store [26]."
"The trial lasted 33 weeks and consisted of an online survey at baseline, followed by additional online surveys at 9 and 33 weeks. To account for the fluctuating nature of alcohol drinking and contextual influences [28, 29], participants were also asked to report their alcohol intake each day using Ecological Momentary Assessment (EMA) on seven weeks (weeks 1, 7, 13, 19, 25, 31 and 33), each consisting of 7 consecutive daily assessments."
"WDYD (version 2.0.0) delivered a 17-week dynamically tailored mHealth program."

14a-i) Indicate if critical “secular events” fell into the study period

Indicate if critical “secular events” fell into the study period, e.g., significant changes in Internet resources available or “changes in computer hardware or Internet delivery resources”

	1	2	3	4	5	
subitem not at all important	O	O	O	O	O	essential

Does your paper address subitem 14a-i?

Copy and paste relevant sections from the manuscript (include quotes in quotation marks "like this" to indicate direct quotes from your manuscript), or elaborate on this item by providing additional information not in the ms, or briefly explain why the item is not applicable/relevant for your study

Jouw antwoord

14b) Why the trial ended or was stopped (early)

Does your paper address CONSORT subitem 14b? *

Copy and paste relevant sections from the manuscript (include quotes in quotation marks "like this" to indicate direct quotes from your manuscript), or elaborate on this item by providing additional information not in the ms, or briefly explain why the item is not applicable/relevant for your study

The trial ended because we had enough participants according to the power calculation:
"The sample size was based on a priori power calculation for detecting a reduction in excessive drinking in the intervention group compared to the control group. We anticipated a small effect size (Cohen’s d = 0.20) based on a previous study of an earlier version of WDYD [29] and a just-in-time adaptive alcohol intervention [41]. In the previous study, the EMA procedure, including rewarding of participants, resulted in a 20% dropout [29]. When anticipating a conservative dropout rate of 40% [48], a small effect size (ES (d) = 0.20), a power of .80 and a 2-sided alpha of .05, 655 participants were required per group at baseline."
"The final sample for the main analyses consisted of 1,767 participants (nintervention = 720, ncontrol = 1,047). "

15) A table showing baseline demographic and clinical characteristics for each group

NPT: When applicable, a description of care providers (case volume, qualification, expertise, etc.) and centers (volume) in each group

Does your paper address CONSORT subitem 15? *

Copy and paste relevant sections from the manuscript (include quotes in quotation marks "like this" to indicate direct quotes from your manuscript), or elaborate on this item by providing additional information not in the ms, or briefly explain why the item is not applicable/relevant for your study

See Table 1 in the manuscript: "Baseline characteristics of participants are depicted in Table 1. There were no significant differences between participants in the intervention and control group regarding sociodemographic characteristics, primary and secondary outcomes (data not shown). Small differences were found in drinking behavior by recruitment strategy. Participants reporting higher weekly alcohol consumption were more likely recruited via social media ($exp(\beta) = 1.01$, $P < .001$) and less likely via teachers ($exp(\beta) = 0.95$, $P < .001$) or the lifestyle monitor ($exp(\beta) = 0.92$, $P < .001$). No other significant baseline differences were observed on drinking behavior, and no interaction effects of recruitment strategy with group assignment were found. Slightly more than half of the participants were male (52.5%), 72.4% were aged 18 to 24 years, most of them attended or had completed a high educational level (56.3%) and 34.6% attended secondary vocational education."

15-i) Report demographics associated with digital divide issues

In ehealth trials it is particularly important to report demographics associated with digital divide issues, such as age, education, gender, social-economic status, computer/Internet/ehealth literacy of the participants, if known.

	1	2	3	4	5	
subitem not at all important	○	○	○	○	○	essential

Does your paper address subitem 15-i? *

Copy and paste relevant sections from the manuscript (include quotes in quotation marks "like this" to indicate direct quotes from your manuscript), or elaborate on this item by providing additional information not in the ms, or briefly explain why the item is not applicable/relevant for your study

See Table 1 in the manuscript: "Baseline characteristics of participants are depicted in Table 1. There were no significant differences between participants in the intervention and control group regarding sociodemographic characteristics, primary and secondary outcomes (data not shown). Small differences were found in drinking behavior by recruitment strategy. Participants reporting higher weekly alcohol consumption were more likely recruited via social media (exp(β) = 1.01, P < .001) and less likely via teachers (exp(β) = 0.95, P < .001) or the lifestyle monitor (exp(β) = 0.92, P < .001). No other significant baseline differences were observed on drinking behavior, and no interaction effects of recruitment strategy with group assignment were found. Slightly more than half of the participants were male (52.5%), 72.4% were aged 18 to 24 years, most of them attended or had completed a high educational level (56.3%) and 34.6% attended secondary vocational education."
Social-economic status and eHealth literacy were not measured.

16) For each group, number of participants (denominator) included in each analysis and whether the analysis was by original assigned groups

16-i) Report multiple "denominators" and provide definitions

Report multiple "denominators" and provide definitions: Report N's (and effect sizes) "across a range of study participation [and use] thresholds" [1], e.g., N exposed, N consented, N used more than x times, N used more than y weeks, N participants "used" the intervention/comparator at specific pre-defined time points of interest (in absolute and relative numbers per group). Always clearly define "use" of the intervention.

	1	2	3	4	5	
subitem not at all important	○	○	○	○	○	essential

Does your paper address subitem 16-i? *

Copy and paste relevant sections from the manuscript (include quotes in quotation marks "like this" to indicate direct quotes from your manuscript), or elaborate on this item by providing additional information not in the ms, or briefly explain why the item is not applicable/relevant for your study

See Figure 2 for the flow of participants in the WDYD study.

"The remaining 6,129 participants were randomized into the intervention (n = 3,088) or control group (n = 3,041). After filling in the baseline survey, 1,334 participants did not meet the inclusion criteria and were excluded from further analysis. Of the participants who met the inclusion criteria, 36.9% (n = 1,767) completed the first week of daily assessments, 10.8% (n = 516) completed the first follow-up survey (week 9), and 4.0 % (n = 192) completed the second follow-up survey (week 33). "
"Participants were excluded from all analyses when they did not meet the inclusion criteria (i.e., excessive drinking and >=16 years of age; see participants' section; n = 1,207) or if they did not complete the baseline questionnaire (n = 127). The amount of participants that dropped-out of the study was large, especially in the first diary week (n = 3,028). We excluded participants who dropped-out within the first diary week from main analysis, as not enough information was observed to allow for imputation. In addition, the number of participants that completed the measurements during weeks 31 and 33 was insufficient for reliable imputation. As a result, our imputation model was unable to produce stable imputed outcomes. For this reason we restricted our analysis to data collected up to week 25.
The final sample for the main analyses consisted of 1,767 participants (nintervention = 720, ncontrol = 1,047)."

16-ii) Primary analysis should be intent-to-treat

Primary analysis should be intent-to-treat, secondary analyses could include comparing only "users", with the appropriate caveats that this is no longer a randomized sample (see 18-i).

	1	2	3	4	5	
subitem not at all important	○	○	○	○	○	essential

Does your paper address subitem 16-ii?

Copy and paste relevant sections from the manuscript (include quotes in quotation marks "like this" to indicate direct quotes from your manuscript), or elaborate on this item by providing additional information not in the ms, or briefly explain why the item is not applicable/relevant for your study

Jouw antwoord

17a) For each primary and secondary outcome, results for each group, and the estimated effect size and its precision (such as 95% confidence interval)

Does your paper address CONSORT subitem 17a? *

Copy and paste relevant sections from the manuscript (include quotes in quotation marks "like this" to indicate direct quotes from your manuscript), or elaborate on this item by providing additional information not in the ms, or briefly explain why the item is not applicable/relevant for your study

See Table 2 for the results of the WDYD intervention compared to the control group for each primary and secondary outcome, including the model coefficients (B, SE, 95%CI) and effect sizes.

17a-i) Presentation of process outcomes such as metrics of use and intensity of use

In addition to primary/secondary (clinical) outcomes, the presentation of process outcomes such as metrics of use and intensity of use (dose, exposure) and their operational definitions is critical. This does not only refer to metrics of attrition (13-b) (often a binary variable), but also to more continuous exposure metrics such as “average session length”. These must be accompanied by a technical description how a metric like a “session” is defined (e.g., timeout after idle time) [1] (report under item 6a).

	1	2	3	4	5	
subitem not at all important	○	○	○	○	○	essential

Does your paper address subitem 17a-i?

Copy and paste relevant sections from the manuscript (include quotes in quotation marks "like this" to indicate direct quotes from your manuscript), or elaborate on this item by providing additional information not in the ms, or briefly explain why the item is not applicable/relevant for your study

Jouw antwoord

17b) For binary outcomes, presentation of both absolute and relative effect sizes is recommended

Does your paper address CONSORT subitem 17b? *

Copy and paste relevant sections from the manuscript (include quotes in quotation marks "like this" to indicate direct quotes from your manuscript), or elaborate on this item by providing additional information not in the ms, or briefly explain why the item is not applicable/relevant for your study

See Table 2 for the model coefficients and effect sizes of the binary outcomes (excessive and binge drinking)

18) Results of any other analyses performed, including subgroup analyses and adjusted analyses, distinguishing pre-specified from exploratory

Does your paper address CONSORT subitem 18? *

Copy and paste relevant sections from the manuscript (include quotes in quotation marks "like this" to indicate direct quotes from your manuscript), or elaborate on this item by providing additional information not in the ms, or briefly explain why the item is not applicable/relevant for your study

"Small differences were found in drinking behavior by recruitment strategy. Participants reporting higher weekly alcohol consumption were more likely recruited via social media ($\exp(\beta) = 1.01$, $P < .001$) and less likely via teachers ($\exp(\beta) = 0.95$, $P < .001$) or the lifestyle monitor ($\exp(\beta) = 0.92$, $P < .001$). No other significant baseline differences were observed on drinking behavior, and no interaction effects of recruitment strategy with group assignment were found."

"We did conduct additional moderation analyses, and found no evidence of educational attainment moderating the treatment effect (data not shown)."

"Third, additional analyses revealed that increases in intrinsic motivation and self-confidence among active users of WDYD were significantly associated with reductions in alcohol consumption (data not shown)."

"A small proportion of participants were recruited via channels that may have provided prior exposure to alcohol-related information, such as the online lifestyle monitor (5.3%) or educational programs delivered by teachers (4.4%). Although this may have reduced contrast between intervention and control conditions, moderation analyses showed that recruitment method did not significantly influence group effects (data not shown)."

18-i) Subgroup analysis of comparing only users

A subgroup analysis of comparing only users is not uncommon in ehealth trials, but if done, it must be stressed that this is a self-selected sample and no longer an unbiased sample from a randomized trial (see 16-iii).

	1	2	3	4	5	
subitem not at all important	○	○	○	○	○	essential

Does your paper address subitem 18-i?

Copy and paste relevant sections from the manuscript (include quotes in quotation marks "like this" to indicate direct quotes from your manuscript), or elaborate on this item by providing additional information not in the ms, or briefly explain why the item is not applicable/relevant for your study

Jouw antwoord

19) All important harms or unintended effects in each group

(for specific guidance see CONSORT for harms)

Does your paper address CONSORT subitem 19? *

Copy and paste relevant sections from the manuscript (include quotes in quotation marks "like this" to indicate direct quotes from your manuscript), or elaborate on this item by providing additional information not in the ms, or briefly explain why the item is not applicable/relevant for your study

There were no harms or unintended effects of the WDYD intervention found. See Table 2 for the effects.

19-i) Include privacy breaches, technical problems

Include privacy breaches, technical problems. This does not only include physical “harm” to participants, but also incidents such as perceived or real privacy breaches [1], technical problems, and other unexpected/unintended incidents. “Unintended effects” also includes unintended positive effects [2].

	1	2	3	4	5	
subitem not at all important	○	○	○	○	○	essential

Does your paper address subitem 19-i?

Copy and paste relevant sections from the manuscript (include quotes in quotation marks "like this" to indicate direct quotes from your manuscript), or elaborate on this item by providing additional information not in the ms, or briefly explain why the item is not applicable/relevant for your study

Jouw antwoord

19-ii) Include qualitative feedback from participants or observations from staff/researchers

Include qualitative feedback from participants or observations from staff/researchers, if available, on strengths and shortcomings of the application, especially if they point to unintended/unexpected effects or uses. This includes (if available) reasons for why people did or did not use the application as intended by the developers.

	1	2	3	4	5	
subitem not at all important	○	○	○	○	○	essential

Does your paper address subitem 19-ii?

Copy and paste relevant sections from the manuscript (include quotes in quotation marks "like this" to indicate direct quotes from your manuscript), or elaborate on this item by providing additional information not in the ms, or briefly explain why the item is not applicable/relevant for your study

Jouw antwoord

DISCUSSION

22) Interpretation consistent with results, balancing benefits and harms, and considering other relevant evidence

NPT: In addition, take into account the choice of the comparator, lack of or partial blinding, and unequal expertise of care providers or centers in each group

22-i) Restate study questions and summarize the answers suggested by the data, starting with primary outcomes and process outcomes (use)

Restate study questions and summarize the answers suggested by the data, starting with primary outcomes and process outcomes (use).

	1	2	3	4	5	
subitem not at all important	○	○	○	○	○	essential

Does your paper address subitem 22-i? *

Copy and paste relevant sections from the manuscript (include quotes in quotation marks "like this" to indicate direct quotes from your manuscript), or elaborate on this item by providing additional information not in the ms, or briefly explain why the item is not applicable/relevant for your study

"This study evaluated the effect, use and acceptability of a dynamically tailored mobile intervention WDYD to reduce excessive alcohol drinking among at-risk adolescents and young adults. Regarding the primary outcomes, we found no support for our hypothesis that WDYD was more effective than the control group in reducing alcohol consumption. No effect of WDYD was found on mean weekly alcohol consumption. Positive effects of WDYD were found only in the first week for excessive drinking. In addition, there was an increase in binge drinking in the first week in both groups, but this increase was significantly smaller in the intervention group. After this first week, alcohol consumption seemed to decrease over time in both groups, but differences between groups were non-significant at all time-points. The increase in binge drinking in the first week was possibly due to the measurement difference between baseline and the following weeks. At baseline, alcohol consumption was based on a participants' self-reported alcohol intake during a typical week, whereas at the follow-up measurements, participants were asked to report their consumption of the day before (1-day recall) for seven consecutive days. A 1-day recall leads to more accurate estimates than a 7-day recall because it is less susceptible to recall bias [52], and this difference is even more pronounced with binge drinking [42].
The reduction in alcohol consumption over time in both groups in this study is in contrast with the study about a previous web-based version of WDYD [29], which showed that both groups increased their alcohol consumption over time, with the intervention group showing a significant lower increase than the control group. Differences between this study and the previous study may partly explain the variation of observed effects. The previous study used a Web-based brief, single-session tailored feedback intervention. Effects were measured during 30 weekly 7-day recall EMAs over a 6 month period among a high educated sample of young adults (i.e., students in higher professional education or university) recruited via flyers and included when they were ready to change their alcohol consumption. In this study, the reduction of alcohol consumption in both groups over time as well as the absence of a significant intervention effect on alcohol consumption could be explained by the use of daily 1-day recall EMAs; the study participants, including those in the control group, self-monitored their alcohol drinking behavior daily for seven weeks during the study. Self-monitoring is shown to be an important behavior change technique in predicting positive outcomes regarding alcohol reduction research [14, 23]. Therefore, it is highly likely that the difference in outcomes between groups would have been greater if the control group had engaged in less frequent self-monitoring. Moreover, a difference between the studies lies in the inclusion criteria. First, the two studies targeted different populations. The previous study focused on highly educated young adults (i.e., students in higher professional education and university), whereas this study primarily targeted adolescents and young adults from secondary vocational education and expanded recruitment to a broader group of Dutch adolescents and young adults. These populations differ in educational level, lifestyle context, and potentially in digital literacy, which may influence engagement and responsiveness to tailored feedback. Second, the previous study included participants who were already ready to change their alcohol consumption [29], a factor known to predict intervention responsiveness and behavior change outcomes [53, 54]. In contrast, this study did not preselect participants based on their readiness to change, resulting in a more heterogeneous sample in terms of readiness. This combination of a different target group and broader variation in readiness may have reduced intervention effects compared to the earlier trial.
This study shows that there was large variation between individuals on alcohol use and its determinants over time. This is in line with earlier research [29, 55], and supports the dynamically tailored nature of WDYD. At the same time, it emphasizes a need for additional data-analyses besides evaluation of group-level effects. Specifically, an analysis method that accounts for differences between and within individuals over time in order to learn why and for whom the intervention is effective and to improve future dynamically tailored

[illegible] interventions [56].
An important flaw of the study was the high dropout rates, with 63.1% within the first week rising to 96.0% at the final week 33, even though we applied several strategies to prevent dropout (e.g. rewards, therapeutic alliance, dynamic tailoring; [10, 21]. Dropout was higher among students in secondary vocational education and those who consumed more glasses of alcohol weekly. High drop-out is a common problem in mHealth interventions, especially in the first two weeks of the study and among those most at-risk, and this needs attention (e.g., [10, 57]). EMA is a promising means to provide dynamic tailoring, however, it also places cognitive burden upon participants and difficulties in keeping participants engaged [21]. Although sensor technology may be a promising strategy to reduce this burden, this is not yet available, reliable or practical for the outcomes measured in this study [58]. WDYD was a stand-alone intervention and did not use in-person contact. Both in-person contact (e.g., with a counselor) or a combined or stepped-care intervention (e.g., mHealth combined with an educational program at school) could be promising ways to reduce dropout while also improving effectiveness [41, 59, 60]. In addition, although WDYD included pictures, videos and a design that was tailored to accommodate low literacy levels, it relied on text to provide tailored feedback. Since health literacy is related to dropout, future mHealth interventions could rely more on audio and video feedback [59]. Also, Providing tailored information by a conversational agent using generative Artificial Intelligence (AI) could be a promising way to reduce dropout and increase intervention effects [61, 62]. Possibly, the WDYD intervention was too static, thereby limiting engagement. Emerging developments in generative AI offer additional opportunities to personalize interventions at scale. AI-driven conversational agents can deliver dynamic, context-aware feedback, simulate motivational interviewing, and adapt content based on real-time user data. These technologies could enhance engagement, reduce attrition, and provide more nuanced support for behavior change compared to static tailoring. However, ethical considerations such as privacy, transparency, and bias mitigation must be addressed before widespread implementation in mHealth interventions.
Despite not finding significant effects on the primary outcomes, and dropout being a serious problem, WDYD could be seen as a promising intervention. This is especially true when taking into account that young and lower educated adolescents and young adults are a difficult group to reach, as the majority (90%) lack motivation to reduce their alcohol consumption [29]. First, the combination of recruitment strategies used in this study showed that we are able to reach young adults who drink excessively; the use of social media advertisements resulted in the largest number of participants. Second, we found positive effects of WDYD on the secondary outcomes intrinsic motivation and self-confidence towards drinking less alcohol. Both outcomes significantly increased in the intervention group compared to the control group, and this effect lasted over 25 weeks. Third, additional analyses revealed that increases in intrinsic motivation and self-confidence among active users of WDYD were significantly associated with reductions in alcohol consumption (data not shown). WDYD can thus be an effective means to improve motivation and self-confidence towards reducing alcohol consumption, and this increase seems to have promising effects on alcohol consumption itself. In addition, the user-based assessments revealed that the intervention was evaluated very positively. WDYD was evaluated as usable (e.g., user-friendly, easy in navigation), credible, understandable, and providing the adequate amount of information. The informativity and usefulness were evaluated as sufficient. Dynamic tailoring was successfully implemented in the intervention design, as participants indicated that WDYD accounted for their personal preferences, discussed topics they found important, and provided freedom in choosing their alcohol consumption. This was also the case for Motivational Interviewing since participants felt accepted for who they are and felt addressed in a pleasant way.
A small proportion of participants were recruited via channels that may have provided prior

exposure to alcohol-related information, such as the online lifestyle monitor (5.3%) or educational programs delivered by teachers (4.4%). Although this may have reduced contrast between intervention and control conditions, moderation analyses showed that recruitment method did not significantly influence group effects (data not shown)."

22-ii) Highlight unanswered new questions, suggest future research

Highlight unanswered new questions, suggest future research.

	1	2	3	4	5	
subitem not at all important	○	○	○	○	○	essential

Does your paper address subitem 22-ii?

Copy and paste relevant sections from the manuscript (include quotes in quotation marks "like this" to indicate direct quotes from your manuscript), or elaborate on this item by providing additional information not in the ms, or briefly explain why the item is not applicable/relevant for your study

Jouw antwoord

20) Trial limitations, addressing sources of potential bias, imprecision, and, if relevant, multiplicity of analyses

20-i) Typical limitations in ehealth trials

Typical limitations in ehealth trials: Participants in ehealth trials are rarely blinded. Ehealth trials often look at a multiplicity of outcomes, increasing risk for a Type I error. Discuss biases due to non-use of the intervention/usability issues, biases through informed consent procedures, unexpected events.

	1	2	3	4	5	
subitem not at all important	○	○	○	○	○	essential

Does your paper address subitem 20-i? *

Copy and paste relevant sections from the manuscript (include quotes in quotation marks "like this" to indicate direct quotes from your manuscript), or elaborate on this item by providing additional information not in the ms, or briefly explain why the item is not applicable/relevant for your study

"This study had multiple limitations. First and foremost, dropout rates were high, particularly in the intervention group. This selective attrition may have introduced bias. To mitigate this, we applied multiple imputation [63]. However, due to difficulties imputing data from weeks 31 and 33 after baseline, these time points were omitted from the imputation model, and analyses were limited to data up to 25 weeks. Additionally, we did not examine reasons for dropout, which could have provided important information to reduce dropout in the future [59].

Second, although the intervention was originally developed for students in secondary vocational education, recruitment challenges let us to broaden our inclusion criteria to all Dutch adolescents and young adults engaging in excessive drinking. As a result, only 34.6% of the sample consisted of students in secondary vocational education, and 6.0% had a low educational level. While moderation analyses showed no significant interaction between educational level and treatment effects (data not shown), caution is warranted when generalizing results to students in secondary vocational education or students from lower-educated populations. Sampling from a more diverse population may have introduced more variability in drinking behavior and decreased receptiveness to our intervention, which could affect the interpretation of our outcomes.

Third, a small proportion of participants (5.3%) were recruited via an online lifestyle monitor or via teachers delivering alcohol education (4.4%). These participants may have already been exposed to normative feedback or alcohol-related risk information prior to the study. This prior exposure could have reduced the contrast between the intervention and control conditions, potentially reducing observed effects.

Fourth, we used single-item measures to assess the primary and secondary outcomes. Although these items were selected based on prior studies and some of these items showed comparable or adequate performance compared to multi-item scales [44-46], single items measures are generally more susceptible to measurement error and have lower psychometric quality [64]. In addition, the items used were not validated prior to our study, which may limit the reliability and generalizability of our findings. However, in studies using EMA, multiple-item scales may enhance reactivity and participant burden [65], justifying our choice for brevity.

Finally, there was a potential risk of contamination between conditions. Although participants were individually randomized into the control or experimental condition within the WDYD app, it is possible that participants in the control group were acquainted with those in the intervention group and discussed intervention content. To assess this, we asked participants in the control group 12 questions about the intervention at the first follow-up (9 weeks after baseline). The answers provided no strong indication of contamination. The distribution of correct answers resembled random guessing, and no participant answered more than 8 out of 12 items correctly (data not shown). "

21) Generalisability (external validity, applicability) of the trial findings

NPT: External validity of the trial findings according to the intervention, comparators, patients, and care providers or centers involved in the trial

21-i) Generalizability to other populations

Generalizability to other populations: In particular, discuss generalizability to a general Internet population, outside of a RCT setting, and general patient population, including applicability of the study results for other organizations

	1	2	3	4	5	
subitem not at all important	○	○	○	○	○	essential

Does your paper address subitem 21-i?

Copy and paste relevant sections from the manuscript (include quotes in quotation marks "like this" to indicate direct quotes from your manuscript), or elaborate on this item by providing additional information not in the ms, or briefly explain why the item is not applicable/relevant for your study

Jouw antwoord

21-ii) Discuss if there were elements in the RCT that would be different in a routine application setting

Discuss if there were elements in the RCT that would be different in a routine application setting (e.g., prompts/reminders, more human involvement, training sessions or other co-interventions) and what impact the omission of these elements could have on use, adoption, or outcomes if the intervention is applied outside of a RCT setting.

	1	2	3	4	5	
subitem not at all important	○	○	○	○	○	essential

Does your paper address subitem 21-ii?

Copy and paste relevant sections from the manuscript (include quotes in quotation marks "like this" to indicate direct quotes from your manuscript), or elaborate on this item by providing additional information not in the ms, or briefly explain why the item is not applicable/relevant for your study

Jouw antwoord

OTHER INFORMATION

23) Registration number and name of trial registry

Does your paper address CONSORT subitem 23? *

Copy and paste relevant sections from the manuscript (include quotes in quotation marks "like this" to indicate direct quotes from your manuscript), or elaborate on this item by providing additional information not in the ms, or briefly explain why the item is not applicable/relevant for your study

Abstract: "Trial Registration: Onderzoekmetmensen.nl Trial 28135; https://onderzoekmetmensen.nl/nl/trial/28135"

Methods: "Trial Registration: onderzoekmetmensen.nl Trial 28135 [17, 18])"

24) Where the full trial protocol can be accessed, if available

Does your paper address CONSORT subitem 24? *

Cite a Multimedia Appendix, other reference, or copy and paste relevant sections from the manuscript (include quotes in quotation marks "like this" to indicate direct quotes from your manuscript), or elaborate on this item by providing additional information not in the ms, or briefly explain why the item is not applicable/relevant for your study

The trial protocol can be accessed via: "Trial registration: Onderzoekmetmensen.nl Trial 28135; https://onderzoekmetmensen.nl/nl/trial/28135"

25) Sources of funding and other support (such as supply of drugs), role of funders

Does your paper address CONSORT subitem 25? *

Copy and paste relevant sections from the manuscript (include quotes in quotation marks "like this" to indicate direct quotes from your manuscript), or elaborate on this item by providing additional information not in the ms, or briefly explain why the item is not applicable/relevant for your study

"The study is funded by the Netherlands Organisation for Health Research and Development (ZonMw #531001105). The funder had no involvement in the study design, data collection, analysis, interpretation or the writing of the manuscript."

X27) Conflicts of Interest (not a CONSORT item)

X27-i) State the relation of the study team towards the system being evaluated

In addition to the usual declaration of interests (financial or otherwise), also state the relation of the study team towards the system being evaluated, i.e., state if the authors/evaluators are distinct from or identical with the developers/sponsors of the intervention.

	1	2	3	4	5	
subitem not at all important	○	○	○	○	○	essential

Does your paper address subitem X27-i?

Copy and paste relevant sections from the manuscript (include quotes in quotation marks "like this" to indicate direct quotes from your manuscript), or elaborate on this item by providing additional information not in the ms, or briefly explain why the item is not applicable/relevant for your study

Jouw antwoord

About the CONSORT EHEALTH checklist

As a result of using this checklist, did you make changes in your manuscript? *

- ○ yes, major changes
- ◉ yes, minor changes
- ○ no

What were the most important changes you made as a result of using this checklist?

Added details about study procedure)

How much time did you spend on going through the checklist INCLUDING making changes in your manuscript *

A lot, in total for the first time 5 days.

As a result of using this checklist, do you think your manuscript has improved? *

- ◉ yes
- ○ no
- ○ Anders:

Would you like to become involved in the CONSORT EHEALTH group?

This would involve for example becoming involved in participating in a workshop and writing an "Explanation and Elaboration" document

- ○ yes
- ○ no
- ○ Anders:

Any other comments or questions on CONSORT EHEALTH

Jouw antwoord

STOP - Save this form as PDF before you click submit

To generate a record that you filled in this form, we recommend to generate a PDF of this page (on a Mac, simply select "print" and then select "print as PDF") before you submit it.

When you submit your (revised) paper to JMIR, please upload the PDF as supplementary file.

Don't worry if some text in the textboxes is cut off, as we still have the complete information in our database. Thank you!

Final step: Click submit !

Click submit so we have your answers in our database!

Verzenden   Formulier wissen

Verzend nooit wachtwoorden via Google Formulieren.

Deze content is niet gemaakt of goedgekeurd door Google. - Contact opnemen met eigenaar van formulier - Servicevoorwaarden - Privacybeleid

Ziet dit formulier er verdacht uit? Rapport

Google Formulieren
